# A network pharmacology-based strategy deciphers the underlying molecular mechanisms of Qixuehe Capsule in the treatment of menstrual disorders

**DOI:** 10.1186/s13020-017-0145-x

**Published:** 2017-08-21

**Authors:** Yanqiong Zhang, Xia Mao, Jing Su, Ya Geng, Rui Guo, Shihuan Tang, Junfang Li, Xuefeng Xiao, Haiyu Xu, Hongjun Yang

**Affiliations:** 1 Institute of Chinese Materia Medica, China Academy of Chinese Medical Sciences, No. 16, Nanxiaojie, Dongzhimennei, Beijing, 100700 China; 2 School of Basic Medicine, Shandong University of Traditional Chinese Medicine, Jinan, 250300 China; 3 College of Pharmacy, Tianjin University of Traditional Chinese Medicine, Tianjin, 300193 China

**Keywords:** Traditional Chinese Medicine, Chinese herbal formula, Menstrual disorders, Network pharmacology, Molecular docking simulation

## Abstract

**Background:**

QiXueHe Capsule (QXHC) is a Chinese patent drug that is extensively used for the treatment of menstrual disorders. However, its underlying pharmacological mechanisms have not been fully elucidated.

**Methods:**

A list of QXHC putative targets were predicted using MetaDrug. An interaction network using links between QXHC putative targets and the known therapeutic targets of menstrual disorders was constructed. QXHC candidate targets were also identified via calculating the topological feature values of nodes in the network. Additionally, molecular docking simulation was performed to determine the binding efficiency of QXHC compound-putative target pairs.

**Results:**

A total of 1022 putative targets were predicted for 311 chemical components containing in QXHC. Following the calculation of topological features of QXHC putative target-known therapeutic target of menstrual disorder network, 66 QXHC candidate targets for the treatment of menstrual disorders were identified. Functionally, QXHC candidate targets were significantly associated with several biological pathways, such as VEGF and Chemokine signaling pathways, Alanine/aspartate/glutamate metabolism, Long-term depression and T/B cell receptor signaling pathway. Moreover, molecular docking simulation demonstrated that there were 20 pairs of QXHC chemical component-candidate target had the strong binding free energy.

**Conclusions:**

This novel and scientific network pharmacology-based study holistically deciphers that the pharmacological mechanisms of QXHC in the treatment of menstrual disorders may be associated with its involvement into hemopoiesis, analgesia, nutrients absorption and metabolism, mood regulation, as well as immune modulation.

**Electronic supplementary material:**

The online version of this article (doi:10.1186/s13020-017-0145-x) contains supplementary material, which is available to authorized users.

## Background

Menstrual disorders, including painful cramps during bleeding, abnormally heavy bleeding, or not having any bleeding, are problems which may affect the normal menstrual cycle of females [1]. Recent epidemiologic studies have declared its high prevalent rates, such as secondary amenorrhea (2.6–8.5%), irregular menstruations (11.3–26.7%) and dysmenorrheal (50%) in adult females [2–5]. Clinically, abnormal uterine bleeding accounts for nearly 20% of outpatient visits and 25% of gynecology-related operations, which may seriously influence the quality of life, mental state and even future fertility of females [6, 7]. Regular menstruation is the periodic shedding of endometrium and blood from the uterus after monthly ovulation, which is regulated by the neuroendocrine hypothalamic-pituitary-ovary axis [8]. Any apparent changes in menstruation patterns are considered as menstrual disorders [9]. Some lifestyle factors could exert a continued influence on the abnormal bleeding of uterus, such as body weight, food habits, physical activities and delivery types (normal vaginal delivery and caesarian section) [9, 10]. Organic pathological changes including polycystic ovary, endometriosis, hypogonadism and even tumors also lead to menstrual disorders [11]. In the current clinics, there exist several medical treatment or surgical options in treating various patterns of menstrual disorders, aiming to adjust the neuroendocrine hypothalamic-pituitary-ovary axis. First-line medical treatment for menstrual disorders include luteal-phase progestins, danazol, tranexamic acid and hormone-releasing intrauterine system [12]. If medications fail or are not well received by patients, the conservative surgery (endometrial ablation and laparoscopic surgery), hysterectomy and developing molecular-targeted therapy may be performed [13]. Although significant improvements in patients’ outcome have been achieved in recent years, there have been a number of potential side effects of the conventional therapeutic strategies which are used in the treatment of menstrual disorders. For example, some patients may experience syndromes including breast tenderness, weight gain, nausea, tiredness and irregular vagina bleeding after receiving the conventional treatment. Moreover, there also exist high risks of disease recurrence or exacerbation [14]. Therefore, more effective and safer therapeutic approaches are urgently demanded for the patients with menstrual disorders.

Traditional Chinese medicine (TCM), as an important part of complementary and alternative medical systems, is characterized by comprehensive medical effects and has been extensively used in clinical practice for thousands of years in Asian countries, especially in China, Japan, North and South Korea [15]. A classic literature titled “*The Inner Canon of Huangdi*” has described the origin and formation of menstrual disorders in ancient China. In TCM theory, the generation and regulation of menstruation are closely related to the functions of liver (blood storage and *“qi”* regulation), spleen (source of blood and *“qi”*; digestion and absorption of nutrients) and kidney (essence storage and the root of congenital constitution) systems. The dysfunctions of the three systems accompanying with the deficiency of blood and *“qi”*, and meridian congestion may result in the occurrence of menstrual disorders [16]. Several Chinese herbal formulae and patent drugs are extensively used for the treatment of abnormal bleeding of uterus in clinics. Among them, QiXueHe Capsule (QXHC) is a commonly used multi-herb Chinese patent drug with a satisfactory efficacy, and is prepared under the principle of promoting blood circulation and regulating *“qi”* circulation. It consists of fifteen Chinese herbs including the principle herbs *Angelica sinensis* (Danggui, DG) and *Radix Paeoniae Rubra* (Chishao, CS); the ministerial herbs *Ligusticum wallichii* (Chuanxiong, CX), seed of *Prunus persica*(L.)*Batsch* (Taoren, TR), *Carthamus tinctorius* (Honghua, HH), *Radix Bupleuri* (Chaihu, CH), *Cyperus rotundus* (Xiangfu, XF), *Salvia miltiorrhiza* (Danshen, DS), *Rhizoma Corydalis* (Yanhusuo, YHS) and *Platycodon grandiflorum* (Jigen, JG); the adjunctive herbs *Fructus Aurantii* (Zhiqiao, ZQ), *Lindera aggregate* (Wuyao, WY) and *Achyranthes bidentata* (Niuxi, NX); the messenger herbs *Rhizoma Cimicifugae* (Shenma, SM) and *Glycyrrhiza uralensis* (Gancao, GC). Among them, DG, CS and CX excel in nourishing blood and promoting its circulation, accompanied by TR and HH to remove blood stasis [17–19]. DS, YHS and JG can activate the circulation of blood and *“qi”* to alleviate pain, on the basis of a TCM theory “when there is stagnation, there is pain, and vice versa” [20, 21]. Moreover, the herbal pair CH and XF act on liver for alleviating *“qi”* stagnation, as well as, ZQ and WY/NX combination, respectively, exert effects on spleen and kidney to reinforce their functions [22–24]. The messenger herbs SM and GC play a role in guiding the actions of all herbs to a certain area of the human body, and harmonizing and integrating the effects of all herbs in this formula [25, 26]. Growing evidence shows the pharmacological and biochemical actions of the chemical components containing in QXHC. For example, Z-ligustilide in DG, total glycosides in CS, tanshinone IIA and phenolic acids in DS, and licoricidin in GC, may exert obvious anti-inflammatory, anti-tumor and anti-hepatotoxic effects [27–30]. However, the underlying pharmacological mechanisms of QXHC acting on menstrual disorders have not been fully elucidated due to a lack of appropriate research approaches to Chinese herbal formulae.

**Fig. 1:**
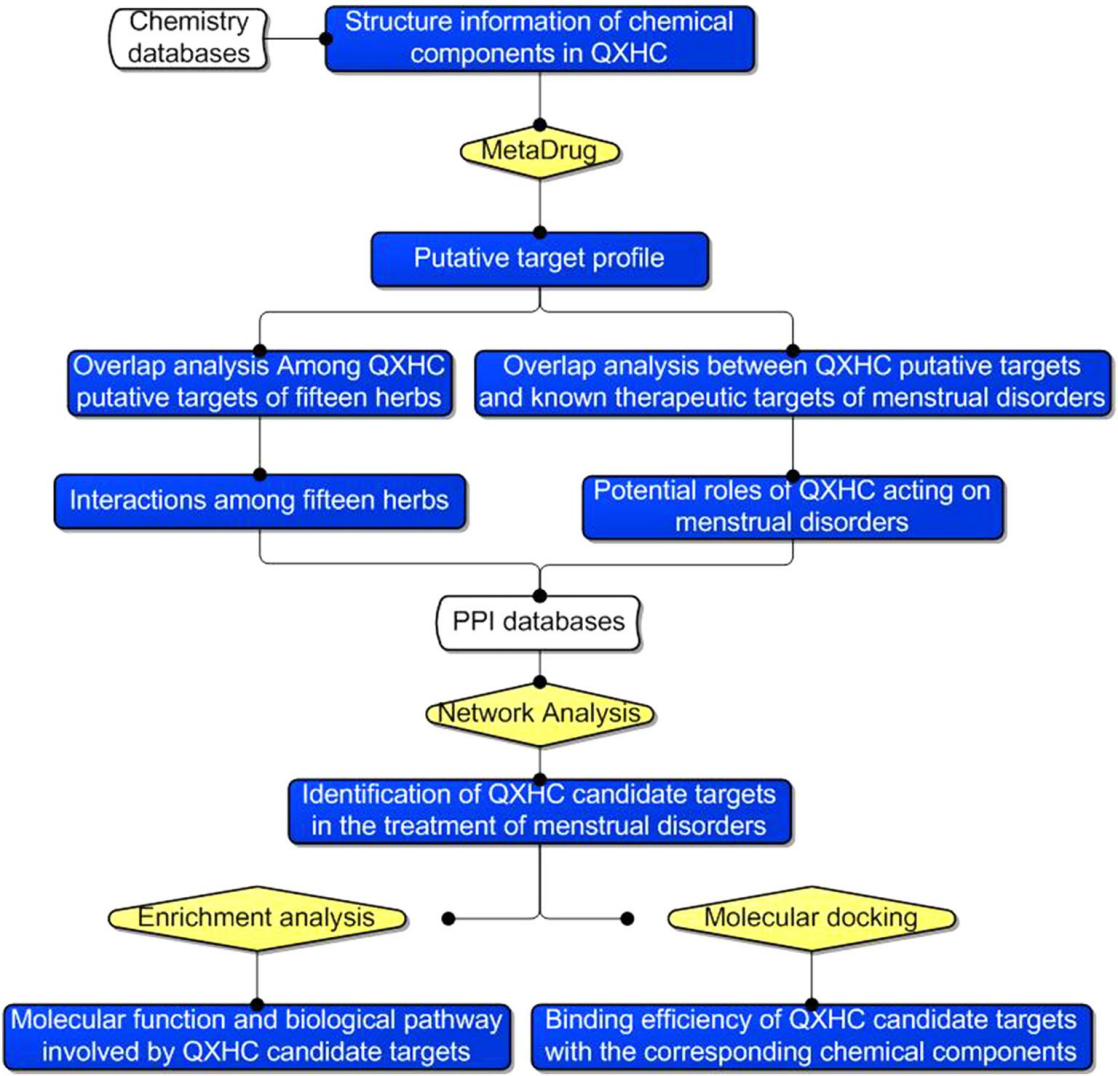
A schematic diagram of the network pharmacology-based strategies for unraveling the pharmacological mechanisms of QXHC acting on menstrual disorders

Network pharmacology, firstly proposed by Hopkins AL, has become a powerful tool in elucidating complex and holistic mechanisms of TCM with the rapid progress of systems biology, bioinformatics and polypharmacology [31–34]. It illustrates the intricate interactions among genes, proteins and metabolites related to diseases and drugs from a network perspective, which is consistent with the multi-component and multi-target nature of TCM. The integration of network pharmacology and TCM are shifting the conventional “one target, one drug” paradigm to “multi-target, multi-component drug” strategy [35]. In recent years, our research groups have identified the candidate drug target of various TCM herbal formulae acting on the corresponding diseases using a series network pharmacology-based strategies. To investigate the underlying pharmacological mechanisms of QXHC, we here performed a three-step analysis: (1) Predicting QXHC putative targets; (2) Illustrating a drug target-disease gene network using the interactions between QXHC putative targets and the known therapeutic targets of menstrual disorders-related diseases, and identifying the QXHC candidate targets for the treatment of menstrual disorders; (3) Validating the binding efficiency of chemical components containing in QXHC to the corresponding candidate targets using molecular docking simulation. Figure 1 depicts a flowchart of the technical strategy used in this study.

## Methods

The Minimum Standards of Reporting Checklist contains details of the experimental design, and statistics, and resources used in this study (Additional file 1).

### Data preparation

#### Chemical components of each herb containing in QXHC

Chemical components of each herb containing in QXHC were obtained from a chemistry database [36] (http://www.organchem.csdb.cn/scdb/main/slogin.asp, updated on 2014-05-05), which is specialized in storing chemical related information, including chemical and crystal structures, spectra, reactions, syntheses, as well as thermophysical data. In total, we collected the structural information of 54 components for CH, 8 components for CS, 28 components for CX, 29 components for DS, 61 components for DG, 50 components for GC, 28 components for HH, 15 components for JG, 20 components for NX, 8 components for SM, 17 components for TR, 6 components for WY, 11 components for XF, 13 components for YHS and 28 components for ZQ. The molecular files of all the chemical components were downloaded from ChemSpider (http://www.chemspider.com/, updated on 2011-12-23) and saved in *.sdf format.

#### Known therapeutic targets for the treatment of menstrual disorders

Known therapeutic targets for the treatment of menstrual disorders were obtained from the DrugBank database [37] (http://www.drugbank.ca/, version, 4.3). We only selected the drug-target interactions whose drugs are approved by the Food and Drug Administration, USA (FDA) for treating menstrual disorders. All target gene/protein identifiers (IDs) were converted into the correspondinggene symbol/UniProtKB-Swiss-Prot IDto facilitate further data analyses. After removing redundant entries, 37 known therapeutic targets for the treatment of menstrual disorders were collected. Detailed information about these known therapeutic targets is provided in Additional file 2: Table S1.

#### Protein-protein interaction (PPI) data

PPI data were imported from the following PPI databases, including Human Annotated and Predicted Protein Interaction Database (HAPPI, http://bio.informatics.iupui.edu/HAPPI/, Version 31.2) [38], Reactome (http://www.reactome.org/, Version 37) [39], Online Predicted Human Interaction Database(OPHID, http://ophid.utoronto.ca, Version 1.71) [40], InAct (http://www.ebi.ac.uk/intact/, Version 2.0) [41], Human Protein Reference Database (HPRD, http://www.hprd.org/, Release 8) [42], Molecular interaction Database (MINT, http://mint.bio.uniroma2.it/mint/download.do, Aug-2011) [43], Database of Interacting Proteins (DIP, http://dip.doe-mbi.ucla.edu/dip/, Jan-2010) [44] and PDZBase (http://icb.med.cornell.edu/services/pdz/start, 2004) [45].

### Prediction of QXHC putative targets

The putative targets of chemical components containing in QXHC were predicted by MetaDrug from GeneGo, Inc. which combines chemical structural analysis tools, a structure-activity database, and a systems biology database of molecular interactions, canonical signaling and metabolic pathways, and gene-biological property associations [46, 47]. Putative targets of certain chemical components were predicted by comparing the structure of the certain chemical components to that of known drugs. The targets of known drugs with high structural similarity (the structural similarity score is higher than 0.8) were identified as the putative targets of the certain chemical components.

### Gene Ontology (GO) and pathway enrichment analyses

GO and pathway enrichment analyses were performed using the application of database for Annotation, Visualization and Integrated Discovery [48] (DAVID, http://david.abcc.ncifcrf.gov/home.jsp, version 6.7), on the basis of the information obtained from GO (http://www.geneontology.org) [49] and KEGG pathway database [50] (Kyoto Encyclopedia of Genes and Genomes, http://www.genome.jp/kegg/, Last updated, Oct 16, 2012), respectively.

### QXHC putative targets-known therapeutic targets for menstrual disorders interaction network construction and analysis

QiXueHe Capsule putative targets-known therapeutic targets for menstrual disorders interaction network (drug target-known disease therapeutic targets network)was constructed using the links among QXHC putative targets and known therapeutic targets for menstrual disorders. The PPI data were collected from eight existing PPI databases as listed above [38–45]. The network was visualized by Navigator software (version 2.2.1).

Then, the nodes, the degree of which are higher than the median value of the degree of all nodes in the network, were identified as hubs. After that, the hub network was constructed using the direct interactions among hubs. To identify the major hubs, 4 topological features of nodes, including ‘Degree’, ‘Node-betweenness’, ‘Closeness’, and ‘K-value’, were calculated. The definitions of the above topological features have been described in our previous studies [51–53]. Hubs, the four topological feature values of which are all higher than the corresponding median values, were identified as major hubs in the hub network.

### Molecular docking simulation

Molecular docking simulation was carried out to evaluate the binding efficiency of QXHC candidate targets with the corresponding chemical components containing in QXHC using the program LibDock implemented in Discovery Studio (Accelrys, San Diego, CA). We collected all the crystal structures of QXHC candidate targets from the RCSB protein data bank (PDB, http://www.pdb.org/, updated on 2014-3-11) and selected the relatively higher resolution crystal structures with the ligands. The virtual screening protocol LibDock was used to calculate the docking scores for binding efficiency assessment. Compounds to be docked were prepared via the Prepare Ligand protocol to give 3D coordinates and confirmation. Number of Hotspots generated by LibDock was set as 100 for each case while the remaining parameters were unchanged. After docking, binding poses of compounds were assessed by LibDock Score and visual inspection to identify the correct poses. The pairs of target-component which had higher docking score than 100 (the median value of all docking scores) were indicated that these QXHC candidate targets had strong binding efficiency with the corresponding chemical components.

## Results

### Putative target profile of QXHC

A total of 1022 putative targets were predicted for 311 chemical components of 15 herbs containing in QXHC, including 261 putative targets for CH, 78 for CS, 352 for CX, 664 for DG, 211 for DS, 286 for GC, 263 for HH, 65 for JG, 384 for NX, 64 for SM, 258 for TR, 106 for WY, 96 for XF, 84 for YHS and 188 for ZQ (Additional file 2: Table S2). Among them, 31 putative targets of QXHC have been identified as the known therapeutic targets for the treatment of menstrual disorders according to the data obtained from the DrugBank database. Among 15 herbs containing in QXHC, CS (functions as a principle herb in QXHC and plays a role in promotion of blood circulation to remove blood stasis), XF (functions as a ministerial herb, and can relieve “*qi*” stagnation in liver), YHS (functions as a ministerial herb, and plays a role in the regulation of “*qi*”), JG (functions as an adjunctive herb, and can tonify “*qi*”) and SM (functions as a messenger herb, and invigorates “*qi*”) shared the most common potential targets (more than 50% of their putative targets) with the other herbs, suggesting that CS, XF, YHS, JG and SM may link with other herbs more closely.

### Pathways involved by QXHC putative targets consistent with the therapeutic effects of the corresponding herbs

**Fig. 2:**
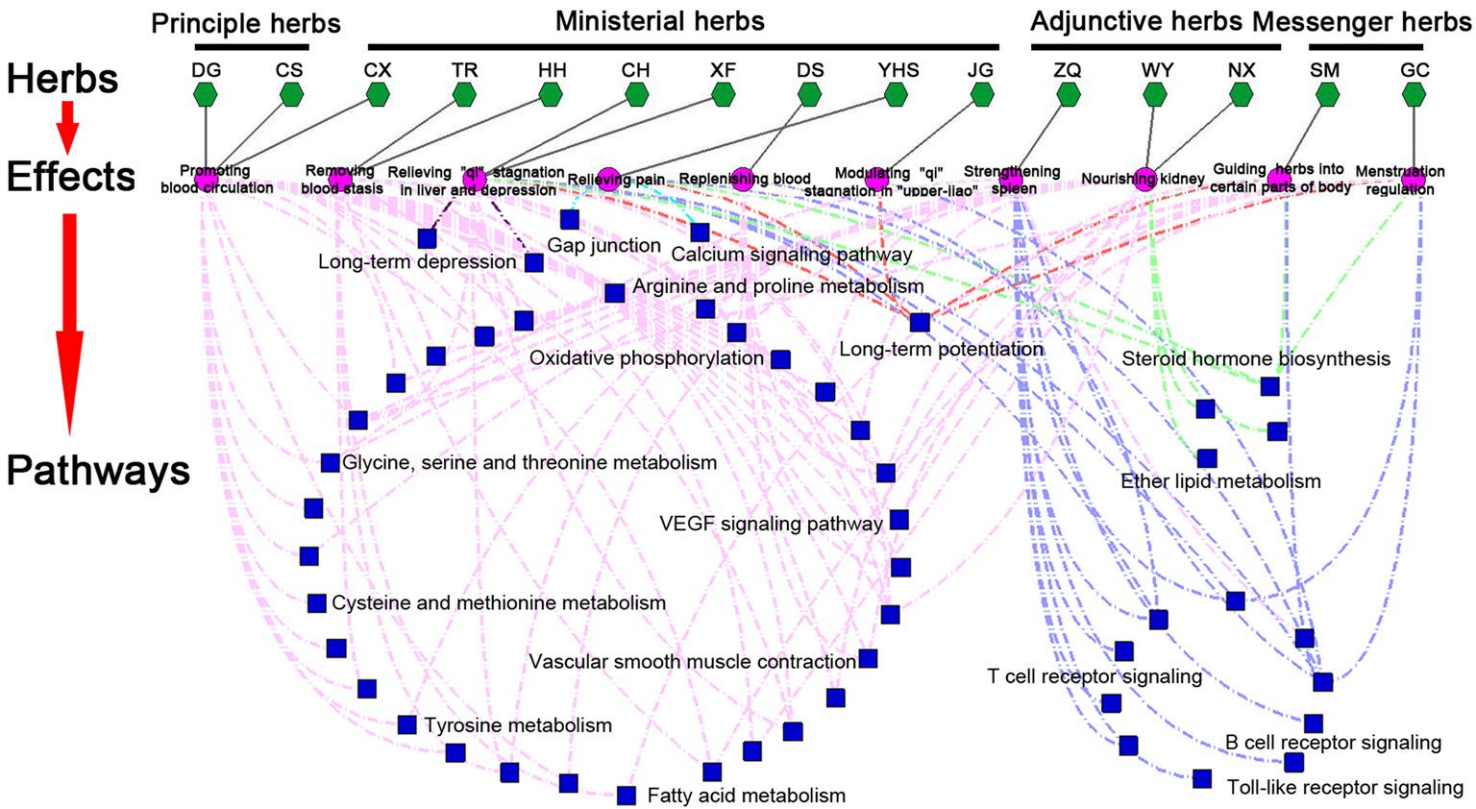
Illustration on the network of 15 herbs containing in QXHC-TCM pharmacology effects-pathways involved by their putative targets. *Green nodes* refer to 15 herbs in QXHC (DG*: Angelica sinensis*, Danggui; CS: *Radix Paeoniae Rubra,* Chishao; CX: *Ligusticum wallichii*, Chuanxiong; TR: seed of *Prunus persica(L.)Batsch*, Taoren; HH: *Carthamus tinctorius*, Honghua; CH: *Radix Bupleuri*, Chaihu; XF: *Cyperus rotundus*, Xiangfu; DS: *Salvia miltiorrhiza*, Danshen; YHS: *Rhizoma Corydalis*, Yanhusuo; JG: *Platycodon grandiflorum*, Jigen; ZQ: *Fructus Aurantii*, Zhiqiao; WY: *Lindera aggregate*, Wuyao; NX: *Achyranthes bidentata*, Niuxi; SM, *Rhizoma Cimicifugae,* Shenma; GC: *Glycyrrhiza uralensis,* Gancao); *Purple nodes* refer to the pharmacology effects of 15 herbs in QXHC from the perspective of TCM; *Blue nodes* refer to the corresponding pathways involved by QXHC putative targets

To get an initial sense of the biological processes and pathways enriched by QXHC putative targets, we performed the functional enrichment analysis based on GO and KEGG Pathway database. As shown in Fig. 2, the biological processes and pathways involved by QXHC putative targets are often associated with the main therapeutic effects of the corresponding herbs. Especially, the herbs DG and CS have been indicated to play a role in activating blood circulation and enriching blood during the progression of menstrual disorders [17]; Accordingly, the putative targets of the two herbs were significantly associated with amyotrophic lateral sclerosis pathway, cardiac muscle contraction and various nutrient metabolic pathways, such as alanine, aspartate and glutamate metabolism, arginine and proline metabolism,as well as Glycine, serine and threonine metabolism; Herbs DS, TR, HH and CX function as ministerial drugs in QXHC, and has been found to exert synergistic effects with herbs DG and CS mainly in removing blood stasis and regulating blood circulation [18–20], in line with which, our enrichment analysis revealed that the putative targets of these herbs were involved into several hemopoiesis-related pathways, including amyotrophic lateral sclerosis, vascular smooth muscle contraction and VEGF signaling pathways; In the TCM theory, the main biological functions of the liver in human body is to regulate the circulation of “*qi*”, and the stagnation of “*qi*” often leads to the occurrence of diseases and depression [54]; Interestingly, our pathway enrichment analysis revealed that the putative targets of herbs CH, XF, YHS and JG were significantly associated with various emotion regulation-related pathways, such as long-term potentiation, long-term depression and neuroactive ligand-receptor interaction; It has been indicated that the adjunctive drugs WY and NX, and the messenger drug GC assist with DG and CS in nourishing the kidney and strengthening spleen for the treatment of menstrual disorders [24–26]. Here, we found that the putative targets of herbs DG, WY, NX, ZQ and GC were significantly associated with the pathways involved in the absorption and metabolism of various nutrients, including alanine, aspartate and glutamate metabolism, arginine and proline metabolism and phenylalanine metabolism; Moreover, the putative targets of ZQ may frequently play a role in immune modulation-related pathways, such as T/B cell receptor signaling pathways.

### Pharmacological mechanisms of QXHC acting on menstrual disorders

From the above functional enrichment analysis, we were able to infer that QXHC could alleviate the pathological changes of menstrual disorders. Then, we asked which putative targets played crucial roles in the therapeutic effects of QXHC and how they interacted with each other. To address this problem, the interaction network using the links among QXHC putative targets and the known therapeutic targets of menstrual disorders (drug target-disease gene network, Additional file 2: Table S3) was constructed. This network consisted of 695 nodes and 4455 edges. A total of 362 hubs were identified since they had many connections with other nodes in the network. After that, the hub network was constructing using the direct interactions among hubs, and then, four topological features, including degree, betweenness, closeness and k-value, were calculated for each hub to screen the major hubs with topological importance. As a result, 89 major hubs were identified. Among them, 66 major hubs, which were QXHC putative targets, were considered as QXHC candidate targets in the treatment of menstrual disorders (Additional file 2: Table S4).

**Fig. 3:**
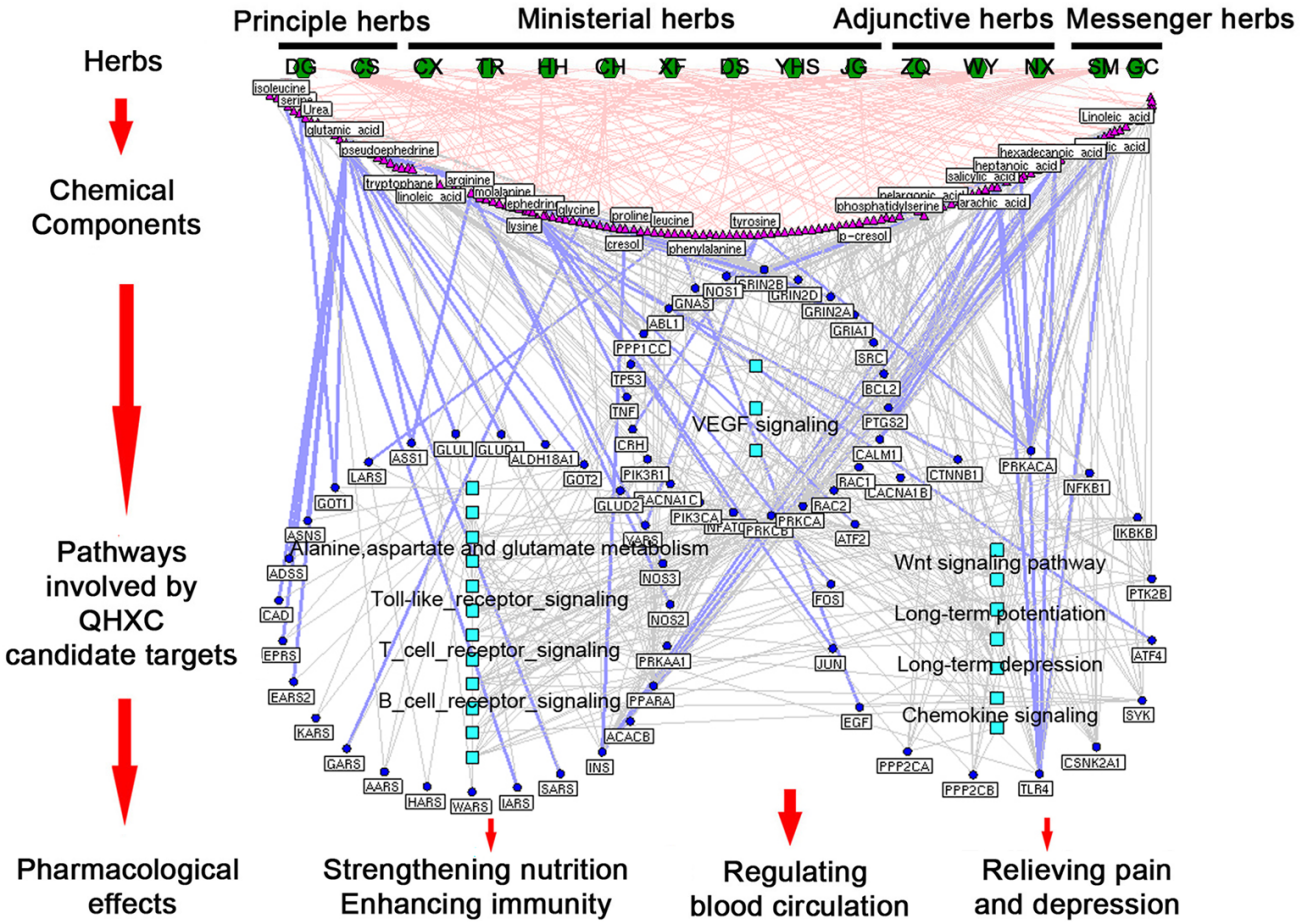
Network of herbs containing in QXHC, chemical components, QXHC candidate targets, the corresponding pathways and pharmacological effects. *Green nodes* refer to 15 herbs in QXHC (DG*: Angelica sinensis*, Danggui; CS: *Radix Paeoniae Rubra,* Chishao; CX: *Ligusticum wallichii*, Chuanxiong; TR: seed of *Prunus persica(L.)Batsch*, Taoren; HH: *Carthamus tinctorius*, Honghua; CH: *Radix Bupleuri*, Chaihu; XF: *Cyperus rotundus*, Xiangfu; DS: *Salvia miltiorrhiza*, Danshen; YHS: *Rhizoma Corydalis*, Yanhusuo; JG: *Platycodon grandiflorum*, Jigen; ZQ: *Fructus Aurantii*, Zhiqiao; WY: *Lindera aggregate*, Wuyao; NX: *Achyranthes bidentata*, Niuxi; SM, *Rhizoma Cimicifugae,* Shenma; GC: *Glycyrrhiza uralensis,* Gancao); *Blue round nodes* refer to candidate targets of 15 herbs in QXHC; *Purple line in bold* refer to the interactions of chemical components of QXHC with corresponding key targets

Moreover, further pathway enrichment analysis demonstrated that QXHC candidate targets were significantly associated with hemopoiesis-related pathways, such as VEGF signaling pathway and Vascular smooth muscle contraction; Analgesia-related pathways, such as MAPK signaling pathway, Wnt signaling pathway and Chemokine signaling pathway; Nutrient absorption and metabolism-related pathways, such as aminoacyl-tRNA biosynthesis, alanine, aspartate and glutamate metabolism, and insulin signaling pathway; Emotion regulation-related pathways, such as long-term potentiation, neurotrophin signaling pathway, long-term depression and gap junction; As well as immune modulation-related pathways, such as T/B cell receptor signaling pathway and Toll-like receptor signaling pathway. Figure 3 illustrated the relationship among herbs, chemical components, QXHC candidate targets and the associated pathways.

Among QXHC candidate targets, PRKCA (for DG, DS and CH), PTGS2 (for CS, CX, HH and TR), NOS3 (for DG and NX), SRC (for DG and NX) and PRKCB (for DS, CH, CX and ZQ) were involved into VEGF signaling pathway, which plays a crucial role in the formation of new blood vessels from existing vessels [55]. In addition, PRKCA (for DG, DS and CH), PRKACA (for CH, CX and HH), PPP1CC (for DS, DG and HH), CALM1 (for CH, CX, HH, TR and WY) and PRKCB (for DS, CH, CX and ZQ), all function as components in the pathway of vascular smooth muscle contraction. The smooth muscle cells directly affect the contraction of the vascular wall and thus modulate the alterations of blood vessel lumen. The changes in vascular smooth muscle contraction seriously influences blood pressure. On this basis, accumulating therapeutic strategies have been developed to be involved into the regulation of vascular smooth muscle cells during various pathological processes [56]. Generally, the normal menstrual cycle is featured by changes in radial artery distensibility in the ovulatory phase, which may be caused by the reduced estrogen in vascular smooth muscle tone. It has also been reported that vascular smooth muscle contraction may influence the arterial stiffening in the luteal phase due to a complex hormonal environment [57]. Therefore, QXHC may activate blood circulation and enrich blood by targeting its candidate targets that were components of VEGF signaling pathway and vascular smooth muscle contraction.

Moreover, dysmenorrhea is one of the most common menstrual disorders experienced by females, and is characterized by painful uterine cramps during the menses. It adversely affects the daily life and social performance of females [1]. Among QXHC candidate targets, PRKACA (for DG, DS and CH), TP53 (for GC), RAC2 (for YHS), JUN (for ZQ), RAC1 (for YHS) and PRKACA (for CH, CX, HH and TR) are involved into both MAPK and Wnt signaling pathways. MAPK signaling pathway plays important roles in the formation and transduction of neuropathic pain and numerous drugs in clinics produce analgesic effect through this signaling pathway [58]. It is also implicated in endometriosis pathogenesis. RAC1 is essential for the integrin-induced MAPK activation and its up-regulation contributes to the activation of MAPK pathway in patients with endometriosis [59]. The tumor suppressor TP53 is down-regulated throughout the menstrual cycle and might act as molecular targets for the diagnosis of endometriosis [60]. Wnt signaling pathway is involved into the development of nervous systems, and participants in the development of neuropathic pain after nerve injury and cancers [61]. Notably, Wnt signaling pathway has been proved to regulate the proliferation of menstrual blood derived stem cells by the trans-localization of activated-β-catenin protein [62]. It could also balance the estrogen-induced proliferation and progesterone-induced differentiation in the duration of menses [63]. These findings suggest that analgesic effect may be one of the main pharmacological actions of QXHC.

Accumulating studies have indicated that vegan diets may be a cause of menstrual disorders and thus, the absorption and metabolism of nutrients is indispensible in maintaining normal menstrual cycles [64]. According to our data, several QXHC candidate targets, such as INS (for CH, CX, DG and HH), PRKACA (for CH, CX and HH), PPP1CC (for JG, GC, NX and SM) and CALM1 (for CH, CX, HH, TR and WY), were all significantly associated with Insulin signaling pathway, which may be implicated into many biological processes, including lipid synthesis and storage, protein and glycogen synthesis, as well as cell growth and survival [65]. The defects in insulin signaling pathway often lead to insulin resistance, which is an important risk factor for metabolic dysfunctions and other diseases [66]. Hence, our data here indicated that QXHC candidate targets were significantly associated with nutrients-related pathways for the normal absorption and metabolism of nutrients.

From the perspective of TCM, the function of the liver system is closely related to emotion regulation. Chinese herbs that can act on the liver to regulate *“qi”* circulation may efficiently improve the patients’ emotional states in clinics [67]. The spleen belongs to a primary hematopoietic and peripheral lymphoid organ that is implicated in breakdown of aged erythrocytes, and T/B-like cells for antigen capturing, antigen presentation, as well as initiation of the adaptive immune response [68]. Nourishing spleen can improve patients’ immune indexes and enhance their immune function [69]. QXHC candidate targets, such as PRKCA (for DG, DS, CH, CH and ZQ), GRIN2B (for HH, TR, CS, DG and NX), ATF4 (for ZQ), GRIN2D (shared by 15 herbs), GRIN2A (for CH, HH, TR, CS and DG), GRIA1 (for DG and NX), PPP1CC (for DG, GC, NX and CX) and CALM1 (for CH, CX, HH, TR and WY) were enriched in emotional regulation pathways. Long-term depression and long-term potentiation are persistent modifications of the synaptic strength that were induced by different rises of intracellular calcium ion concentration respectively [70]. In the neostriatum, long-term depression may exert synaptic efficiency by storing motor skills within the basal ganglia, and this synaptic plasticity is regarded to be correlated with some cognitive and emotional activities, which is modulated by cortical-striatal-pallidal-thalamic loops. Striatal long-term potentiation has been considered as a potential therapeutic target for various mood disorders [71, 72]. ATF4 is one member of ATF/cAMP response element binding protein family that can negatively regulate synaptic plastic and memory. Its knockdown causes profound impairment in the induction of two forms of synaptic plasticity long-term depression and long-term potentiation [73]. The mRNA expression of GRIA1 related to these two pathways were significantly decreased at the cerebellar postsynaptic density of mouse cerebellum exposed to arsenic, which is a neurotoxin that induces dysfunction of learning and memory [74].

### Binding efficiency of QXHC candidate targets with the corresponding chemical components containing in QXHC

Molecular docking is one of the most frequently used structure-based drug design method and has wide range of applications in molecular recognition event analysis, such as binding energies and molecular interactions [75]. LibDock is a powerful high-throughput docking program that is on the strength of the algorithm developed by Diller and Merz to guide the molecular docking using protein binding site features. LibDock scoring could execute predictions of binding energy on the basis of the binding efficiency of ligand-receptor complexes with a high speed and a substantial degree of accuracy [76]. In the current study, a docking score is an indication of the binding efficiency between the chemical components containing in QXHC and the corresponding candidate targets for the treatment of menstrual disorders molecular targets. As listed in Additional file 2: Table S5, a total of 41 pairs of QXHC candidate targets (n=24) and the corresponding chemical components (n=21) were delivered into docking. As shown in Table 1, 20 pairs of chemical component-QXHC candidate target interactions had strong binding free energy, since their docking scores were higher than the median value of all pairs.

**Table 1:** Docking scores of QXHC candidate targets with the corresponding chemical components containing in QXHC

Herbs	Chemical_components	Major_targets	Libdockscores
CH	hexadecanoic_acid	ABL1	109.602
CH	hexadecanoic_acid	PRKACA	104.17
CH	hexadecanoic_acid	TLR4	100.788
CX	Linoleic_acid	PTGS2	117.673
CX	arachic_acid	ABL1	119.662
CX	arachic_acid	PRKACA	119.296
CX	arachic_acid	TLR4	112.936
DG	arginine	ASS1	107.341
DG	arginine	NOS2	106.253
DG	phosphatidylserine	PRKCA	119.477
DG	tryptophane	WARS	112.00
HH	arachic_acid	ABL1	119.662
HH	arachic_acid	PRKACA	119.296
HH	arachic_acid	TLR4	112.936
HH	linoleic_acid	PTGS2	117.673
NX	arginine	ASS1	107.341
NX	arginine	NOS2	106.253
NX	tryptophane	WARS	112
TR	linoleic_acid	PTGS2	117.673
TR	tryptophane	WARS	112.00

## Discussion

Menstrual disorders are a group of female diseases with a high prevalence [1]. Short- or long-term uncomfortable feelings on females seriously impair their life quality. Increasing evidence suggests that multi-component therapeutics, which is characterized by the simultaneous actions of two or more agents on multiple targets, may be efficient in controlling complex diseases. Of note, Chinese herbal formulae are considered to be an empirical system of multi-components and multi-targets, and specialize in treating diseases in an integrative manner. The rapid development of network pharmacology-based methods provide new possibilities to identify active multi-components and their interactions with the corresponding targets and diseases. In the current study, we combined drug target prediction, network analysis and target validation to reveal the associations among the chemical components of each herb containing in QXHC, their candidate targets and menstrual disorders-related pathways. Our main findings are summarized as follows:

1. A total of 1022 putative targets of 15 herbs in QXHC were predicted, and gave us a first glance at the investigation of the pharmacological mechanisms of QXHC acting on menstrual disorders;
2. QXHC candidate targets in the treatment of menstrual disorders are significantly associated with several biological pathways, such as VEGF and Chemokine signaling pathways, Alanine, aspartate and glutamate metabolism, Long-term depression and T/B cell receptor signaling pathway, which are involved into the major pathological processes of menstrual disorders, including abnormal menstrual bleeding, dysmenorrhea, malnutrition, emotional disturbances, as well as immune dysregulation, respectively.
3. Further molecular docking simulation confirmed that 20 pairs of QXHC candidate targets and the corresponding chemical components had the strong binding free energy.

In conclusion, the current study provides a novel and scientific approach to holistically decipher that the pharmacological mechanisms of QXHC in the treatment of menstrual disorders may be associated with its involvement into hemopoiesis, analgesia, nutrients absorption and metabolism, mood regulation, as well as immune modulation. However, this pilot study was performed on the basis of data analysis, and further experimental experiments were demanded to validate these hypotheses.

## Additional files

**Additional file 1.** Minimum Standards of Reporting Checklist.

**Additional file 2.** Additional Tables. **Table S2** The putative targets for 311 chemical components in QXHC.

## Abbreviations

QXHC: QiXueHe Capsule
TCM: Traditional Chinese medicine
DG: *Angelica sinensis*
CS: *Radix Paeoniae Rubra*
CX: *Ligusticum wallichii*
TR: seed of *Prunus persica*(L.)*Batsch*
HH: *Carthamus tinctorius*
CH: *Radix Bupleuri*
XF: *Cyperus rotundus*
DS: *Salvia miltiorrhiza*
YHS: *Rhizoma Corydalis*
JG: *Platycodon grandiflorum*
ZQ: *Fructus Aurantii*
WY: *Lindera aggregate*
NX: *Achyranthes bidentata*
SM: *Rhizoma Cimicifugae*
GC: *Glycyrrhiza uralensis*
FDA: Food and Drug Administration, USA
PPI data: protein–protein interaction data
HAPPI: human annotated and predicted protein interaction database
OPHID: online predicted human interaction database
HPRD: human protein reference database
MINT: molecular interaction database
DIP: database of interacting proteins
GO: gene ontology
DAVID: database for annotation, visualization and integrated discovery
KEGG: kyoto encyclopedia of genes and genomes
ALS: amyotrophic lateral sclerosis
