# Supplementary material for: A network pharmacology-based strategy deciphers the underlying molecular mechanisms of Qixuehe Capsule in the treatment of menstrual disorders
Source: Chin Med. 2017 Aug 21;12:23. doi: 10.1186/s13020-017-0145-x (PMC5563918; doi:10.1186/s13020-017-0145-x)
Supplement: Supplementary file 2 — Additional file 2. Additional Tables. Table S2 The putative targets for 311 chemical components in QXHC. [file 13020_2017_145_MOESM2_ESM.pdf]

Tabel S1 Detailed information about the known therapeutic targets for the treatment of menstrual disorders

Drugbankid	UniProt_Accession number	Uniprot_ID	Gene_Symbol
DB00367	P03372	ESR1_HUMAN	ESR1
DB00367	P06401	PRGR_HUMAN	PGR
DB00367	P10275	ANDR_HUMAN	AR
DB00367	P18405	S5A1_HUMAN	SRD5A1
DB00482	O15530	PDPK1_HUMAN	PDPK1
DB00482	P35354	PGH2_HUMAN	PTGS2
DB00533	P15502	ELN_HUMAN	ELN
DB00533	P35354	PGH2_HUMAN	PTGS2
DB00554	P23219	PGH1_HUMAN	PTGS1
DB00554	P35354	PGH2_HUMAN	PTGS2
DB00575	P08913	ADA2A_HUMAN	ADRA2A
DB00575	P18089	ADA2B_HUMAN	ADRA2B
DB00575	P18825	ADA2C_HUMAN	ADRA2C
DB00575	P25100	ADA1D_HUMAN	ADRA1D
DB00575	P35348	ADA1A_HUMAN	ADRA1A
DB00575	P35368	ADA1B_HUMAN	ADRA1B
DB00712	P23219	PGH1_HUMAN	PTGS1
DB00712	P35354	PGH2_HUMAN	PTGS2
DB00784	P23219	PGH1_HUMAN	PTGS1
DB00784	P35354	PGH2_HUMAN	PTGS2
DB00788	P23219	PGH1_HUMAN	PTGS1
DB00788	P35354	PGH2_HUMAN	PTGS2
DB00814	P23219	PGH1_HUMAN	PTGS1
DB00814	P35354	PGH2_HUMAN	PTGS2
DB00939	O43525	KCNQ3_HUMAN	KCNQ3
DB00939	O43526	KCNQ2_HUMAN	KCNQ2
DB00939	P09917	LOX5_HUMAN	ALOX5
DB00939	P23219	PGH1_HUMAN	PTGS1
DB00939	P35354	PGH2_HUMAN	PTGS2
DB01009	P23219	PGH1_HUMAN	PTGS1
DB01009	P25024	CXCR1_HUMAN	CXCR1
DB01009	P35354	PGH2_HUMAN	PTGS2
DB01050	P00750	TPA_HUMAN	PLAT
DB01050	P07204	TRBM_HUMAN	THBD
DB01050	P10415	BCL2_HUMAN	BCL2
DB01050	P12104	FABPI_HUMAN	FABP2
DB01050	P13569	CFTR_HUMAN	CFTR
DB01050	P23219	PGH1_HUMAN	PTGS1
DB01050	P35354	PGH2_HUMAN	PTGS2
DB01050	P37231	PPARG_HUMAN	PPARG
DB01200	P08908	5HT1A_HUMAN	HTR1A
DB01200	P08913	ADA2A_HUMAN	ADRA2A
DB01200	P14416	DRD2_HUMAN	DRD2
DB01200	P18089	ADA2B_HUMAN	ADRA2B
DB01200	P18825	ADA2C_HUMAN	ADRA2C
DB01200	P21728	DRD1_HUMAN	DRD1
DB01200	P21917	DRD4_HUMAN	DRD4
DB01200	P21918	DRD5_HUMAN	DRD5
DB01200	P25100	ADA1D_HUMAN	ADRA1D
DB01200	P28221	5HT1D_HUMAN	HTR1D
DB01200	P28222	5HT1B_HUMAN	HTR1B
DB01200	P28223	5HT2A_HUMAN	HTR2A
DB01200	P28335	5HT2C_HUMAN	HTR2C
DB01200	P34969	5HT7R_HUMAN	HTR7
DB01200	P35348	ADA1A_HUMAN	ADRA1A
DB01200	P35368	ADA1B_HUMAN	ADRA1B
DB01200	P35462	DRD3_HUMAN	DRD3
DB01200	P41595	5HT2B_HUMAN	HTR2B
DB05749	P11511	CP19A_HUMAN	CYP19A1

Table S2 List of putative targets for each herb containing Qixuehe Capsule

Herbname (abbraviation)	Genesymbol
CH	ABAT
CH	ABL1
CH	ACADSB
CH	ACOT13
CH	ACOX1
CH	ACSL3
CH	ACSL4
CH	ADH1B
CH	AKR1C1
CH	AKR1C2
CH	AKR1C3
CH	AKR1D1
CH	ALDH2
CH	ALDH5A1
CH	ANXA1
CH	AR
CH	ARF1
CH	ARF6
CH	ATP1A1
CH	ATP2A1
CH	ATP5A1
CH	ATP5B
CH	ATP5C1
CH	ATP6V1C1
CH	B3GAT1
CH	BCHE
CH	BCL2
CH	CA1
CH	CA12
CH	CA14
CH	CA2
CH	CA3
CH	CA4
CH	CA5A
CH	CA5B
CH	CA6
CH	CA7
CH	CA9
CH	CALM1
CH	CDK6
CH	CFTR
CH	COMT
CH	CSNK2A1
CH	CYP17A1
CH	CYP19A1
CH	CYP1A2
CH	CYP27B1
CH	CYP2A6
CH	CYP2C8

CH	DBI
CH	DDX6
CH	E
CH	ECI2
CH	ELOVL4
CH	ESR1
CH	ESR2
CH	FABP2
CH	FADS1
CH	FADS2
CH	FFAR1
CH	FKBP1A
CH	FURIN
CH	GABRA1
CH	GABRA2
CH	GABRA3
CH	GABRA4
CH	GABRA5
CH	GABRA6
CH	GABRB1
CH	GABRB2
CH	GABRB3
CH	GABRD
CH	GABRE
CH	GABRG1
CH	GABRG2
CH	GABRG3
CH	GABRP
CH	GABRQ
CH	GLRA3
CH	GLTP
CH	GM2A
CH	GRIN1
CH	GRIN2A
CH	GRIN2B
CH	GRIN2C
CH	GRIN2D
CH	GRIN3A
CH	GRIN3B
CH	GSTP1
CH	GUCA1A
CH	HA
CH	HAO1
CH	HCK
CH	HDAC2
CH	HDAC9
CH	HIBCH
CH	HIF1AN
CH	HMGCR
CH	HNF4A
CH	HNF4G

CH	HPGDS
CH	HSD11B1
CH	HSD17B1
CH	HSD17B11
CH	HSD3B1
CH	IGHG1
CH	IGHG2
CH	INS
CH	ISYNA1
CH	ITGB2
CH	ITPR1
CH	LALBA
CH	LIP3
CH	LSS
CH	LY96
CH	MAOB
CH	MT1417
CH	MTTP
CH	NAGA
CH	NCOA1
CH	NCOA2
CH	NR1I2
CH	NR1I3
CH	NR3C1
CH	72
CH	NR3C2
CH	OGDH
CH	OPRK1
CH	P2RY12
CH	PAEP
CH	PAPSS1
CH	PGR
CH	PIK3CG
CH	PIM1
CH	PKIA
CH	PLA2G1B
CH	PLA2G2A
CH	PLA2G2D
CH	PLA2G2E
CH	PLAT
CH	PMP2
CH	PPARA
CH	PPARD
CH	PPARG
CH	PPP1CC
CH	PPP3CA
CH	PPP3R1
CH	PPT1
CH	PRKACA
CH	PRKCA
CH	PRKCB

CH	PRLR
CH	PTGIR
CH	PTGIS
CH	PTGS1
CH	PTGS2
CH	PTK2B
CH	PVR
CH	RCVRN
CH	RHO
CH	RORA
CH	RV0233
CH	S100B
CH	SCN10A
CH	SCN11A
CH	SCN1A
CH	SCN1B
CH	SCN2A
CH	SCN2B
CH	SCN3A
CH	SCN3B
CH	SCN4A
CH	SCN4B
CH	SCN5A
CH	SCN7A
CH	SCN8A
CH	SCN9A
CH	SEC14L2
CH	SERPINA1
CH	SIGMAR1
CH	SLC8A1
CH	SOAT1
CH	SOAT2
CH	SP_1951
CH	SQLE
CH	SRD5A1
CH	STK17B
CH	SULT2A1
CH	SULT2B1
CH	SYK
CH	TGFBR2
CH	THBD
CH	TLR4
CH	TOP2A
CH	TRAPPC3
CH	TRDMT1
CH	TREM1
CH	TRPA1
CH	TRPM8
CH	TRPV1
CH	TRPV3
CH	UGT3A1

CH	VDR
CH	YWHAE
CH	ARGG
CH	BFR
CH	BGLA
CH	CAMC
CH	CC4
CH	CCRA
CH	CELCCF
CH	CELCCG
CH	CELH
CH	CHOB
CH	COBT
CH	CUMD
CH	CYP102A1
CH	CYPC
CH	DCTD
CH	DLGD
CH	ENO
CH	ERYF
CH	FABB
CH	FABG3
CH	FABZ
CH	FADR
CH	FHUA
CH	FUCI
CH	GAG
CH	GAG-POL
CH	GLCB
CH	GLPF
CH	HISD
CH	KSI
CH	LIPB
CH	LPXA
CH	LPXC
CH	LUXF
CH	MALE
CH	MDLB
CH	METE
CH	MEXA
CH	MUTL
CH	MUTY
CH	NAGJ
CH	NCSA
CH	NEF
CH	NPRS
CH	NRDB
CH	ONR
CH	PAB
CH	PFLB
CH	PHNA

CH	RHAA
CH	RMLC
CH	SERA
CH	TCP14
CH	TESA
CH	TONB
CH	TTGR
CH	XYLA
CS	ABO
CS	AKR1C1
CS	AKR1C2
CS	AKR1C3
CS	AR
CS	ATP1A1
CS	ATP5A1
CS	ATP5B
CS	ATP5C1
CS	CA2
CS	CTRB1
CS	CTSB
CS	DAO
CS	EDNRA
CS	ESR1
CS	ESR2
CS	GABRA1
CS	GABRA2
CS	GABRA3
CS	GABRA4
CS	GABRA5
CS	GABRA6
CS	GABRB1
CS	GABRB2
CS	GABRB3
CS	GABRD
CS	GABRE
CS	GABRG1
CS	GABRG2
CS	GABRG3
CS	GABRP
CS	GABRQ
CS	GRIN1
CS	GRIN2A
CS	GRIN2B
CS	GRIN2C
CS	GRIN2D
CS	GRIN3A
CS	GRIN3B
CS	HRSP12
CS	HSD17B1
CS	LCN2
CS	LIP3

CS	LSS
CS	MIF
CS	NCOA2
CS	NR1I2
CS	NR1I3
CS	NR3C1
CS	PGR
CS	PPARA
CS	PRDX5
CS	PTGS1
CS	PTGS2
CS	RAB9A
CS	RORA
CS	SIGMAR1
CS	SULT2A1
CS	SULT2B1
CS	VDR
CS	CHOB
CS	CHQB
CS	CLCA
CS	COCE
CS	CPO
CS	CUMD
CS	CUTA
CS	DHBE
CS	ELTB
CS	HUTH
CS	KSI
CS	MALT
CS	NFSB
CS	OXYR
CS	POBA
CS	PPTA
CS	PYP
CS	REPA
CX	72
CX	9
CX	ABAT
CX	ABL1
CX	ABO
CX	ACADSB
CX	ACHE
CX	ACOX1
CX	ACSL3
CX	ACSL4
CX	ACTA1
CX	AKR1C1
CX	AKR1C2
CX	AKR1C3
CX	ALDH2
CX	ALDH5A1

CX	AMY1A
CX	AMY2A
CX	AMY2B
CX	AOX1
CX	AR
CX	ARF1
CX	ARF6
CX	ATOX1
CX	ATP1A1
CX	ATP5A1
CX	ATP5B
CX	ATP5C1
CX	BCHE
CX	CA1
CX	CA12
CX	CA14
CX	CA2
CX	CA3
CX	CA4
CX	CA5A
CX	CA5B
CX	CA6
CX	CA7
CX	CA9
CX	CACNA1A
CX	CACNA1B
CX	CACNA1C
CX	CACNA1D
CX	CACNA1F
CX	CACNA1G
CX	CACNA1H
CX	CACNA1I
CX	CACNA1S
CX	CACNA2D1
CX	CACNA2D2
CX	CACNA2D3
CX	CACNB1
CX	CACNB2
CX	CACNB3
CX	CACNB4
CX	CACNG1
CX	CALM1
CX	CDA
CX	CDK6
CX	COMT
CX	CSNK2A1
CX	CTRB1
CX	CYP11B2
CX	CYP19A1
CX	CYP27B1
CX	CYP2B6

CX	CYP2C8
CX	DHODH
CX	DPYD
CX	ECI2
CX	ELOVL4
CX	ESR1
CX	ESR2
CX	FADS1
CX	FADS2
CX	FFAR1
CX	FHIT
CX	FKBP1A
CX	FURIN
CX	GABRA1
CX	GABRA2
CX	GABRA3
CX	GABRA4
CX	GABRA5
CX	GABRA6
CX	GABRB1
CX	GABRB2
CX	GABRB3
CX	GABRD
CX	GABRE
CX	GABRG1
CX	GABRG2
CX	GABRG3
CX	GABRP
CX	GABRQ
CX	GCK
CX	GLRA3
CX	GLT6D1
CX	GLTP
CX	GM2A
CX	GNPDA1
CX	GRIN1
CX	GRIN2A
CX	GRIN2B
CX	GRIN2C
CX	GRIN2D
CX	GRIN3A
CX	GRIN3B
CX	GUCA1A
CX	HAO1
CX	HCK
CX	HDAC2
CX	HDAC9
CX	HIBCH
CX	HK1
CX	HNF4A
CX	HNF4G

CX	HSD11B1
CX	HSD17B1
CX	IFNB1
CX	IGHG1
CX	INS
CX	KRTAP5-2
CX	KRTAP5-3
CX	LALBA
CX	LCN2
CX	LCTL
CX	LGALS2
CX	LGALS7
CX	LIP3
CX	LSS
CX	LY96
CX	LYZ
CX	MB
CX	MED1
CX	MIF
CX	MT1417
CX	MTTP
CX	NCAN
CX	NCOA1
CX	NCOA2
CX	NR1I2
CX	NR1I3
CX	NR3C1
CX	NR3C2
CX	NUDT9
CX	OGDH
CX	P2RY12
CX	PAEP
CX	PCYT1A
CX	PCYT1B
CX	PGR
CX	PHOSPHO1
CX	PIK3CG
CX	PIM1
CX	PKIA
CX	PLA2G1B
CX	PLA2G2A
CX	PLA2G2D
CX	PLA2G2E
CX	PLD1
CX	PLD2
CX	PMP2
CX	PPARA
CX	PPARD
CX	PPARG
CX	PPP1CC
CX	PPP3CA

CX	PPP3R1
CX	PPT1
CX	PRKACA
CX	PRKCA
CX	PRKCB
CX	PRLR
CX	PTGIR
CX	PTGIS
CX	PTGS1
CX	PTGS2
CX	PTK2B
CX	PVR
CX	PYGL
CX	PYGM
CX	RCVRN
CX	RHO
CX	RORA
CX	RTCB
CX	RV0233
CX	S100B
CX	SCN10A
CX	SCN11A
CX	SCN1A
CX	SCN1B
CX	SCN2A
CX	SCN2B
CX	SCN3A
CX	SCN3B
CX	SCN4A
CX	SCN4B
CX	SCN5A
CX	SCN7A
CX	SCN8A
CX	SCN9A
CX	SEC14L2
CX	SFTPD
CX	SHBG
CX	SIGLEC1
CX	SIGMAR1
CX	SLC8A1
CX	SMARCA5
CX	SOAT1
CX	SOAT2
CX	SQLE
CX	SRD5A1
CX	SRD5A2
CX	SRD5A3
CX	STK17B
CX	SULT2A1
CX	SULT2B1
CX	SYK

CX	TLR4
CX	TOP2A
CX	TRAPPC3
CX	TRPV1
CX	TYMS
CX	UCKL1
CX	UGT3A1
CX	VDR
CX	XDH
CX	YWHAE
CX	ABFA
CX	ACTIII
CX	AGLA
CX	ALGD
CX	ALSB
CX	AMPC
CX	AMS
CX	AMY
CX	AMYA
CX	AMYE
CX	AMYM
CX	AMYP
CX	ARAC
CX	ARAF
CX	ASPA
CX	BGL3
CX	BLA
CX	BLACTX-M-14
CX	BLACTX-M-27
CX	BLACTX-M-9A
CX	BOTB
CX	BPHC
CX	BUDC
CX	CATA
CX	CBHA
CX	CEL5A
CX	CELA
CX	CELA1
CX	CELB
CX	CELC307
CX	CELCCF
CX	CELCCG
CX	CELD
CX	CELS
CX	CENC
CX	CEX
CX	CFT
CX	CGT
CX	CHOB
CX	CSLB
CX	CTXB

CX	CUMD
CX	CYP102A1
CX	CYPC
CX	DEOA
CX	DHBE
CX	DMSA
CX	EBGA
CX	ECO
CX	ENGF
CX	ENTB
CX	FABB
CX	FABI
CX	FABZ
CX	FADR
CX	FHUA
CX	GAG
CX	GAG-POL
CX	GALM
CX	GANB
CX	GDHB
CX	GFO
CX	GLK
CX	HMUO
CX	HUTH
CX	KSI
CX	LACZ
CX	LAMB
CX	LECB
CX	LGTC
CX	LIPB
CX	LPXC
CX	LUXF
CX	MAF
CX	MALE
CX	MALP
CX	MDLB
CX	MGLB
CX	MNDD
CX	MOXC
CX	MUTY
CX	NAHD
CX	NEDA
CX	NEF
CX	NOS
CX	NPLT
CX	PAB
CX	PAC
CX	PCAG
CX	PCAH
CX	PDP
CX	PHR

CX	POBA
CX	PPGMK
CX	PYP
CX	PYRC
CX	PYRD
CX	PYRDA
CX	PYRE
CX	RBSK
CX	RSUA
CX	SACB
CX	SCRY
CX	SPOII
CX	STX2EB
CX	STXB
CX	TCP14
CX	TESA
CX	TETX
CX	TONB
CX	TREZ
CX	TTGR
CX	TVAI
CX	TVAII
CX	UBIC
CX	UDG
CX	UNG
CX	XLNA
CX	XYLA
CX	XYNA
CX	XYNY
CX	XYNZ
DG	9
DG	AADAT
DG	AARS
DG	AARS2
DG	AASS
DG	ABAT
DG	ABO
DG	ACACB
DG	ACADSB
DG	ACCS
DG	ACO2
DG	ACOT13
DG	ACP1
DG	ACTA1
DG	ACY1
DG	ACY3
DG	ADH1B
DG	ADSS
DG	ADSSL1
DG	AGXT
DG	AGXT2

DG	AKR1B1
DG	AKR1C1
DG	AKR1C2
DG	AKR1D1
DG	ALAD
DG	ALAS1
DG	ALAS2
DG	ALDH18A1
DG	ALDH5A1
DG	AMY1A
DG	AMY2A
DG	AMY2B
DG	ANXA5
DG	AOX1
DG	APRT
DG	AR
DG	ARF1
DG	ARG1
DG	ARG2
DG	ASL
DG	ASNS
DG	ASPA
DG	ASPH
DG	ASRGL1
DG	ASS
DG	ASS1
DG	ATOX1
DG	ATP1A1
DG	ATP2A1
DG	ATP6V1C1
DG	ATP8A1
DG	AZIN2
DG	B2M
DG	B3GAT1
DG	BAAT
DG	BBOX1
DG	BCAT1
DG	BCAT2
DG	BGLAP
DG	BHMT
DG	BHMT2
DG	BPI
DG	CAD
DG	CAMP
DG	CARS
DG	CARS2
DG	CBS
DG	CCBL1
DG	CCBL2
DG	CDO-1
DG	CDO1

DG	CKM
DG	CLPP
DG	COMT
DG	COX4I1
DG	COX5A
DG	COX5B
DG	COX6A2
DG	COX6B1
DG	COX6C
DG	COX7A1
DG	COX7B
DG	COX7C
DG	COX8A
DG	CPA1
DG	CPQ
DG	CRH
DG	CSAD
DG	CTH
DG	CTNS
DG	CTPS1
DG	CTRB1
DG	CTSB
DG	CTSD
DG	CXCL10
DG	CYP2B6
DG	CYTH2
DG	DAO
DG	DARS
DG	DARS2
DG	DDX6
DG	DGKD
DG	DGKG
DG	DHODH
DG	DNPEP
DG	DPP4
DG	DPYD
DG	DRD1
DG	DRD2
DG	DRD3
DG	DRD4
DG	DRD5
DG	E
DG	EARS2
DG	EGF
DG	ENOPH1
DG	ENPEP
DG	EPOR
DG	EPRS
DG	ESR1
DG	ESR2
DG	F10

DG	F2
DG	FAAH
DG	FARS2
DG	FARSA
DG	FARSB
DG	FHIT
DG	FOLH1
DG	FPGS
DG	FTCD
DG	GABRA1
DG	GABRA2
DG	GABRA3
DG	GABRA4
DG	GABRA5
DG	GABRA6
DG	GABRB1
DG	GABRB2
DG	GABRB3
DG	GABRD
DG	GABRE
DG	GABRG1
DG	GABRG2
DG	GABRG3
DG	GABRP
DG	GABRQ
DG	GAD1
DG	GAD2
DG	GAMT
DG	GARS
DG	GATB
DG	GATM
DG	GCAT
DG	GCK
DG	GCLC
DG	GCLM
DG	GCSH
DG	GGCX
DG	GIG18
DG	GLDC
DG	GLRA1
DG	GLRA2
DG	GLRA3
DG	GLRB
DG	GLS
DG	GLS2
DG	GLT6D1
DG	GLTP
DG	GLUD1
DG	GLUD2
DG	GLUL
DG	GLYAT

DG	GLYATL1
DG	GLYATL2
DG	GM2A
DG	GMPS
DG	GNMT
DG	GNPDA1
DG	GOT1
DG	GOT2
DG	GPI
DG	GPR18
DG	GPT
DG	GPT2
DG	GRIA1
DG	GRIA2
DG	GRIA3
DG	GRIA4
DG	GRID1
DG	GRID2
DG	GRIK1
DG	GRIK2
DG	GRIK3
DG	GRIK4
DG	GRIK5
DG	GRIN1
DG	GRIN2A
DG	GRIN2B
DG	GRIN2C
DG	GRIN2D
DG	GRIN3A
DG	GRIN3B
DG	GRM1
DG	GRM4
DG	GRM7
DG	GRM8
DG	GSS
DG	GSTM1
DG	GSTP1
DG	HA
DG	HAL
DG	HARS
DG	HBA1
DG	HBB
DG	HCAR2
DG	HCAR3
DG	HDC
DG	HIF1AN
DG	HK1
DG	HLA-B
DG	HNF1A
DG	HPGDS
DG	HSD17B1

DG	HSD17B6
DG	IARS
DG	IARS2
DG	IFNB1
DG	IGF2R
DG	IGHG1
DG	IL4I1
DG	INS
DG	ISYNA1
DG	ITPR1
DG	IYD
DG	KARS
DG	KRTAP5-2
DG	KRTAP5-3
DG	KYNU
DG	L3HYPDH
DG	LARS
DG	LARS2
DG	LCMT1
DG	LCMT2
DG	LCTL
DG	LGALS2
DG	LGALS7
DG	LGSN
DG	LIP3
DG	LSS
DG	LYZ
DG	M6PR
DG	MB
DG	ME2
DG	METAP2
DG	MGMT
DG	MIF
DG	MT-CO1
DG	MT-CO2
DG	MT-CO3
DG	MT-ND1
DG	MTAP
DG	MTR
DG	MTRR
DG	MTTP
DG	NADSYN1
DG	NAGA
DG	NAGS
DG	NCAN
DG	NCOA2
DG	NFS1
DG	NME1
DG	NNMT
DG	NOS1
DG	NOS2

DG	NOS3
DG	NPY
DG	NR1I2
DG	NR1I3
DG	NR3C1
DG	NR5A1
DG	NUDT9
DG	OAT
DG	OAZ1
DG	OAZ2
DG	OAZ3
DG	ODC1
DG	OPLAH
DG	OTC
DG	OXCT1
DG	OXCT2
DG	P2RY12
DG	P3H1
DG	P3H2
DG	P3H3
DG	P4HA1
DG	P4HA2
DG	P5CR2
DG	PAEP
DG	PAH
DG	PAICS
DG	PAPSS1
DG	PARS2
DG	PCCB
DG	PCTP
DG	PECR
DG	PFAS
DG	PGM1
DG	PGR
DG	PHYKPL
DG	PIM1
DG	PIN1
DG	PIPOX
DG	PISD
DG	PITPNA
DG	PKIA
DG	PLA2G1B
DG	PLA2G2A
DG	PLA2G2E
DG	PLOD1
DG	PLOD3
DG	PON1
DG	PPARA
DG	PPAT
DG	PPIA
DG	PPIB

DG	PPIC
DG	PPIF
DG	PPIG
DG	PPIH
DG	PPP1CC
DG	PRKACA
DG	PRKCA
DG	PROCR
DG	PRODH
DG	PROSC
DG	PRSS1
DG	PSAP
DG	PSAT1
DG	PTDSS1
DG	PTDSS2
DG	PTGIR
DG	PTGIS
DG	PTGS1
DG	PTGS2
DG	PTH
DG	PTPN1
DG	PYCR1
DG	PYCR2
DG	PYCRL
DG	PYGL
DG	PYGM
DG	QPRT
DG	RABGGTA
DG	RABGGTB
DG	RHO
DG	RNASE1
DG	RORA
DG	RTCB
DG	S100B
DG	S100G
DG	SARS
DG	SCARB1
DG	SCN2A
DG	SCN4A
DG	SDHA
DG	SDHB
DG	SDHC
DG	SDHD
DG	SDS
DG	SERPINB3
DG	SERPINC1
DG	SFTPD
DG	SHMT1
DG	SHMT2
DG	SIGLEC1
DG	SIGMAR1

DG	SLC13A1
DG	SLC13A2
DG	SLC13A3
DG	SLC19A3
DG	SLC1A1
DG	SLC1A2
DG	SLC1A3
DG	SLC1A4
DG	SLC1A6
DG	SLC1A7
DG	SLC25A10
DG	SLC25A12
DG	SLC25A13
DG	SLC25A15
DG	SLC25A18
DG	SLC25A2
DG	SLC25A22
DG	SLC25A4
DG	SLC32A1
DG	SLC36A1
DG	SLC38A3
DG	SLC3A1
DG	SLC6A14
DG	SLC6A5
DG	SLC6A7
DG	SLC6A9
DG	SLC7A1
DG	SLC7A11
DG	SLC7A2
DG	SLC7A3
DG	SLC7A4
DG	SLC7A8
DG	SLC7A9
DG	SMARCA5
DG	SMPD3
DG	SMPD4
DG	SOAT1
DG	SOAT2
DG	SOD2
DG	SPTLC1
DG	SPTLC2
DG	SP_1951
DG	SRC
DG	SRD5A2
DG	SRPK2
DG	SRR
DG	SUCLA2
DG	SUCLG1
DG	SUCLG2
DG	SUCNR1
DG	SULT2A1

DG	SULT2B1
DG	TARS
DG	TARS2
DG	TAT
DG	TGFBR2
DG	TH
DG	THNSL1
DG	TMLHE
DG	TPI1
DG	TRDMT1
DG	TREM1
DG	TUB
DG	TYMS
DG	TYR
DG	UBC
DG	UCKL1
DG	VARS
DG	VDR
DG	WARS
DG	WARS2
DG	XDH
DG	YARS
DG	YARS2
DG	YWHAE
DG	ABFA
DG	AGLA
DG	ALGC
DG	ALGD
DG	ALSB
DG	AMPC
DG	AMS
DG	AMY
DG	AMYA
DG	AMYE
DG	AMYM
DG	AMYP
DG	ANSB
DG	AQPZ
DG	ARAC
DG	ARAF
DG	ARGB
DG	ARGG
DG	AROB
DG	ASPA
DG	ASPC
DG	ATPE
DG	ATPF
DG	ATPH
DG	ATSA
DG	BFR
DG	BGL3

DG	BGLA
DG	BGLT
DG	BLA
DG	BLACTX-M-14
DG	BLACTX-M-27
DG	BLACTX-M-9A
DG	BOTB
DG	BUDC
DG	CAMC
DG	CBHA
DG	CC4
DG	CEL5A
DG	CELA
DG	CELA1
DG	CELB
DG	CELC307
DG	CELCCF
DG	CELCCG
DG	CELD
DG	CELS
DG	CENC
DG	CEX
DG	CFT
DG	CGT
DG	CHOB
DG	COBT
DG	CS1
DG	CSLB
DG	CTXB
DG	DADX
DG	DAPA
DG	DAPD
DG	DCTD
DG	DDH
DG	DEOA
DG	DHAK
DG	DLGD
DG	DMSA
DG	EBGA
DG	ECO
DG	ENGF
DG	ENO
DG	ENTB
DG	ENV
DG	FABI
DG	FDHF
DG	FHUA
DG	FMT
DG	FTSI
DG	FUCI
DG	GADA

DG	GAG-POL
DG	GALM
DG	GANB
DG	GDHB
DG	GFO
DG	GLCB
DG	GLK
DG	GLMS
DG	GLPF
DG	GLPG
DG	GLVA
DG	GPT
DG	GSHA
DG	GUDD
DG	HEMB
DG	HISD
DG	HLD
DG	HMP
DG	HMUO
DG	HUTH
DG	ICD
DG	ILVE
DG	KBL
DG	KSI
DG	LACG
DG	LACZ
DG	LAMB
DG	LECB
DG	LEUB
DG	LGTC
DG	LINB
DG	LPXA
DG	LUXS
DG	LYSA
DG	MAF
DG	MALE
DG	MALP
DG	MENC
DG	METE
DG	MEXA
DG	MGLB
DG	MOXC
DG	MRCA
DG	MRCB
DG	MRDA
DG	MRSA
DG	MTNN
DG	MURA
DG	MURE
DG	MURI
DG	MUTL

DG	MUTY
DG	MXIM
DG	NAHD
DG	NARG
DG	NARH
DG	NARI
DG	NEDA
DG	NIKA
DG	NOS
DG	NPLT
DG	NPRS
DG	NRDB
DG	NTPK
DG	OPPA
DG	OTSA
DG	PAB
DG	PBP1B
DG	PBP2A
DG	PBP3
DG	PBPA
DG	PBPC
DG	PCP
DG	PDP
DG	PENA
DG	PFLB
DG	PGMB
DG	PHNA
DG	PHR
DG	PLC
DG	POBA
DG	POL
DG	POLA
DG	PPGMK
DG	PTD
DG	PUFC
DG	PUFL
DG	PUFM
DG	PUHA
DG	PURF
DG	PURR
DG	PYP
DG	PYRC
DG	PYRD
DG	PYRDA
DG	PYRE
DG	RADH
DG	RBSK
DG	RHAA
DG	RMLC
DG	RSUA
DG	RUB

DG	RUVB
DG	SACB
DG	SCRY
DG	SDSA1
DG	SERA
DG	SHP
DG	SPG
DG	SPOII
DG	STX2EB
DG	STXB
DG	SUFS
DG	TALB
DG	TESA
DG	TETX
DG	TREZ
DG	TRPS
DG	TRPS2
DG	TRXB
DG	TVAI
DG	TVAII
DG	TYRS
DG	UBIC
DG	UDG
DG	ULAG
DG	UNG
DG	XLNA
DG	XYLA
DG	XYNA
DG	XYNY
DG	XYNZ
DG	ZWF
DS	72
DS	ACO2
DS	ADH1C
DS	AKR1C1
DS	AKR1C2
DS	AKR1D1
DS	ALAD
DS	ALDH5A1
DS	ALOX5
DS	AR
DS	ASPH
DS	ATP1A1
DS	ATP5A1
DS	ATP5B
DS	ATP5C1
DS	BBOX1
DS	BGLAP
DS	CA1
DS	CA12
DS	CA14

DS	CA2
DS	CA3
DS	CA4
DS	CA5A
DS	CA5B
DS	CA6
DS	CA7
DS	CA9
DS	CDA
DS	CDK6
DS	CES1
DS	COMT
DS	COX4I1
DS	COX5A
DS	COX5B
DS	COX6A2
DS	COX6B1
DS	COX6C
DS	COX7A1
DS	COX7B
DS	COX7C
DS	COX8A
DS	CSNK2A1
DS	DGKA
DS	DKFZP686P18130
DS	EFTUD1
DS	ESR1
DS	ESR2
DS	ESRRG
DS	F10
DS	F2
DS	F7
DS	F9
DS	FABP6
DS	FECH
DS	GABRA1
DS	GABRA2
DS	GABRA3
DS	GABRA4
DS	GABRA5
DS	GABRA6
DS	GABRB1
DS	GABRB2
DS	GABRB3
DS	GABRD
DS	GABRE
DS	GABRG1
DS	GABRG2
DS	GABRG3
DS	GABRP
DS	GABRQ

DS	GGCX
DS	GLRA3
DS	GRIN1
DS	GRIN2A
DS	GRIN2B
DS	GRIN2C
DS	GRIN2D
DS	GRIN3A
DS	GRIN3B
DS	HBA1
DS	HBB
DS	HCK
DS	HIBCH
DS	HMGCR
DS	HSD11B1
DS	HSD17B1
DS	HSD17B6
DS	IGHG1
DS	IGHG2
DS	ITGB2
DS	LCN2
DS	LIP3
DS	LSS
DS	MIF
DS	MT-CO1
DS	MT-CO2
DS	MT-CO3
DS	NCOA1
DS	NCOA2
DS	NQO1
DS	NQO2
DS	NR1H4
DS	NR1I2
DS	NR1I3
DS	NR3C1
DS	NR3C2
DS	OXCT1
DS	OXCT2
DS	P3H1
DS	P3H2
DS	P3H3
DS	P4HA1
DS	P4HA2
DS	PGR
DS	PIK3CA
DS	PIK3CG
DS	PIK3R1
DS	PIM1
DS	PLA2G1B
DS	PLK1
DS	PLOD1

DS	PLOD3
DS	PPARA
DS	PPP1CC
DS	PPP2CA
DS	PPP2CB
DS	PRKACA
DS	PRKCA
DS	PRKCB
DS	PRLR
DS	PROC
DS	PROS1
DS	PROZ
DS	PTGS1
DS	PTK2B
DS	PYGM
DS	RORA
DS	SDHA
DS	SDHB
DS	SDHC
DS	SDHD
DS	SEC14L2
DS	SEC14L3
DS	SEC14L4
DS	SIGMAR1
DS	SLC13A1
DS	SLC13A2
DS	SLC13A3
DS	SLC25A10
DS	SQLE
DS	SRD5A2
DS	STK17B
DS	SUCLA2
DS	SUCLG1
DS	SUCLG2
DS	SUCNR1
DS	SULT2A1
DS	SULT2B1
DS	SYK
DS	TMLHE
DS	TOP2A
DS	TYR
DS	UGT3A1
DS	VDR
DS	VKORC1
DS	VKORC1L1
DS	YWHAE
DS	ACRB
DS	ACTIII
DS	ALDA
DS	ASPC
DS	BPHC

DS	CAT
DS	CAT3
DS	CATA
DS	CBH
DS	CCRA
DS	CHOB
DS	COBT
DS	CTAC
DS	CTAD
DS	DAPD
DS	DHBE
DS	DOXG
DS	FABZ
DS	FRDA
DS	FRDB
DS	FRDC
DS	FUSA
DS	GADA
DS	GAG-POL
DS	HEMB
DS	HUTH
DS	ILVE
DS	KSI
DS	MNDD
DS	MVAA
DS	NAHD
DS	NCSA
DS	PAC
DS	PCAG
DS	PCAH
DS	POLA
DS	PPCA
DS	PYP
DS	TRXB
DS	TTGR
DS	UBIC
DS	XYNY
DS	XYNZ
GC	72
GC	9
GC	AADACL2
GC	ABO
GC	ACHE
GC	ADCY2
GC	ADCY5
GC	ADH1C
GC	AKR1C1
GC	AKR1C2
GC	AKR1C3
GC	AKR1D1
GC	ALDH2

GC	ALOX5
GC	AMY1A
GC	AMY2A
GC	AMY2B
GC	ANXA1
GC	APH1A
GC	AR
GC	ARG1
GC	ATP1A1
GC	ATP5A1
GC	ATP5B
GC	ATP5C1
GC	BCHE
GC	CA2
GC	CDA
GC	CDIPT
GC	CDK6
GC	CES1
GC	CHRM2
GC	CHRNA1
GC	CHRNA10
GC	CHRNA2
GC	CHRNA3
GC	CHRNA4
GC	CHRNA5
GC	CHRNA6
GC	CHRNA7
GC	CHRNA9
GC	CHRNB1
GC	CHRNB2
GC	CHRNB3
GC	CHRNB4
GC	CHRND
GC	CHRNE
GC	CHRNG
GC	COMT
GC	COX4I1
GC	COX5A
GC	COX5B
GC	COX6A2
GC	COX6B1
GC	COX6C
GC	COX7A1
GC	COX7B
GC	COX7C
GC	COX8A
GC	CSNK2A1
GC	CTNNB1
GC	CYP27B1
GC	DKFZP686P18130
GC	DRD2

GC	EDNRA
GC	EFTUD1
GC	ESR1
GC	ESR2
GC	ESRRG
GC	FABP6
GC	FECH
GC	GABRA1
GC	GABRA2
GC	GABRA3
GC	GABRA4
GC	GABRA5
GC	GABRA6
GC	GABRB1
GC	GABRB2
GC	GABRB3
GC	GABRD
GC	GABRE
GC	GABRG1
GC	GABRG2
GC	GABRG3
GC	GABRP
GC	GABRQ
GC	GAPDHS
GC	GBA
GC	GCK
GC	GLO1
GC	GLRA3
GC	GLT6D1
GC	GLTP
GC	GNAS
GC	GNPDA1
GC	GRIN1
GC	GRIN2A
GC	GRIN2B
GC	GRIN2C
GC	GRIN2D
GC	GRIN3A
GC	GRIN3B
GC	HCK
GC	HIBCH
GC	HK1
GC	HMGCR
GC	HSD11B1
GC	HSD17B1
GC	HSD17B11
GC	HSPA5
GC	HTR2A
GC	HTR3A
GC	IFNB1
GC	IGHG1

GC	IGHG2
GC	IKBKB
GC	ITGB2
GC	KRTAP5-2
GC	KRTAP5-3
GC	LCN2
GC	LCT
GC	LCTL
GC	LGALS2
GC	LGALS7
GC	LIP3
GC	LSS
GC	MAN1B1
GC	MAN2A1
GC	MB
GC	MBL2
GC	MED1
GC	MSDC
GC	MT-CO1
GC	MT-CO2
GC	MT-CO3
GC	MTTP
GC	NCAN
GC	NCOA1
GC	NCOA2
GC	NFKB1
GC	NFKB2
GC	NFKBIA
GC	NOS2
GC	NR0B1
GC	NR1H4
GC	NR1I2
GC	NR1I3
GC	NR3C1
GC	NR3C2
GC	NUDT9
GC	OPRD1
GC	OPRK1
GC	OPRM1
GC	PGR
GC	PIK3CG
GC	PIM1
GC	PLA2G1B
GC	PLA2G2E
GC	PPARA
GC	PPP1CC
GC	PRKAA1
GC	PRKAA2
GC	PRKAB1
GC	PRKAB2
GC	PRKAG1

GC	PRKAG2
GC	PRKAG3
GC	PRLR
GC	PSENEN
GC	PTGS1
GC	PTGS2
GC	PTK2B
GC	PYGL
GC	PYGM
GC	RORA
GC	RPS6KA3
GC	SFTPD
GC	SIGLEC1
GC	SIGMAR1
GC	SLC18A2
GC	SMARCA5
GC	SOAT1
GC	SOAT2
GC	STK17B
GC	SULT2A1
GC	SULT2B1
GC	TOP2A
GC	TP53
GC	UGT3A1
GC	VDR
GC	YWHAE
GC	ACRB
GC	ACTIII
GC	AGLA
GC	AGUA
GC	ALDA
GC	ALSB
GC	AMS
GC	AMY
GC	AMYA
GC	AMYE
GC	AMYM
GC	AMYP
GC	ASPA
GC	BGLA
GC	BOTB
GC	BUDC
GC	CAT
GC	CAT3
GC	CBH
GC	CEL5A
GC	CELA
GC	CELA1
GC	CELB
GC	CELC307
GC	CELCCF

GC	CELCCG
GC	CELD
GC	CELS
GC	CENC
GC	CEX
GC	CGT
GC	CHOB
GC	CSLA
GC	CSLB
GC	CTAC
GC	CTAD
GC	CTXB
GC	CYP158A2
GC	DMSA
GC	ECO
GC	ENGF
GC	ENTB
GC	ETHR
GC	FABG3
GC	FABI
GC	FABZ
GC	FHUA
GC	FOLA
GC	FUSA
GC	GAG-POL
GC	GALM
GC	GANB
GC	GDHB
GC	GFO
GC	GLK
GC	KSI
GC	LACZ
GC	LAMB
GC	LECB
GC	LGTC
GC	MALE
GC	MALP
GC	MGLB
GC	MOXC
GC	MUTY
GC	MVAA
GC	NAHD
GC	NCSA
GC	NEDA
GC	NPLT
GC	PLCA
GC	PPCA
GC	PPGMK
GC	PUNA
GC	RBSB
GC	RBSD

GC	SPOII
GC	STX2EB
GC	STXB
GC	TETX
GC	TREZ
GC	TTGR
GC	TVAI
GC	TVAII
GC	UBIC
GC	XLNA
GC	XYL
GC	XYLA
GC	XYN10C
GC	XYNA
GC	XYNC
GC	XYNY
GC	XYNZ
GC	YEDY
HH	AASS
HH	ABAT
HH	ABL1
HH	ACADSB
HH	ACOT13
HH	ACOX1
HH	ACSL3
HH	ACSL4
HH	ADH1B
HH	ADRA2A
HH	AKR1C1
HH	AKR1C2
HH	ALDH1A1
HH	ALDH1A2
HH	ALDH5A1
HH	APH1A
HH	AR
HH	ARF1
HH	ARF6
HH	ARG1
HH	ARG2
HH	ATP1A1
HH	ATP6V1C1
HH	B3GAT1
HH	CALM1
HH	CDA
HH	CTRB1
HH	CYP27B1
HH	CYP2C8
HH	DAO
HH	DBI
HH	DDC
HH	DDX6

HH	DRD1
HH	DRD2
HH	DRD3
HH	DRD4
HH	DRD5
HH	ECI2
HH	ELOVL4
HH	EPOR
HH	ESR1
HH	ESR2
HH	FADS1
HH	FADS2
HH	FFAR1
HH	FKBP1A
HH	FURIN
HH	GABRA1
HH	GABRA2
HH	GABRA3
HH	GABRA4
HH	GABRA5
HH	GABRA6
HH	GABRB1
HH	GABRB2
HH	GABRB3
HH	GABRD
HH	GABRE
HH	GABRG1
HH	GABRG2
HH	GABRG3
HH	GABRP
HH	GABRQ
HH	GATM
HH	GLRA3
HH	GLTP
HH	GM2A
HH	GPRC5A
HH	GRIN1
HH	GRIN2A
HH	GRIN2B
HH	GRIN2C
HH	GRIN2D
HH	GRIN3A
HH	GRIN3B
HH	GSTP1
HH	GUCA1A
HH	HAO1
HH	HDAC2
HH	HDAC9
HH	HIF1AN
HH	HNF4A
HH	HNF4G

HH	HPGDS
HH	HSD17B1
HH	IGHG1
HH	IGHG2
HH	IGKC
HH	INS
HH	ISYNA1
HH	ITPR1
HH	IYD
HH	KARS
HH	LALBA
HH	LCN2
HH	LCN9
HH	LIP3
HH	LSS
HH	LY96
HH	MIF
HH	MT1417
HH	NAGA
HH	NCOA1
HH	NCOA2
HH	NR0B1
HH	NR1I2
HH	NR1I3
HH	NR3C1
HH	OAT
HH	OAZ1
HH	OAZ2
HH	OAZ3
HH	ODC1
HH	OGDH
HH	OTC
HH	PAEP
HH	PAPSS1
HH	PGR
HH	PKIA
HH	PLA2G1B
HH	PLA2G2A
HH	PLA2G2D
HH	PLA2G2E
HH	PMP2
HH	PPARA
HH	PPARD
HH	PPARG
HH	PPP1CC
HH	PPP3CA
HH	PPP3R1
HH	PPT1
HH	PRKACA
HH	PRSS1
HH	PSENEN

HH	PTGS1
HH	PTGS2
HH	PVR
HH	RARA
HH	RARB
HH	RARG
HH	RARRES1
HH	RCVRN
HH	RHO
HH	RORA
HH	RXRA
HH	RXRB
HH	RXRG
HH	RV0233
HH	S100B
HH	SCN10A
HH	SCN11A
HH	SCN1A
HH	SCN1B
HH	SCN2A
HH	SCN2B
HH	SCN3A
HH	SCN3B
HH	SCN4A
HH	SCN4B
HH	SCN5A
HH	SCN7A
HH	SCN8A
HH	SCN9A
HH	SEC14L2
HH	SIGMAR1
HH	SLC25A15
HH	SLC25A2
HH	SLC7A1
HH	SLC7A2
HH	SLC7A3
HH	SLC7A4
HH	SLC8A1
HH	SOD2
HH	SP_1951
HH	SULT2A1
HH	SULT2B1
HH	TAT
HH	TGFBR2
HH	TH
HH	TLR4
HH	TRAPPC3
HH	TRDMT1
HH	TREM1
HH	TRPV1
HH	VDR

HH	YARS
HH	YARS2
HH	YWHAE
HH	ANSB
HH	ARGG
HH	AROQ
HH	ASPC
HH	BFR
HH	BGLA
HH	BPHC
HH	CATA
HH	CC4
HH	CELCCF
HH	CELCCG
HH	CHOB
HH	COBT
HH	CUMD
HH	CYP102A1
HH	CYPC
HH	DADX
HH	DAPA
HH	DCTD
HH	DHBE
HH	DLGD
HH	DOXG
HH	ENO
HH	ENV
HH	EST
HH	FABB
HH	FADR
HH	FHUA
HH	FUCI
HH	GAG
HH	GAG-POL
HH	GLCB
HH	GLPF
HH	HISD
HH	HUTH
HH	KCSA
HH	KSI
HH	LIPB
HH	LPXA
HH	LPXC
HH	LUXF
HH	LUXS
HH	LYSA
HH	MALE
HH	MDLB
HH	METE
HH	MEXA
HH	MNDD

HH	MUTL
HH	MUTY
HH	NAHD
HH	NEF
HH	NIKA
HH	NRDB
HH	PAB
HH	PAC
HH	PCAG
HH	PCAH
HH	PCP
HH	PFLB
HH	PHNA
HH	PYP
HH	RHAA
HH	RMLC
HH	SERA
HH	TALB
HH	TCP14
HH	TESA
HH	TONB
HH	TYNA
HH	TYRS
HH	XYLA
HH	XYNY
HH	XYNZ
JG	AKR1C1
JG	AKR1C2
JG	ALOX5
JG	ANXA1
JG	AR
JG	ATP1A1
JG	CDA
JG	CYP27B1
JG	ESR1
JG	ESR2
JG	GABRA1
JG	GABRA2
JG	GABRA3
JG	GABRA4
JG	GABRA5
JG	GABRA6
JG	GABRB1
JG	GABRB2
JG	GABRB3
JG	GABRD
JG	GABRE
JG	GABRG1
JG	GABRG2
JG	GABRG3
JG	GABRP

JG	GABRQ
JG	GLRA3
JG	GRIN1
JG	GRIN2A
JG	GRIN2B
JG	GRIN2C
JG	GRIN2D
JG	GRIN3A
JG	GRIN3B
JG	HMGCR
JG	HSD11B1
JG	HSD17B1
JG	LIP3
JG	LSS
JG	MED1
JG	NCOA1
JG	NCOA2
JG	NOS2
JG	NR0B1
JG	NR1I2
JG	NR1I3
JG	NR3C1
JG	NR3C2
JG	PGR
JG	PPARA
JG	PPP1CC
JG	PTGS1
JG	RORA
JG	SIGMAR1
JG	SRD5A1
JG	SULT2A1
JG	SULT2B1
JG	VDR
JG	YWHAE
JG	CAT
JG	CAT3
JG	CHOB
JG	FUSA
JG	KSI
JG	MVAA
NX	AADAT
NX	AARS
NX	AARS2
NX	AASS
NX	ABAT
NX	ACY1
NX	ACY3
NX	ADH1B
NX	ADH1C
NX	ADSS
NX	ADSSL1

NX	AGXT
NX	AGXT2
NX	AKR1C1
NX	AKR1C2
NX	ALAS1
NX	ALAS2
NX	ALDH18A1
NX	ANXA1
NX	AR
NX	ARF1
NX	ARG1
NX	ARG2
NX	ASL
NX	ASNS
NX	ASPA
NX	ASPH
NX	ASRGL1
NX	ASS
NX	ASS1
NX	ATP6V1C1
NX	AZIN2
NX	B3GAT1
NX	BAAT
NX	BCAT1
NX	BCAT2
NX	BGLAP
NX	BHMT
NX	CAD
NX	CAMP
NX	CARS
NX	CARS2
NX	CBS
NX	CCBL1
NX	CCBL2
NX	CDA
NX	CDO-1
NX	CDO1
NX	CES1
NX	CKM
NX	COX4I1
NX	COX5A
NX	COX5B
NX	COX6A2
NX	COX6B1
NX	COX6C
NX	COX7A1
NX	COX7B
NX	COX7C
NX	COX8A
NX	CPA1
NX	CPQ

NX	CRH
NX	CSAD
NX	CTH
NX	CTPS1
NX	CTSB
NX	CXCL10
NX	DAO
NX	DARS
NX	DARS2
NX	DDX6
NX	DKFZP686P18130
NX	DNPEP
NX	DPP4
NX	DRD1
NX	DRD2
NX	DRD3
NX	DRD4
NX	DRD5
NX	E
NX	EARS2
NX	EFTUD1
NX	EGF
NX	ENPEP
NX	EPOR
NX	EPRS
NX	ESR1
NX	ESR2
NX	ESRRG
NX	F10
NX	F2
NX	FABP6
NX	FARS2
NX	FARSA
NX	FARSB
NX	FECH
NX	FOLH1
NX	FPGS
NX	FTCD
NX	GABRA1
NX	GABRA2
NX	GABRA3
NX	GABRA4
NX	GABRA5
NX	GABRA6
NX	GABRB1
NX	GABRB2
NX	GABRB3
NX	GABRD
NX	GABRE
NX	GABRG1
NX	GABRG2

NX	GABRG3
NX	GABRP
NX	GABRQ
NX	GAD1
NX	GAD2
NX	GAMT
NX	GARS
NX	GATB
NX	GATM
NX	GCAT
NX	GCLC
NX	GCLM
NX	GCSH
NX	GGCX
NX	GIG18
NX	GLDC
NX	GLRA1
NX	GLRA2
NX	GLRA3
NX	GLRB
NX	GLS
NX	GLS2
NX	GLUD1
NX	GLUD2
NX	GLUL
NX	GLYAT
NX	GLYATL1
NX	GLYATL2
NX	GMPS
NX	GNMT
NX	GOT1
NX	GOT2
NX	GPR18
NX	GPT
NX	GPT2
NX	GRIA1
NX	GRIA2
NX	GRIA3
NX	GRIA4
NX	GRID1
NX	GRID2
NX	GRIK1
NX	GRIK2
NX	GRIK3
NX	GRIK4
NX	GRIK5
NX	GRIN1
NX	GRIN2A
NX	GRIN2B
NX	GRIN2C
NX	GRIN2D

NX	GRIN3A
NX	GRIN3B
NX	GRM1
NX	GRM4
NX	GRM7
NX	GRM8
NX	GSS
NX	GSTM1
NX	GSTP1
NX	HIF1AN
NX	HPGDS
NX	HSD11B1
NX	HSD17B1
NX	IGHG1
NX	IGHG2
NX	ISYNA1
NX	ITPR1
NX	IYD
NX	KYNU
NX	L3HYPDH
NX	LARS
NX	LARS2
NX	LCMT1
NX	LCMT2
NX	LGSN
NX	LSS
NX	LYZ
NX	ME2
NX	MGMT
NX	MT-CO1
NX	MT-CO2
NX	MT-CO3
NX	NADSYN1
NX	NAGA
NX	NAGS
NX	NCOA1
NX	NCOA2
NX	NFS1
NX	NME1
NX	NOS1
NX	NOS2
NX	NOS3
NX	NPY
NX	NR0B1
NX	NR1H4
NX	NR1I2
NX	NR1I3
NX	NR3C1
NX	NR3C2
NX	OAT
NX	OAZ1

NX	OAZ2
NX	OAZ3
NX	OPLAH
NX	OTC
NX	P3H1
NX	P3H2
NX	P3H3
NX	P4HA1
NX	P4HA2
NX	P5CR2
NX	PAEP
NX	PAH
NX	PAICS
NX	PAPSS1
NX	PARS2
NX	PCCB
NX	PFAS
NX	PGR
NX	PHYKPL
NX	PIN1
NX	PIPOX
NX	PKIA
NX	PLA2G1B
NX	PLA2G2E
NX	PPARA
NX	PPAT
NX	PPIA
NX	PPIB
NX	PPIC
NX	PPIF
NX	PPIG
NX	PPIH
NX	PPP1CC
NX	PRKACA
NX	PRLR
NX	PRODH
NX	PROSC
NX	PRSS1
NX	PSAT1
NX	PTPN1
NX	PYCR1
NX	PYCR2
NX	PYCRL
NX	RNASE1
NX	RORA
NX	SARS
NX	SDS
NX	SERPINB3
NX	SHMT1
NX	SHMT2
NX	SIGMAR1

NX	SLC19A3
NX	SLC1A1
NX	SLC1A2
NX	SLC1A3
NX	SLC1A4
NX	SLC1A6
NX	SLC1A7
NX	SLC25A12
NX	SLC25A13
NX	SLC25A15
NX	SLC25A18
NX	SLC25A2
NX	SLC25A22
NX	SLC32A1
NX	SLC36A1
NX	SLC6A14
NX	SLC6A5
NX	SLC6A7
NX	SLC6A9
NX	SLC7A1
NX	SLC7A11
NX	SLC7A2
NX	SLC7A3
NX	SLC7A4
NX	SLC7A8
NX	SOD2
NX	SPTLC1
NX	SPTLC2
NX	SP_1951
NX	SRC
NX	SRR
NX	SULT2A1
NX	SULT2B1
NX	TARS
NX	TARS2
NX	TAT
NX	TGFBR2
NX	TH
NX	THNSL1
NX	TPI1
NX	TRDMT1
NX	TREM1
NX	VARS
NX	VDR
NX	WARS
NX	WARS2
NX	YARS
NX	YARS2
NX	ACRB
NX	ALDA
NX	ANSB

NX	ARGB
NX	ARGG
NX	ATPF
NX	ATSA
NX	BFR
NX	BGLA
NX	CAT
NX	CAT3
NX	CBH
NX	CC4
NX	CELCCF
NX	CELCCG
NX	CHOB
NX	CS1
NX	CTAC
NX	CTAD
NX	DAPD
NX	DCTD
NX	DDH
NX	DLGD
NX	ENO
NX	ENV
NX	FDHF
NX	FUCI
NX	FUSA
NX	GAG-POL
NX	GLCB
NX	GLPF
NX	GUDD
NX	HISD
NX	ICD
NX	ILVE
NX	KBL
NX	KSI
NX	LEUB
NX	LINB
NX	LPXA
NX	MALE
NX	MENC
NX	METE
NX	MEXA
NX	MRSA
NX	MURA
NX	MURE
NX	MURI
NX	MUTL
NX	MUTY
NX	NCSA
NX	NIKA
NX	NPRS
NX	NRDB

NX	OPPA
NX	PCP
NX	PFLB
NX	PHNA
NX	POL
NX	PPCA
NX	PURF
NX	RHAA
NX	RMLC
NX	SERA
NX	SHP
NX	SUFS
NX	TRPS
NX	TRPS2
NX	TYRS
NX	XYLA
SM	AKR1C1
SM	AKR1C2
SM	AR
SM	ATP1A1
SM	CTRB1
SM	ESR1
SM	ESR2
SM	GABRA1
SM	GABRA2
SM	GABRA3
SM	GABRA4
SM	GABRA5
SM	GABRA6
SM	GABRB1
SM	GABRB2
SM	GABRB3
SM	GABRD
SM	GABRE
SM	GABRG1
SM	GABRG2
SM	GABRG3
SM	GABRP
SM	GABRQ
SM	GAPDHS
SM	GLRA3
SM	GRIN1
SM	GRIN2A
SM	GRIN2B
SM	GRIN2C
SM	GRIN2D
SM	GRIN3A
SM	GRIN3B
SM	HSD17B1
SM	LCN2
SM	LIP3

SM	LSS
SM	MIF
SM	NCOA2
SM	NR1I2
SM	NR1I3
SM	NR3C1
SM	PGR
SM	PPARA
SM	PPP1CC
SM	RORA
SM	SIGMAR1
SM	SULT2A1
SM	SULT2B1
SM	VDR
SM	YWHAE
SM	ASST
SM	CHOB
SM	DHBE
SM	HUTH
SM	KSI
SM	MNDD
SM	NAHD
SM	PAC
SM	PCAG
SM	PCAH
SM	PYP
SM	UBIC
SM	XYNY
SM	XYNZ
TR	9
TR	ABL1
TR	ABO
TR	ACSL3
TR	ACSL4
TR	ACTA1
TR	ADH1B
TR	AKR1C1
TR	AKR1C2
TR	AMY1A
TR	AMY2A
TR	AMY2B
TR	AR
TR	ARF1
TR	ARF6
TR	ATOX1
TR	ATP1A1
TR	CALM1
TR	CDA
TR	CYP27B1
TR	CYP2B6
TR	CYP2C8

TR	DAO
TR	ECI2
TR	ELOVL4
TR	ESR1
TR	ESR2
TR	FADS1
TR	FADS2
TR	FFAR1
TR	FHIT
TR	FKBP1A
TR	FURIN
TR	GABRA1
TR	GABRA2
TR	GABRA3
TR	GABRA4
TR	GABRA5
TR	GABRA6
TR	GABRB1
TR	GABRB2
TR	GABRB3
TR	GABRD
TR	GABRE
TR	GABRG1
TR	GABRG2
TR	GABRG3
TR	GABRP
TR	GABRQ
TR	GCK
TR	GLRA3
TR	GLT6D1
TR	GLTP
TR	GM2A
TR	GNPDA1
TR	GRIN1
TR	GRIN2A
TR	GRIN2B
TR	GRIN2C
TR	GRIN2D
TR	GRIN3A
TR	GRIN3B
TR	GSTM1
TR	GSTP1
TR	GUCA1A
TR	HK1
TR	HNF4A
TR	HNF4G
TR	HPGDS
TR	HSD17B1
TR	IFNB1
TR	IGHG2
TR	IGKC

TR	INS
TR	ISYNA1
TR	ITPR1
TR	KRTAP5-2
TR	KRTAP5-3
TR	LALBA
TR	LCT
TR	LCTL
TR	LGALS2
TR	LGALS7
TR	LIP3
TR	LSS
TR	LY96
TR	LYZ
TR	MB
TR	MBL2
TR	MT1417
TR	NAGA
TR	NCAN
TR	NCOA1
TR	NCOA2
TR	NR1I2
TR	NR1I3
TR	NR3C1
TR	NUDT9
TR	PAEP
TR	PAPSS1
TR	PGR
TR	PKIA
TR	PLA2G1B
TR	PLA2G2A
TR	PLA2G2D
TR	PLA2G2E
TR	PMP2
TR	PPARA
TR	PPARD
TR	PPARG
TR	PPP1CC
TR	PPP3CA
TR	PPP3R1
TR	PPT1
TR	PRKACA
TR	PTGS1
TR	PTGS2
TR	PVR
TR	PYGL
TR	PYGM
TR	RCVRN
TR	RHO
TR	RORA
TR	RTCB

TR	RV0233
TR	S100B
TR	SEC14L2
TR	SFTPD
TR	SIGLEC1
TR	SIGMAR1
TR	SLC8A1
TR	SMARCA5
TR	SULT2A1
TR	SULT2B1
TR	TGFBR2
TR	TLR4
TR	TPI1
TR	TRAPPC3
TR	TRDMT1
TR	TRPV1
TR	VDR
TR	WARS
TR	WARS2
TR	YWHAE
TR	ABFA
TR	AGLA
TR	ALGD
TR	ALSB
TR	AMPC
TR	AMS
TR	AMY
TR	AMYA
TR	AMYE
TR	AMYM
TR	AMYP
TR	ARAC
TR	ARAF
TR	ARGG
TR	AROQ
TR	ASPA
TR	BFR
TR	BGL3
TR	BGLA
TR	BLA
TR	BLACTX-M-14
TR	BLACTX-M-27
TR	BLACTX-M-9A
TR	BOTB
TR	BUDC
TR	CBHA
TR	CC4
TR	CEL5A
TR	CELA
TR	CELA1
TR	CELB

TR	CELC307
TR	CELCCF
TR	CELCCG
TR	CELD
TR	CELS
TR	CENC
TR	CEX
TR	CFT
TR	CGT
TR	CHOB
TR	CSLB
TR	CTXB
TR	CUMD
TR	CYP102A1
TR	CYPC
TR	DCTD
TR	DMSA
TR	EBGA
TR	ECO
TR	ENGF
TR	ENO
TR	ENTB
TR	FABB
TR	FABI
TR	FADR
TR	FHUA
TR	FUCI
TR	GAG
TR	GAG-POL
TR	GALM
TR	GANB
TR	GDHB
TR	GFO
TR	GLCB
TR	GLK
TR	GLPF
TR	HISD
TR	HMUO
TR	KCSA
TR	KSI
TR	LACZ
TR	LAMB
TR	LECB
TR	LGTC
TR	LIPB
TR	LPXC
TR	LUXF
TR	MAF
TR	MALE
TR	MALP
TR	METE

TR	MEXA
TR	MGLB
TR	MOXC
TR	MUTL
TR	MUTY
TR	NEDA
TR	NEF
TR	NOS
TR	NPLT
TR	NRDB
TR	PAB
TR	PFLB
TR	PPGMK
TR	RBSK
TR	RHAA
TR	SACB
TR	SCRY
TR	SPOII
TR	STX2EB
TR	STXB
TR	TCP14
TR	TESA
TR	TETX
TR	TONB
TR	TREZ
TR	TRPS
TR	TRPS2
TR	TVAI
TR	TVAII
TR	XLNA
TR	XYLA
TR	XYNA
WY	ABL1
WY	ACHE
WY	ACSL3
WY	ACSL4
WY	ADRA2A
WY	ADRA2B
WY	ADRA2C
WY	AKR1D1
WY	AR
WY	ARF1
WY	ARF6
WY	CALM1
WY	CALY
WY	CHRNA2
WY	CYP2C8
WY	DRD1
WY	DRD2
WY	DRD3
WY	DRD4

WY	DRD5
WY	ECI2
WY	ELOVL4
WY	ESR2
WY	FADS1
WY	FADS2
WY	FFAR1
WY	FKBP1A
WY	FURIN
WY	GABRA1
WY	GABRA2
WY	GABRA3
WY	GABRA4
WY	GABRA5
WY	GABRA6
WY	GLTP
WY	GM2A
WY	GUCA1A
WY	HNF4A
WY	HNF4G
WY	HSD17B1
WY	HSD17B11
WY	HTR1A
WY	HTR1B
WY	HTR1D
WY	HTR2A
WY	HTR2B
WY	HTR2C
WY	HTR3A
WY	IGHG2
WY	INS
WY	LALBA
WY	LIP3
WY	LSS
WY	LY96
WY	MT1417
WY	NCOA1
WY	NCOA2
WY	NR1I3
WY	OPRK1
WY	PAEP
WY	PKIA
WY	PLA2G1B
WY	PLA2G2A
WY	PLA2G2D
WY	PLA2G2E
WY	PMP2
WY	PPARD
WY	PPARG
WY	PPP3CA
WY	PPP3R1

WY	PPT1
WY	PRKACA
WY	PTGS1
WY	PTGS2
WY	PVR
WY	RCVRN
WY	RHO
WY	RXRA
WY	RV0233
WY	SEC14L2
WY	SLC8A1
WY	SULT2A1
WY	TLR4
WY	TRAPPC3
WY	TRPA1
WY	TRPM8
WY	TRPV1
WY	TRPV3
WY	VDR
WY	CAMC
WY	CYP102A1
WY	CYPC
WY	FABB
WY	FABG3
WY	FADR
WY	FHUA
WY	GAG
WY	GAG-POL
WY	LIPB
WY	LPXC
WY	LUXF
WY	NEF
WY	PAB
WY	TCP14
WY	TESA
WY	TONB
XF	ADH1B
XF	AKR1C1
XF	AKR1C2
XF	AKR1C3
XF	AKR1D1
XF	AR
XF	ARF1
XF	CDA
XF	CYP17A1
XF	CYP19A1
XF	CYP27B1
XF	ESR1
XF	ESR2
XF	GABRA1
XF	GABRA2

XF	GABRA3
XF	GABRA4
XF	GABRA5
XF	GABRA6
XF	GABRB1
XF	GABRB2
XF	GABRB3
XF	GABRD
XF	GABRE
XF	GABRG1
XF	GABRG2
XF	GABRG3
XF	GABRP
XF	GABRQ
XF	GRIN1
XF	GRIN2A
XF	GRIN2B
XF	GRIN2C
XF	GRIN2D
XF	GRIN3A
XF	GRIN3B
XF	GSTP1
XF	HPGDS
XF	HSD17B1
XF	HSD17B11
XF	HSD3B1
XF	IGHG2
XF	ISYNA1
XF	ITPR1
XF	LCN9
XF	LIP3
XF	LSS
XF	MED1
XF	NAGA
XF	NCOA1
XF	NCOA2
XF	NR1I2
XF	NR1I3
XF	NR3C1
XF	NR3C2
XF	OPRK1
XF	PAEP
XF	PAPSS1
XF	PGR
XF	PLA2G2E
XF	PPARA
XF	RORA
XF	SIGMAR1
XF	SULT2A1
XF	SULT2B1
XF	TGFBR2

XF	TRDMT1
XF	VDR
XF	ARGG
XF	BFR
XF	BGLA
XF	CAMC
XF	CC4
XF	CELCCF
XF	CELCCG
XF	CHOB
XF	DCTD
XF	DHAK
XF	ENO
XF	ERYF
XF	FABG3
XF	FUCI
XF	GLCB
XF	GLPF
XF	HISD
XF	KSI
XF	MALE
XF	METE
XF	MEXA
XF	MUTL
XF	MUTY
XF	NRDB
XF	ONR
XF	PFLB
XF	RHAA
XF	XYLA
YHS	ACHE
YHS	ADRA2A
YHS	ADRA2B
YHS	ADRA2C
YHS	AKR1C1
YHS	AKR1C2
YHS	AR
YHS	CALY
YHS	CHRM2
YHS	CHRNA2
YHS	CHRNA3
YHS	CHRNA4
YHS	CHRNA7
YHS	CHRNB2
YHS	CHRNB4
YHS	CYBA
YHS	CYBB
YHS	DRD1
YHS	DRD2
YHS	DRD3
YHS	DRD4

YHS	DRD5
YHS	ESR1
YHS	ESR2
YHS	GABRA1
YHS	GABRA2
YHS	GABRA3
YHS	GABRA4
YHS	GABRA5
YHS	GABRA6
YHS	GABRB1
YHS	GABRB2
YHS	GABRB3
YHS	GABRD
YHS	GABRE
YHS	GABRG1
YHS	GABRG2
YHS	GABRG3
YHS	GABRP
YHS	GABRQ
YHS	GRIN1
YHS	GRIN2A
YHS	GRIN2B
YHS	GRIN2C
YHS	GRIN2D
YHS	GRIN3A
YHS	GRIN3B
YHS	HSD17B1
YHS	HTR1A
YHS	HTR1B
YHS	HTR1D
YHS	HTR2A
YHS	HTR2B
YHS	HTR2C
YHS	HTR3A
YHS	LIP3
YHS	LSS
YHS	NCF1
YHS	NCF2
YHS	NCF4
YHS	NCOA2
YHS	NR1I2
YHS	NR1I3
YHS	NR3C1
YHS	OPRD1
YHS	OPRK1
YHS	OPRM1
YHS	PGR
YHS	PGRMC1
YHS	PPARA
YHS	RAC1
YHS	RAC2

YHS	RORA
YHS	SIGMAR1
YHS	SLC18A2
YHS	SLC6A2
YHS	SLC6A4
YHS	SULT2A1
YHS	SULT2B1
YHS	TOP1
YHS	VDR
YHS	CHOB
YHS	KSI
YHS	QACR
ZQ	72
ZQ	9
ZQ	ABCA1
ZQ	ABCB11
ZQ	ABCC8
ZQ	ABCC9
ZQ	ABO
ZQ	ACHE
ZQ	ACOT13
ZQ	ADRA1A
ZQ	ADRA1B
ZQ	ADRA1D
ZQ	ADRA2A
ZQ	ADRA2B
ZQ	ADRA2C
ZQ	ADRB1
ZQ	ADRB2
ZQ	ADRB3
ZQ	AKR1C3
ZQ	ALDH2
ZQ	AMY1A
ZQ	AMY2A
ZQ	AMY2B
ZQ	ATF1
ZQ	ATF2
ZQ	ATF3
ZQ	ATF4
ZQ	ATF5
ZQ	ATF6
ZQ	ATF7
ZQ	ATP1A1
ZQ	ATP5A1
ZQ	ATP5B
ZQ	ATP5C1
ZQ	CA1
ZQ	CA12
ZQ	CA14
ZQ	CA2
ZQ	CA3

ZQ	CA4
ZQ	CA5A
ZQ	CA5B
ZQ	CA6
ZQ	CA7
ZQ	CA9
ZQ	CDK6
ZQ	CFTR
ZQ	COMT
ZQ	CPT1A
ZQ	CSNK2A1
ZQ	CYP2A6
ZQ	CYP2B6
ZQ	DBI
ZQ	DRD1
ZQ	ESR1
ZQ	ESR2
ZQ	FOS
ZQ	GCK
ZQ	GLT6D1
ZQ	GLTP
ZQ	GNPDA1
ZQ	HCK
ZQ	HIBCH
ZQ	HK1
ZQ	IFNB1
ZQ	IGHG1
ZQ	IL2
ZQ	JDP2
ZQ	JUN
ZQ	KCNJ1
ZQ	KCNJ11
ZQ	KCNJ5
ZQ	KCNJ8
ZQ	KRTAP5-2
ZQ	KRTAP5-3
ZQ	LCT
ZQ	LCTL
ZQ	LGALS2
ZQ	LGALS7
ZQ	MAOB
ZQ	MB
ZQ	MBL2
ZQ	MTTP
ZQ	NCAN
ZQ	NCOA1
ZQ	NCOA2
ZQ	NFATC1
ZQ	NR3C1
ZQ	NUDT9
ZQ	P2RY12

ZQ	PAH
ZQ	PIK3CA
ZQ	PIK3CG
ZQ	PIK3R1
ZQ	PIM1
ZQ	PLK1
ZQ	PRKACA
ZQ	PRKCA
ZQ	PRKCB
ZQ	PTGIR
ZQ	PTGIS
ZQ	PTGS1
ZQ	PTK2B
ZQ	PYGL
ZQ	PYGM
ZQ	RHO
ZQ	SFTPD
ZQ	SIGLEC1
ZQ	SLC18A1
ZQ	SLC18A2
ZQ	SLC6A2
ZQ	SLC6A3
ZQ	SLC6A4
ZQ	SMARCA5
ZQ	SOAT1
ZQ	SOAT2
ZQ	SQLE
ZQ	STK17B
ZQ	SYK
ZQ	TNF
ZQ	TOP2A
ZQ	TOP2B
ZQ	UGT3A1
ZQ	AGLA
ZQ	ALSB
ZQ	AMS
ZQ	AMY
ZQ	AMYA
ZQ	AMYE
ZQ	AMYM
ZQ	AMYP
ZQ	ASPA
ZQ	ASST
ZQ	BLA
ZQ	BOTB
ZQ	BUDC
ZQ	CBHA
ZQ	CCRA
ZQ	CEL5A
ZQ	CELA
ZQ	CELA1

ZQ	CELB
ZQ	CELC307
ZQ	CELCCF
ZQ	CELCCG
ZQ	CELD
ZQ	CELS
ZQ	CENC
ZQ	CEX
ZQ	CGT
ZQ	CSLB
ZQ	CTXB
ZQ	DMSA
ZQ	ECO
ZQ	ENGF
ZQ	ENTB
ZQ	FABI
ZQ	FABZ
ZQ	FHUA
ZQ	GALM
ZQ	GANB
ZQ	GDHB
ZQ	GFO
ZQ	GLK
ZQ	LACZ
ZQ	LAMB
ZQ	LECB
ZQ	LGTC
ZQ	MALE
ZQ	MALP
ZQ	MGLB
ZQ	MOXC
ZQ	MUTY
ZQ	NEDA
ZQ	NPLT
ZQ	PKN3
ZQ	PPGMK
ZQ	SPOII
ZQ	STX2EB
ZQ	STXB
ZQ	TETX
ZQ	TREZ
ZQ	TTGR
ZQ	TVAI
ZQ	TVAII
ZQ	XLNA
ZQ	XYLA
ZQ	XYNA

Table S3 Detailed information of drug target-disease gene network

Node1	Node2
EPRS	KARS
JUN	FOS
NCOA2	ESR1
SUCLG1	SUCLA2
PTPN1	SRC
TLR4	LY96
PRKAA1	PRKAG1
NFKB1	NFKBIA
MT-CO2	COX5A
JUN	ATF3
SRC	RAC1
LARS	DARS
ABCC8	KCNJ11
ACTA1	UBC
GLDC	GCSH
ATP5A1	ATP5B
EPRS	DARS
MED1	POLR2E
MED1	ESR1
SDHD	SDHC
UBC	NFKBIA
NCF2	CYBA
GRIN1	GRIN2A
MED1	PPARG
AR	UBC
NCOA2	AR
UBC	HMGCR
IKBKB	NFKB1
PRKAG2	PRKAB1
CTNNB1	RUVBL1
MT-CO2	COX7C
SDHB	SDHA
MT-CO2	COX4I1
ASL	ASS1
COX5A	COX4I1
JDP2	ATF2
PRKAG1	PRKAB1
NFKB2	UBC
SRC	PGR
F10	SERPINC1
MT-CO2	COX6B1
NCF4	NCF2
NCOA1	RARA
PPP3CA	PPP3R1
NCOA1	ESR1
SERPINC1	F9
SRC	PIK3R1
CAD	DHODH
GCLM	GCLC
PIK3R1	PIK3CA
AR	CTNNB1
NCF2	RAC1
PRKAA1	PRKAB1
NCF1	CYBA
SUCLG2	SUCLG1
JUN	CSNK2A1
UBC	CTNNB1
ATP5C1	ATP5A1
HDAC2	TP53
BCL2	TP53
ATP5C1	ATP5B
CYTH2	ARF1
IARS	DARS
UBC	HSPA5
SRC	ESR1
IKBKB	UBC
ATF3	ATF4
CYTH2	ARF6
MT-CO3	MT-CO2
HDAC2	SMARCA5
IARS	EPRS

LARS	KARS
UBC	ESR1
MT-CO2	COX5B
AR	SRC
NCOA1	PPARG
HBB	HBA1
MT-CO2	COX6C
GRIN1	GRIN2B
NR0B1	NR5A1
UBC	ALDOA
COX5A	COX5B
VDR	MED1
PAICS	PFAS
SERPINC1	F2
TP53	PIN1
IARS	KARS
VDR	NCOA1
NCF2	RAC2
FARSA	FARSB
IKBKB	NFKBIA
RAC1	UBC
SDHD	SDHB
PRKAA1	PRKAG2
MT-CO3	MT-CO1
MT-CO2	MT-CO1
UBC	CFTR
PPP2CA	UBC
NCOA1	NR3C1
UBC	TP53
JUN	JDP2
SDHC	SDHA
PRKAA2	PRKAG1
PRKAA2	PRKAB1
RABGGTB	RABGGTA
ESR2	NCOA1
LARS	EPRS
JUN	ATF2
SDHB	SDHC
B2M	HLA-B
NCF2	NCF1
JUN	UBC
AR	NCOA1
LARS	IARS
ESR2	ESR1
VDR	NCOA2
PAICS	PPAT
MT-CO1	COX4I1
KARS	DARS
ATF3	TP53
HDAC2	CSNK2A1
THBD	F2
SDHD	SDHA
NR0B1	AR
ARF1	PLD2
GNAS	PTGIR
SUCLA2	OGDH
NCOA2	NCOA1
GNAS	UBC
UBC	PLK1
GARS	IARS
COX5A	COX6B1
COX5A	COX7C
MT-CO1	COX5A
NCOA2	NR3C1
GARS	AARS
LARS	LARS2
GPI	UBC
TOP2A	TOP1
COX5B	COX7C
NCOA2	PPARG
YARS	EPRS
SUCLG2	OGDH
LARS	AARS
JUN	ESR1
NCOA1	PPARA

ABL1	CTNNB1
NCOA2	ESR2
ALDH5A1	ABAT
ALDH18A1	PYCR1
SRC	PTK2B
ASS1	OTC
TOP2A	TOP2B
UBC	ADRB2
PPIA	UBC
COX7C	COX6B1
UBC	PPP2CB
MT-CO3	COX5A
YARS	UBC
JUN	NR3C1
NCF4	CYBA
PTGS2	TP53
ATF3	ATF2
EPRS	UBC
CTNNB1	PIK3R1
IKBKB	NFKB2
COX5B	COX4I1
NOS3	ESR1
TOP2B	TOP1
TP53	CSNK2A1
UBC	ATP5A1
NCOA2	RARA
JUN	TP53
SPTLC1	SPTLC2
ATP5C1	ATP5E
RHO	RCVRN
CTH	CBS
COX5B	COX6B1
PPIB	LEPRE1
ATF6	HSPA5
PPIA	PPP3CA
MED1	RARA
ATP5A1	ATP5E
SRC	ESR2
PRKAG3	PRKAB1
UBC	NR3C1
COX6C	COX5B
NAGS	ASS1
RXRG	RARA
ATP5B	ATP5E
MED1	PPARA
ABCC8	KCNJ8
GLDC	SHMT1
PPIA	PPP3R1
SRC	CTNNB1
CTNNB1	HNF4A
GPI	PGM1
UBC	YWHAE
JUN	ATF4
PSAT1	PHGDH
FOS	TP53
OXCT2	OXCT1
CAD	ASS1
GMPS	APRT
NR3C1	NFKB1
PFAS	PPAT
CACNB3	CACNA1C
TP53	MLH1
SRC	UBC
PPP2CA	LCMT1
GLDC	SHMT2
RAC1	ARF6
UBC	NFKB1
ABCA1	PPARA
SHMT1	TYMS
UBC	ACTG2
HNF4A	HNF1A
VDR	SRC
RPS6KA3	PDPK1
COX6A2	COX5B
YARS	FARS2

UBC	NOS2
IARS	AARS
SRC	GRIN2A
RAC1	PLD1
TOP2A	UBC
SYK	UBC
MT-CO1	COX5B
PRKCA	RAC1
RAC1	NFKB1
UBC	NOS1
UBC	UNG
PTPN1	INS
SRC	NFKBIA
RAC1	NME1
PRKAA2	ACACB
OAZ1	ODC1
PHGDH	UBC
P5CR2	ALDH18A1
UBC	PPARG
CTNNB1	HNF1A
ABL1	SRC
PRKAG1	PRKAG2
PIK3R1	ESR1
CALM1	NOS3
HNF4A	NR0B2
OAT	OTC
JUN	NOS3
HDAC2	UBC
ABL1	JUN
ALDH18A1	PYCRL
GARS	EPRS
CAD	PPAT
CPT1A	PPARA
ESRRG	NR0B2
SLC3A1	SLC7A9
PPARG	ESR1
JUN	IL2
DPYD	CAD
FOS	IL2
ESR2	MED1
COX5A	COX6C
UBC	GLUL
UBC	SQLE
ATF4	ASNS
SUCLA2	SDHA
MAF	FOS
GMPS	CAD
CACNB1	CACNA1D
PRKAA1	PRKAG3
JUN	CTNNB1
IKBKB	PRKCB
COX6C	COX6B1
NFKB2	NFKB1
AR	CDK6
FXN	NFS1
RAC1	CYBA
MT-CO3	COX6B1
FOS	ATF2
RAC1	CTNNB1
ATF6	ATF4
PRKCA	RARA
PRKCA	NCF1
COX4I1	COX6B1
ALDH18A1	OAT
TLR2	NFKB1
FOS	ESR1
PGM1	PYGL
HSD3B1	HSD17B6
SRC	F2
PIM1	NFATC1
GNAS	ADCY2
SUCLG2	SDHA
PIK3CA	ESR1
GMPS	PFAS
COX4I1	COX7C

GCLC	GSS
PSAT1	UBC
UBC	ACSL4
UBC	ATP1A1
S100B	TP53
PGR	NCOA1
GMPS	PRKAG2
CYP17A1	HSD3B1
NCF4	NCF1
JUN	PTGS2
INS	GCK
PRKCA	PLD1
FARSB	AARS
PLK1	TP53
MT-CO3	COX4I1
UBC	CHMP4B
MT-CO1	COX7C
LSS	UBC
HDAC2	NFKB1
PARS2	EPRS
IARS	UBC
SERPINC1	PROC
SUCLG1	SDHA
GARS	ASNS
ESR2	NOS3
AR	TP53
FARSB	FARS2
MTR	MTRR
MT-CO3	COX5B
CSNK2A1	NFKBIA
PTK2B	GRIN2A
TH	DDC
ADSS	CAD
F7	GGCX
PRKAA2	PRKAG2
AGXT	AGXT2
ACACB	CAD
ATF4	FOS
ABCC9	KCNJ8
JUN	BCL2
UBC	TARS
IARS	FARSB
UBC	SPTLC1
ABL1	TP53
PLA2G2A	PTGS2
UBC	RARA
UBC	ANXA1
PFAS	CAD
ATF2	INS
PPP1CC	PLK1
MT-CO1	COX6B1
CACNB2	CACNA1C
NR5A1	NR0B2
CACNA2D1	CACNA1D
ARF6	NME1
COX6C	COX4I1
NFATC1	IL2
PLD2	RAC2
GAD1	ABAT
ATF4	HSPA5
JUN	RAC1
YARS	YARS2
FADS1	PPARA
OAT	ARG2
SRC	PIK3CA
SYK	SRC
CTPS1	UBC
CACNB2	CACNA1D
OAZ3	ODC1
UBC	NME1
ABL1	PIK3R1
LSS	SQLE
NR0B2	ESR1
BGLAP	ATF4
CTNNB1	NFKB1

HSD17B6	CYP19A1
SRC	RARA
GCLM	GSS
ALAD	ALAS1
GGCX	F9
RUVBL1	SMARCA5
JUN	ATF1
HSD3B1	CYP19A1
AR	JUN
ADSSL1	CAD
GLUL	GLUD1
COX6C	COX7C
NAGS	OTC
HNF4A	MED1
ABAT	GAD2
FKBP1A	PPP3R1
NR1H4	MED1
MT-CO3	COX7C
BGLAP	PTH
PGR	ESR1
NOS3	ARG2
PPP3CA	FKBP1A
PRKAA2	PRKAG3
PPARG	IL2
GRIN1	GRIN3A
DRD2	SLC6A3
ACTA1	RAC1
UBC	SHMT1
PPARA	NFKBIA
CYBB	NCF2
PAICS	UBC
ABL1	FOS
SYK	PIK3R1
GMPS	UBC
GRM8	NPY
AARS	ASNS
ALAS1	INS
OAZ1	OAZ3
B2M	JUN
UBC	PYGL
KCNJ11	ABCC9
LY96	TLR2
SUCLG1	OGDH
GARS	DARS
GLS	GLUL
GPI	GCK
UBC	PFAS
NFATC1	PPP3R1
GPI	HK1
PTGS2	PLA2G1B
SYK	TLR4
NAGS	CAD
KCNQ3	KCNQ2
CYP17A1	HSD17B6
ESR2	FOS
OAT	ARG1
HCK	ABL1
DARS	AARS
MT-CO1	COX6C
NR0B2	RARA
UBC	ATF4
UBC	C22orf28
ARG1	OTC
PRKCA	UBC
PPP3CA	NFATC1
THBD	PROC
TGFBR2	UBC
SUCLG1	SDHB
HTR1B	HTR1A
TP53	PRKAB1
FKBP1A	ITPR1
COX6A2	COX7C
KCNJ11	KCNJ8
PPP2CA	PIN1
CBS	BHMT

OPRM1	OPRD1
TLR4	SRC
OAZ2	ODC1
MAOB	COMT
WARS	WARS2
GAMT	GATM
MED1	SULT2A1
NFATC1	PPARG
SRD5A1	SRD5A3
ADCY5	PRKACA
ADSS	ASS1
GPI	ALDOA
SRC	GRIN2B
IARS	ASNS
CACNB4	CACNA1A
ALAD	ALAS2
NOS1	GRIN2A
BCL2	ITPR1
PTGS1	PTGIS
SRC	FOS
SUCLA2	SDHC
TARS	SARS
PDPK1	PIK3CA
PRKCB	NCF1
NR0B1	ESR1
PIK3CG	PIK3R1
RPS6KA3	ATF4
MT-CO3	COX6C
UBC	CDK6
CYBB	CYBA
PTGS2	PTGIS
TOP1	TP53
UBC	ARF6
SUCLA2	SDHB
UBC	PPP1CC
NR0B1	ESR2
PLD1	ARF6
ATF4	ATF2
PSAP	GBA
PROC	GGCX
SCN5A	SCN2B
DCTD	TYMS
GOT1	CAD
HAO1	AGXT
AR	MED1
CAD	GOT2
HDAC9	TP53
TOP1	UBC
COMT	DDC
FOS	CSNK2A1
SRC	PDPK1
HARS	TARS
UBC	GLUD1
FOS	NFKB1
GRIN1	GRIN2D
ARF6	PLD2
TNF	PTGS2
TH	FOS
PDPK1	PRKCB
PRKAA1	ACACB
CACNA1B	CACNB3
NR5A1	CYP19A1
GMPS	PRKAG3
GMPS	PAICS
GMPS	PRKAG1
UBC	ARF1
NOS1	GRIN2B
NR1I2	NCOA1
PPARG	NFKB1
UBC	SDHA
ALDOA	GAPDHS
ACOX1	PPARA
TNF	NFKB2
SHMT2	GCSH
PRKAA2	PRKAA1

ACOX1	CPT1A
HNF4A	GCK
NCF4	RAC1
CALM1	CSNK2A1
UBC	CSNK2A1
PPARG	INS
TARS2	FARS2
ADCY5	GNAS
PRKACA	NFKB1
GMPS	CTPS1
HK1	UBC
PIK3CG	PDPK1
PPP2CA	BCL2
PPARA	INS
CACNA2D1	CACNB3
SLC6A3	DRD4
TGFBR2	CTNNB1
HDAC9	JUN
GNAS	ADRB2
VDR	TP53
RXRG	RARB
SOD2	CAT
CALM1	CACNA1C
UBC	SLC25A4
FARS2	TARS
GRIN2A	GRIN2B
CACNB1	CACNA1S
UBC	SHMT2
CACNB1	CACNA2D1
GPI	TPI1
GCSH	SHMT1
EPRS	CAD
CRH	NPY
GPI	GNPDA1
CACNB1	CACNA1C
SUCLG2	SDHC
PRKCA	PLD2
HSD17B1	CYP19A1
PRKCA	NFKBIA
MTR	CBS
BCL2	PIN1
TP53	ESR1
RAC1	ARF1
SRC	NCF1
NCOA2	MED1
ADH1B	ALDH1A1
SLC25A4	PPIF
PTPN1	CTNNB1
HNF4G	HNF1A
PROS1	PROC
CYP1A2	GSTM1
SRC	NFKB1
IARS2	FARS2
UBC	ATP5B
CALM1	NOS2
COX5A	COX6A2
GLUL	CAD
JUN	SRC
CBS	SHMT1
CAD	GAD2
ACACB	PRKAG2
SCN3B	SCN2B
SHMT2	TYMS
COX6A2	COX6B1
GNAS	PTH
MT-CO2	NOS2
ALDH18A1	NAGS
SYK	PIK3CG
GAD1	CAD
PPARG	NR0B2
CTSD	ESR1
SUCLG2	SDHB
ARG1	ODC1
UBC	ANXA5
PTGS1	PLA2G1B

CTNNB1	PPARD
ESR2	NR0B2
SRC	ADRB2
SRD5A1	AKR1D1
HDAC2	FOS
BCL2	PPIF
AR	ESR1
RAC1	PIK3CA
TLR4	NFKB1
YARS	FARSA
PAH	TAT
PTDSS1	PISD
CACNA2D1	CACNB2
SCN4B	SCN3B
CTNNB1	PIK3CA
UBC	TYMS
RAC1	NCF1
AR	NR0B2
UBC	AKR1B1
RXRA	RARA
CALM1	ITPR1
PIK3CG	SRC
SRC	NOS2
PRKAG2	PRKAB2
ARG1	ASL
ARG1	NOS1
GRIN3A	GRIN2B
SRD5A1	CYP19A1
TP53	NR0B2
PRKCA	SRC
HNF4A	TP53
GRIN3A	GRIN2A
NCOA1	RORA
RXRA	PPARG
SLC25A18	GUCA1A
SUCLG1	SDHC
GLDC	AGXT
GAD2	SLC32A1
CACNB3	CACNA1D
RAC1	PIK3R1
LARS	UBC
PIK3R1	EPOR
NR1I3	CYP1A2
ALDH18A1	CAD
IKBKB	PPP2CA
F2	PROC
AR	HNF4A
VDR	HNF4A
ARG2	OTC
ACTA1	SRC
ADCY5	ADCY2
RUVBL1	POLR2E
PTPN1	CSNK2A1
MTR	SHMT1
HNF4A	ESR1
RAC1	BCL2
SRC	EPOR
GNAS	ADRB3
HK1	PGM1
F7	F9
CACNA1S	CACNB2
SYK	JUN
SHMT1	AGXT
TOP2A	CSNK2A1
BCL2	NFKB1
CAD	ASNS
ASL	NAGS
CACNB4	CACNA2D1
MAOB	DDC
INS	NPY
PRKAA1	CFTR
TP53	NFKB1
F10	GGCX
F2	GGCX
F7	F10

NCOA2	CTNNB1
TH	TYR
NCOA2	RORA
RARG	RXRG
ASL	OTC
SYK	PTK2B
CYP17A1	SRD5A1
CACNA1F	CACNA2D1
XDH	CAT
PDPK1	INS
OAT	ODC1
VDR	RXRA
APRT	UBC
PKIA	PRKACA
ESR2	PPARG
F2	F9
RAC1	NOS2
TAT	GOT2
HDAC2	HDAC9
JUN	PPARA
POLA1	TYMS
AR	NR3C1
RXRA	NR1I2
CDA	UCKL1
NR1H4	ABCB11
GSTP1	CYP1A2
AR	NR5A1
SLC7A1	UBC
SMPD4	SMPD3
NR1I2	CYP2B6
SHMT2	AGXT
CACNB4	CACNA1S
HTR7	CRH
CAD	OTC
UBC	PRKCB
PRKCA	NOS1
PRKACA	CFTR
ABCC8	INS
IGF2R	M6PR
CBS	SHMT2
FARS2	WARS2
CACNB1	CACNG1
JUN	INS
COMT	SLC6A4
HAO1	CAT
SLC25A15	ASS1
FOS	ATF1
GLUD1	GOT2
RAC1	TLR2
NOS3	INS
UBC	AARS
SRC	INS
SRC	FHIT
GLUD2	GLUL
CTSB	CTSD
NFATC1	FOS
PROS1	F2
RARA	ESR1
GAD2	INS
FOS	NR3C1
RXRG	PPARG
HSPG2	SLAMF7
ACSL3	UBC
RAC1	PTK2B
HSD3B1	CYP11B2
PROS1	GGCX
RXRA	NR1I3
YARS	TARS2
HDAC2	JUN
UBC	FOS
KCNJ1	CFTR
PGM1	GCK
SRC	PLD2
VDR	RXRG
SYK	RAC1

UBC	FARSA
CACNA2D1	CACNG1
EPRS	ASNS
CTNNB1	YWHAE
CHRNA4	CHRNB2
HSD11B1	AKR1D1
JUN	NFKBIA
PIK3R1	NFKBIA
ARG2	ODC1
CHRNA2	CHRNB2
F2	PLAT
TNF	HSPA5
CHRNA3	CHRNB4
ADRB3	ADRB2
ASS1	SLC25A2
GRIA1	GRIA2
RARA	NR3C1
IKBKB	PRKCA
PIK3CG	FOS
OAZ1	OAZ2
HTR1A	HTR1D
CHRNA4	CHRNB4
MED1	RORA
HSD3B1	SRD5A1
ABL1	RAC1
CYP17A1	ESR1
CACNA2D1	CACNA1C
CACNA1S	CACNA2D1
NAT1	CYP1A2
ADRA1A	ADRA1B
OPRK1	OPRD1
PRKACA	NR3C1
NR1I2	ABCB11
CACNB4	CACNA1D
RXRG	NR0B2
CHRNA2	CHRNB4
SIGMAR1	SQLE
TP53	CTSD
HSD17B6	SRD5A1
PIK3CA	RAC2
PTGS2	FOS
B2M	LYZ
CCBL1	KYNU
HMGCR	PPARA
YARS	TARS
HTR1B	HTR1D
CACNB4	CACNA1C
IKBKB	TLR4
CYP11B2	HSD17B6
AR	NFKB1
HDAC2	CTNNB1
SHMT2	SDS
UBC	MIF
KYNU	AADAT
ADRA1A	ADRA1D
SHMT1	SDS
HDAC2	ISYNA1
CTH	MTR
UBC	DARS
SRC	ARF6
HSD3B1	HSD11B1
HCK	SRC
ASS1	ASNS
AMY1A	PYGL
CYP2B6	GSTM1
MTR	SHMT2
UBC	SCARB1
GMPS	ADSS
GSTM1	CYP2A6
CXCL10	CXCR1
PAH	DDC
JUN	NFATC1
MT-CO3	COX6A2
PRKAG1	PRKAB2
GSTM1	ADH1B

HTR1A	NPY
IARS	FARS2
ABCA1	SPTLC1
HSD17B6	AKR1D1
CARS	EPRS
ARG1	NOS2
AMY2B	PYGL
AMY2A	PYGL
SUCLG1	OXCT1
FOS	NPY
FOS	CRH
GLDC	AGXT2
ADCY2	PRKACA
CTNNB1	FOS
RPS6KA3	UBC
CACNA1S	CACNB3
ACTA1	PIK3CA
AOX1	ADH1B
ALDH18A1	UBC
ALDH18A1	GLUD1
ALDH18A1	GLUD2
NCOA1	MED1
GSTP1	CYP2B6
ODC1	OTC
YARS2	FARS2
RAC2	NFKB1
VDR	NR0B2
UBC	POLR2E
TH	JUN
TP53	NR3C1
GLS	GLUD1
PGR	JDP2
TRPM8	TRPA1
NCOA2	PPARA
TNF	BCL2
UBC	GSS
ADCY5	PRKCA
INS	ESR1
CYBB	NCF1
PTH	CRH
UBC	SLC7A11
CACNA1D	CACNG1
PCTP	ACOT13
PTH	CYP27B1
PROC	PROCR
FOS	INS
MGMT	TP53
SYK	IL2
PRKACA	YWHAE
F2	FURIN
GSTP1	GSS
CACNA1F	CACNB2
COX8A	COX7C
ABL1	GRIN2D
F2	PPARA
CTH	BHMT
PIK3R1	INS
DPYD	CYP2A6
B2M	IL2
NFKB2	NFKBIA
SYK	NFKB1
IKBKB	TLR2
TP53	PIK3CA
FARSA	FARS2
HTR2A	GRM1
UBC	ASNS
CACNB4	CACNA1F
CMPK2	NME1
SHMT2	MTFMT
SRC	NOS3
PIK3CA	INS
NR1H4	NR0B2
UBC	PPIH
GRIA2	GRIA3
FARS2	LARS2

LARS	TARS
PRKCA	BCL2
HSPA5	PPIB
MT-ND1	MT-CO1
RPS6KA3	FOS
ALDH18A1	PYCR2
ADSS	PFAS
GRIN1	GRIN2C
PRKCA	PIK3CA
GAD1	GLS
NR0B1	CYP19A1
GARS	UBC
GARS	SARS
XDH	AOX1
SYK	FOS
TP53	PPIF
PRKCA	PDPK1
DGKA	PLD2
HTR1B	NPY
CACNA1F	CACNB3
PTGS2	NFKB1
CYP17A1	CYP19A1
COX6A2	COX4I1
JUN	NCOA1
FURIN	PROC
GLS	CAD
TNF	NFKB1
PTDSS2	PISD
ACACB	CPT1A
GLS	GAD2
PGR	MED1
GSTP1	CYP2A6
ADCY5	ADRA2A
SYK	RAC2
PTGS2	MT-CO2
CTSA	CTSD
GNMT	SHMT1
PDPK1	PIK3R1
FOS	HNF1A
IKBKB	SRC
PAICS	CAD
ACSL3	CPT1A
UBC	FADS1
PRKAA1	PRKAB2
PPARA	NR0B2
CA9	CTNNB1
TNF	IL2
PROS1	SERPINC1
UBC	KARS
FURIN	F9
HSD17B6	SULT1E1
EPRS	CARS2
SHMT2	PFAS
GRM8	CHRM2
MT-CO1	COX6A2
MED1	TP53
PTGS2	BCL2
HNF1A	INS
GSTP1	ADH1B
ARG1	NOS3
PPARA	SCARB1
EPRS	AARS
PRKAB2	PRKAG3
CACNA1G	CACNB2
SOAT1	UBC
ACSL4	CPT1A
PPP3CA	UBC
SRC	NR0B2
PTPN1	PIK3CA
CTH	CDO1
GAD1	GLUL
HTR3A	HTR7
NR1H4	FABP6
CTPS1	CAD
BCL2	ATF2

SHMT2	FTCD
CACNB1	CACNA1G
ACTA1	PLD1
ADH1B	ALDH1A2
SHMT1	FTCD
PIK3CA	NFKB1
JUN	ACTA1
GSTP1	UBC
HTR2A	FOS
NOS2	ARG2
PTK2B	ITGB2
TRPV1	TRPA1
AGXT	SDS
SRR	SHMT1
CYP2C8	PTGS2
CYP2B6	SULT2A1
ABCA1	PPARG
HSD17B6	ALDH1A1
HCK	ITGB2
GOT1	ASNS
GSTM1	GSS
GLUL	GAD2
IARS	TARS
GPI	GAPDHS
HARS	UBC
GOT2	ASNS
BCL2	IL2
TP53	NME1
GAMT	CKM
UBC	PIK3R1
DDC	TYR
HAO1	DAO
NOS3	ASS1
SUCLA2	ALAS2
HNF4A	F2
CARS	YARS
GLUL	PPAT
BCL2	PGR
UBC	CBS
FPGS	TYMS
PTGS2	CTNNB1
DRD2	NPY
MT-CO2	MT-ND1
UBC	SCN5A
SCN4B	SCN2A
NCOA2	FOS
IKBKB	CTNNB1
PLA2G2A	PTGS1
TRAPPC3	UBC
GNAS	ADRB1
CTH	CCBL1
ATP2A1	FURIN
UBC	FARSB
NCOA2	JUN
NOS2	NFKBIA
GPT2	GOT2
EXT1	HSPG2
GOT1	GPT2
COX8A	COX5A
CACNA1I	CACNA1D
PDPK1	NFKB1
PRKCA	NFKB1
ABL1	CDK6
CALM1	SCN5A
CACNA1I	CACNA1C
TAT	NR3C1
P2RY12	NPY
MTR	FTCD
TH	TAT
F2	CFTR
CTH	GOT1
SRR	SHMT2
CTPS1	NME1
TRPV1	KCNQ2
CACNA1I	CACNA1S

PPP2CA	YWHAE
ALDH18A1	ASS1
GRM4	HTR1A
RPS6KA3	ATF1
YARS	WARS2
PTPN1	EPOR
GRM7	HTR1A
SHMT1	MTFMT
MAF	SMARCA5
JUN	NOS2
CYP2C8	GSTM1
MT-CO2	PTGS1
GPT	GOT1
SUCLA2	SDHD
UBC	BCL2
ACACB	PRKAG3
CTNNB1	ACP1
WARS	UBC
OPRM1	NPY
SCN1B	SCN2B
CTH	GOT2
PPP2CA	RPS6KA3
PPARA	SULT2A1
GRIN1	NOS1
JUN	ACHE
CYP19A1	SULT1E1
CACNA1G	CACNA1C
FKBP1A	NFATC1
GAD1	SLC32A1
PROS1	SERPINA1
PPP3CA	NOS1
ADCY2	DRD1
PPARG	HSD11B1
HTR7	PTGIR
TNF	TP53
CYP17A1	CYP11B2
NFKB1	EPOR
PRKCB	PPP2CB
NCOA1	FOS
OAT	UBC
UBC	SIGMAR1
CALM1	UBC
ITPR1	INS
ATF2	ESR1
PLD1	PISD
TARS2	SARS
TRPV3	TRPM8
CTNNB1	CSNK2A1
CACNA1H	CACNA1C
PRKCB	NFKBIA
AR	PIK3CA
GRIK2	GRIK5
CACNA1G	CACNA1D
LGALS7	TP53
AGXT	DAO
ABL1	NFKBIA
L3HYPDH	DAO
PGM1	ALDOA
YARS	FARSB
PIK3CG	RAC1
OPRM1	DRD4
CACNA1S	CACNA1G
SRR	SDS
TNF	SRC
KYNU	CCBL2
NOS2	PPARA
PPARD	MED1
CACNA1H	CACNA1D
NOS2	ASS1
CCBL1	AADAT
MGAM	AMY2A
NCF1	RAC2
HNF4G	NR0B2
ITGB2	NFKB1
CARS	AARS

GLDC	GNMT
HTR1D	NPY
GSTP1	CYP2C8
SOD2	UBC
GNMT	SHMT2
F2	ESR1
HSD17B6	ALDH1A2
RXRG	PPARA
CYP11B2	HSD11B1
AR	FOS
PRKCA	ATF2
CYP17A1	SRD5A3
DGKG	PLD2
RAB9A	M6PR
OPRK1	NPY
ACACB	PRKAG1
SDHB	OGDH
TP53	CAT
GSTP1	TP53
ASL	CAD
COMT	UBC
COMT	ADH1B
PPP1CC	INS
PLD1	DGKG
PAPSS1	MTRR
PISD	PLD2
HMGCR	INS
TOP2B	UBC
GRIN1	CALM1
SUCLG1	SDHD
PLD1	DGKA
VDR	ESR1
GLDC	DAO
APH1A	PSENEN
JUN	ARG1
HTR7	PTH
HPGDS	PTGIS
CYP2C8	CYP1A2
GNAS	ESR1
DRD1	CALY
BCHE	INS
CACNB4	CACNA1H
HK1	ALDOA
CACNB1	CACNA1H
CACNA1I	CACNB2
BCAT2	GLUD1
TARS2	FARSB
JUN	HNF4A
SCN1B	SCN2A
FXN	ACO2
CYP1A2	CYP2B6
ADRB2	CFTR
MT-CO2	PPARA
ADH1B	ALDH2
HSD3B1	AKR1D1
RAC1	NOS3
IL2	NFKB1
CACNB4	CACNA1G
VDR	AR
NFKB1	ESR1
SYK	PIK3CA
CYP1A2	CYP2A6
HNF4A	PPARA
UBC	PSENEN
F10	FURIN
SCN1B	SCN4B
PRKACA	ITPR1
PIK3R1	NFKB1
CBS	SDS
HLA-B	UBC
MED1	NR0B2
GLS	PPAT
PAH	GOT1
PROZ	GGCX
MTAP	UBC

RORA	NR0B2
CACNA1G	CACNB3
COX8A	COX5B
ADRA2C	ADRA2A
DRD2	GRM7
CTNNB1	BCL2
TRPV1	SRC
RARG	MED1
PGR	NR0B2
SCN5A	SCN4B
P2RY12	ADRA2A
GCAT	GLDC
CACNA1S	CACNA1H
COX8A	COX6C
PLK1	PIN1
PPARA	ESR1
PIK3CA	NFKBIA
PPP2CA	PPP3R1
ALAS1	PPARA
EPRS	ACACB
HSD3B1	CYP1A2
GRM1	HTR2B
F7	FURIN
UBC	PRKACA
GALM	GCK
ADCY5	HTR7
RARB	RARA
SUCLG1	ACACB
CYP2B6	ALDH1A1
FECH	FXN
SUCLG2	SDHD
CYP11B2	CYP19A1
SYK	NFKBIA
MTR	UBC
ADCY2	HTR7
PGM1	UBC
ABCA1	UBC
PTGS2	NFATC1
ADCY5	DRD2
PTK2B	IL2
DRD2	GRM8
NOS1	OTC
HDAC9	NOS3
UBC	GPRC5A
PTGS2	CYP2B6
MTTP	INS
RUVBL1	CAD
IARS	YARS
PPARG	TP53
YARS2	WARS2
ADCY5	DRD1
DPP4	CXCL10
PAH	GOT2
NOS1	ASS1
CALM1	PPP3R1
PPP3R1	PPP2CB
SRC	HNF1A
ADSSL1	ASS1
RXRG	MED1
FOS	PTH
UBC	METAP2
NOS2	OTC
FARSB	TARS
SDHA	COX4I1
PRKCA	YWHAE
ASPA	FOLH1
CBS	AGXT
CHRM2	NPY
VDR	PGR
COX8A	COX4I1
KARS	TARS
HNF4A	PPARG
SCN4B	SCN2B
OPRM1	DRD2
ESR2	PGR

YARS	AARS
TRPV1	TRPM8
IARS	TARS2
SYK	ITPR1
GNAS	F2
OPRM1	GRM8
GOT1	CDO1
HCK	SYK
JUN	F2
ATF2	IL2
SRC	ADRB3
HCK	PTK2B
CACNA1A	RAC1
OPRD1	DRD4
IFNB1	JUN
CACNA1H	CACNB2
CACNA1I	CACNB1
NQO1	ODC1
TAT	TYR
PRKACA	CACNA1C
ADCY5	ADRB2
PPARD	NR0B2
CACNB4	CACNA1I
GRM7	CHRM2
GRM8	HTR1A
AR	PIK3R1
DCTD	CMPK2
ADCY5	ADRB1
AR	ESR2
LARS	FARS2
CTH	CCBL2
CYP11B2	SRD5A1
NPY	DRD4
TH	GOT1
NOS3	OTC
GSTP1	GSTM1
TH	GOT2
CA9	TP53
SRR	AGXT
ACTA1	FOS
GOT1	GLUD1
PTPN1	PLD2
SLC25A12	GOT2
GRM4	NPY
SUCLG2	ACACB
ADCY2	DRD5
UBC	COX6B1
TLR2	CYP27B1
AR	PPARA
SCN5A	SCN3B
HCK	F2
NR1H4	RXRG
NR5A1	JUN
GALM	LCT
COX8A	COX6B1
PTGS2	HPGDS
AR	PPARG
ADRB2	PTH
GABRB2	GABRA1
YARS	IARS2
CYP17A1	INS
UBC	SARS
MTR	IL4I1
ABCA1	MED1
GRM8	OPRD1
CHRNA1	CHRND
TH	ATF2
AOX1	NNMT
GRM4	DRD2
ANXA1	NR3C1
ARF1	PIK3CA
GRM4	HTR1B
CYP1A2	ALDH1A1
IFNB1	NR3C1
ARF6	ARF1

NPY	OPRD1
GMPS	EPRS
CACNB1	CACNA1A
ABCA1	RXRG
PPIA	PIN1
CYP17A1	CYP1A2
GNAS	HTR7
LCT	GCK
CDO1	GOT2
NCF2	ARF1
EDNRA	ANXA1
UBC	PLD2
GAD1	GAD2
VDR	PPARA
YWHAE	NFKB1
CYP2C8	ALDH1A1
NOS2	ATF2
GNAS	CRH
MAOB	ADH1B
GLDC	PIPOX
B2M	HSPG2
NAT1	CYP2A6
PVR	SRC
PTGS2	UBC
GATM	AGXT2
GRM4	CHRM2
AOX1	HSD17B6
PGM1	TPI1
JUN	MT-CO2
UBC	LYZ
SUCLA2	ACACB
NCF4	RAC2
RAC1	SH3RF1
ADCY5	DRD5
GATM	AGXT
GABRB1	GABRA1
GRM7	NPY
PTPN1	PIK3R1
RXRG	RORA
DPYD	SDHA
HCAR2	SUCNR1
UBC	EPOR
HSD17B1	SULT1E1
GLO1	AKR1B1
PIK3CG	RAC2
NR5A1	RORA
MT-CO1	COX8A
PIPOX	DAO
F2	TUB
CTNNB1	PSENEN
PGR	PPARA
GLUD2	GAD2
CTNNB1	PROC
PRKACA	NOS3
PPP3CA	PPP1CC
PLA2G2A	PLD2
POLR2E	NME1
HSPG2	LYZ
GAD1	GLUD2
PRKACA	GRIA1
NOS1	NOS2
PIK3R1	IL2
NFATC1	MAF
PTDSS1	PTDSS2
GABRA5	GABRB2
PAICS	SHMT2
GRM7	DRD4
GRIK2	GRIA1
GRM7	OPRD1
JUN	FHIT
ASPA	ACY3
IFNB1	PTPN1
FKBP1A	TGFBR2
HTR1B	GRM7
PGR	HNF4A

IFNB1	FOS
DRD2	KCNJ5
DRD1	PTH
CACNA1I	CACNB3
GNAS	ADRA1B
GABRA5	GABRB3
PTK2B	GRIA1
CTNNB1	MED1
HCAR3	SUCNR1
CACNA1H	CACNB3
SMPD4	UGT8
ALDOA	GCK
SLC25A12	GOT1
GNAS	DRD1
HDC	HAL
PRKCA	GNAS
SLC25A13	GOT1
HCK	PIK3CG
CACNA1I	CACNA1G
PTGS2	PPARA
AR	PGR
PDPK1	NFKBIA
UCKL1	CMPK2
DDC	TAT
GABRA2	GABRB1
HNF4A	PPARD
DRD3	GRM8
VDR	ESR2
PROZ	FURIN
GRM4	HTR1D
XDH	CYP1A2
CACNA1G	CTNNB1
CYP19A1	AKR1D1
RPS6KA3	HTR2A
F10	F2
GABRB2	GABRA4
ESRRG	MED1
ADCY5	OPRM1
CYP17A1	AKR1D1
GABRB1	GABRA4
NR5A1	RXRG
SLC25A15	OTC
GABRA3	GABRB2
SLC25A13	GOT2
GABRB2	GABRA6
GABRA5	GABRB1
PIK3CG	PIK3CA
HTR1B	DRD2
UGT8	SMPD3
GPT	GOT2
HNF4G	MED1
GNAS	PRKACA
HTR7	DRD5
TAT	BHMT
ADRB1	ADRB2
HSPG2	NCAN
RARG	NR0B2
VDR	PPARG
UBC	GOT2
ADSS	ASNS
GABRB3	GABRA1
HTR1B	DRD4
AOX1	ABAT
ESR2	RORA
PTGS1	HPGDS
UBC	OPRD1
GABRG2	GABRB2
PROS1	THBD
PLD1	PLA2G1B
GABRG2	GABRA1
NOS1	ARG2
GABRA3	GABRB1
RXRG	PPARD
TLR4	RAC1
CYP2A6	ALDH1A1

GNAS	ESR2
NR3C1	ESR1
PRKCA	PIK3R1
PTGIR	PTH
ESRRG	PPARG
CYP17A1	SULT2B1
TNF	FOS
HSD17B6	SRD5A3
GABRA2	GABRB2
OPRM1	GRM7
MAOB	IL4I1
RARB	NR0B2
PGR	PPARG
GRM4	DRD4
ADCY2	PTH
CHRM2	OPRK1
ESR2	PPARA
ESR2	HNF4A
OPRM1	GRM4
PLA2G2E	PTDSS2
HTR1B	GRM8
GRM8	OPRK1
NCF4	CYBB
OAZ2	OAZ3
GABRB1	GABRA6
SHMT2	AGXT2
GCSH	AGXT
GLDC	ALAS1
ADRA1B	ADRB2
NAGS	GLUD1
HCAR3	DRD3
NR5A1	ESR1
GRIN2A	GRIN2C
HTR7	DRD1
DRD2	OPRK1
DARS2	FARS2
ESRRG	HNF4G
EDNRA	NOS3
CACNB2	PRKACA
HCAR2	DRD3
GRM8	DRD4
HTR7	ADRB2
PPARD	HNF1A
POLA1	UBC
GRIN2B	GRIN2D
HDAC9	AR
GABRA3	GABRB3
MT-CO3	COX8A
AR	RORA
PTK2B	ARF1
NOS3	TP53
OPRM1	DRD3
HNF4G	RARG
TGFBR2	PPP1CC
PPP3CA	CALM1
F7	SERPINC1
GABRG3	GABRB2
OPRM1	HTR1B
HK1	GNPDA1
PTGS1	CYP2B6
SUCLA2	OXCT1
ADRA2A	NPY
ARF6	PIK3CA
PIK3R1	NR3C1
GABRB3	GABRA4
VDR	RARA
MTR	MTFMT
VARS	EPRS
HNF4A	RARA
DRD2	HTR1A
PPARA	RORA
UBC	RUVBL1
DRD2	OPRD1
ACTA1	CTNNB1
NR5A1	RARA

AR	SULT2A1
ADRB1	ADRB3
CHRM2	HTR1D
GRIK2	GRIK1
HK1	GALM
DRD3	DRD4
TLR4	INS
HCAR2	GRM8
F2	HTR2C
CYP2A6	HSD11B1
CHRNA7	CHRNA4
RORA	ESR1
HCAR2	NPY
DRD2	DRD4
NR5A1	ESR2
EDNRA	EGF
PPP1CC	PPP3R1
HTR1D	OPRD1
PRKACA	GRIA4
RORA	RARA
JUN	CPT1A
PGR	RORA
OPRM1	HTR1A
ABCA1	INS
PROS1	FURIN
GNAS	EDNRA
F10	F9
CTPS1	POLR2E
RAB9A	IGF2R
IARS	INS
CHRM2	OPRD1
GCAT	ALAS1
CYP1A2	SULT2B1
LARS	IARS2
NR1I2	CYP2A6
GRIN2A	GRIN2D
UBC	OGDH
PLA2G2A	FADS2
ASPH	JUN
PPARD	ESR1
ESRRG	PPARA
PPP2CA	SRC
CTNNB1	CDK6
PLA2G1B	PLD2
SDHB	ATP5C1
HNF4G	PPARD
GABRG2	GABRA2
DRD3	OPRD1
CHRNA7	CHRNB2
ADRA2C	DRD4
CYP11B2	AKR1D1
HTR1D	DRD4
VDR	HNF4G
ADCY5	CHRM2
GATM	GLDC
ASS1	GOT2
AOX1	CYP1A2
ADCY2	CHRM2
ATP5C1	COX5A
ADCY2	NPY
CYP19A1	ESR1
GRM4	OPRD1
DRD2	CHRM2
ESRRG	RORA
ADRA2A	DRD4
GABRA2	GABRB3
GLUD2	GLS
HARS	KARS
HTR1B	CHRM2
DRD3	NPY
GRM4	OPRK1
PRKCA	GRIN2B
AR	RARA
GABRG3	GABRB3
SRD5A3	CYP19A1

ADRB1	HTR7
DRD3	OPRK1
HNF4A	RORA
HNF4G	PPARG
ASPA	ASS1
PPARG	RORA
GRIN2C	GRIN2B
PTDSS1	PLA2G2E
HNF4G	RARB
GABRG2	GABRB1
OPRM1	ADCY2
PPP1CC	PRKACA
HCAR3	GRM8
VDR	RARG
DRD3	HTR1A
RARB	ESR1
SHMT1	ALAS1
VDR	PPARD
NOS1	PRKCB
ADRA2B	CHRM2
GNMT	AGXT
GABRG2	GABRA5
HTR3A	HTR1A
RARG	RORA
HCAR2	ADRA2A
GABRG2	GABRA3
GABRB3	GABRA6
VDR	RORA
PLA2G2E	PTGS1
NAT1	XDH
ADCY5	ADRA2B
CACNA1I	CACNA1H
HNF4G	RARA
MED1	ACOX1
HNF4G	PPARA
CYP1A2	PPIG
HNF4G	NR5A1
ESRRG	HNF4A
KYNU	CAT
GOT1	ASS1
GABRG2	GABRB3
CACNA1G	CACNA1H
PPP2CA	PPP1CC
ADRB3	CRH
OPRM1	CHRM2
HTR1A	DRD4
UBC	SMARCA5
MT-CO3	MT-ND1
ADCY5	HTR1A
HTR1B	ADRA2A
GMPS	DHODH
HTR3A	FOS
CYP17A1	ESR2
RARG	HNF4A
DRD2	HTR1D
ITGB2	PROC
NR5A1	HNF4A
F2	ADRB2
CYP2B6	ALDH1A2
CHRNA7	CHRNA2
ESR2	RARB
NOS2	RAC2
MT-CO2	COX8A
ADRB3	HTR7
GABRG3	GABRB1
NR5A1	RARB
CBS	SRR
HNF4G	ESR2
CYP2A6	ALDH1A2
NR5A1	PPARA
SHBG	SULT2A1
CHRNA7	CHRNB4
F2	NFKB1
HNF4G	RORA
FPGS	UBC

GCAT	ALAS2
RARB	HNF4A
GRM1	PIK3CA
PPARG	RARA
DRD3	GRM4
DRD2	ADCY2
DRD3	GRM7
CTSB	BCL2
NOS1	GRIN2D
ADRA2C	DRD3
PGR	RARA
ADCY2	HTR1A
GNMT	AGXT2
ARF6	PIK3R1
JUN	ACOX1
GABRG2	GABRA4
ADCY5	GRM4
RARB	RORA
CYP2C8	ALDH1A2
ESR2	RARA
HNF4G	ESR1
PGR	PGRMC1
ERVW-1	ENPEP
HCAR2	GRM7
PPARA	RARA
VDR	NR5A1
GABRA5	GABRG3
GRM8	GRM1
CTNNB1	GRIA2
HCAR2	GRM4
CYP1A2	ALDH1A2
PTGIR	CRH
PIK3CA	IL2
HCAR3	GRM4
HNF4G	RXRG
GRM7	GRM1
VDR	RARB
GMPS	ADSSL1
DRD3	CHRM2
GRM8	HTR1D
CYP2C8	PTGS1
PGM1	PYGM
SUCNR1	P2RY12
UBC	FADS2
RAC1	EDNRA
CHRNA7	CHRNA3
PLA2G2E	FADS2
HCAR3	GRM7
NFKB1	PSENEN
PPP1CC	PPP2CB
NR5A1	PGR
RARG	ESR1
ESRRG	AR
ADCY5	PTH
PPARD	RORA
GABRG2	GABRA6
TLR4	CXCL10
RARG	PPARG
RARB	PGR
ESRRG	PPARD
GRM4	GRM1
OPRM1	HTR1D
HNF4G	AR
GCAT	PIPOX
ADRA1D	HTR2B
RARB	PPARG
HTR1B	OPRD1
ESRRG	ESR1
GATM	DAO
GLRA1	GLRB
RARG	PPARA
IKBKB	LY96
ADCY5	ADRB3
CSAD	CDO1
AKR1B1	LCT

ESRRG	ESR2
HCAR3	OPRK1
ESRRG	PGR
EPRS	LARS2
PIPOX	AGXT
ESR2	PPARD
HCAR3	GPR18
AR	PPARD
ADRA2C	DRD2
GOT1	TAT
HCAR2	OPRK1
ADSSL1	ASNS
HTR1A	OPRK1
PIK3R1	RAC2
HCAR3	ADRA2A
PTDSS2	PLD2
GLUD1	GAD2
MAOB	AOX1
GCAT	SHMT2
DRD2	ADRA2A
GLUD2	NAGS
SRC	TLR2
HTR2A	HTR2B
CHRM2	DRD4
SRC	EGF
DRD3	HTR1B
HTR1A	P2RY12
ADCY2	ANXA1
HNF4G	PGR
PAICS	SHMT1
DRD1	CRH
ADRA1B	HTR2B
ADRB2	HTR2C
IKBKB	PDPK1
NCOA2	ABCA1
SDHA	OGDH
ALDOA	ESR1
YWHAE	PIN1
ADRA2C	GRM4
ADRA1A	HTR2A
ALAS2	AGXT
SCN3B	TP53
SERPINA1	ALDOA
SMPD4	UBC
RARG	PPARD
ADCY5	CRH
ALAD	FECH
RARB	PPARA
PGM1	RBKS
PLA2G2E	PTGS2
ARG1	SLC25A15
OPRK1	DRD4
PGR	PPARD
ESRRG	NR5A1
AKR1B1	GALM
RXRG	HNF4A
HTR1A	ADRA2A
F10	ITGB2
PPARD	RARA
ACTA1	CHRNA1
RXRG	BCL2
ADCY5	PTGIR
UBC	PLD1
ADCY5	NPY
GAD1	GLUD1
SLC25A2	OTC
ALAS1	AGXT
HTR1A	OPRD1
UBC	PRKAG1
PRKCA	PTK2B
NOS2	NR0B2
HTR1B	OPRK1
CYP2C8	CYP2B6
PIK3R1	ARF1
AOX1	CYP2A6

DRD3	HTR1D
SDHB	HSPA5
CHRNG	CHRNB1
ADRA2B	ADRA2A
ADRA2C	CHRM2
RARG	AR
PRKCB	IL2
ADRA2B	DRD2
NR5A1	PPARD
GRM7	ADCY2
NR5A1	CYP17A1
GABRG3	GABRA1
RUVBL1	NME1
PPP2CA	PPP3CA
RARG	NR5A1
ADRA2C	HTR1D
ADRA1D	GRM1
HTR1D	OPRK1
GABRA3	GABRG3
GABRA2	GABRG3
NOS2	NOS3
ALAS2	AGXT2
HCAR3	NPY
ADRA1D	HTR2A
ADCY5	GRM7
GRM7	OPRK1
HCK	EGF
ADSS	UBC
ADSSL1	ASPA
RARG	ESR2
ATF2	NR3C1
ADRA1B	GRM1
DRD3	ADRA2A
SHMT2	DAO
ADCY2	NME1
CYP2B6	CYP2A6
OPRM1	ADRA2A
ADCY5	GRM8
HSD3B1	SRD5A3
RARG	PGR
RARB	MED1
CHRNB2	CHRNA3
EDNRA	HTR2B
GRIN2C	GRIN2D
HTR2A	EDNRA
ADRA2B	ADRA2C
GATM	SHMT2
DRD1	ADRB2
NR5A1	PPARG
RARB	PPARD
GRIN2A	GRIN3B
NCOA1	ACOX1
ADRA1A	GRM1
GRM8	ADRA2A
GPR18	ADRA2A
GRM8	ADCY2
GRM4	ADCY2
HTR1B	P2RY12
VDR	UBC
GLDC	ALAS2
DRD5	CRH
ARAF	JUN
THBD	F10
VDR	ESRRG
HTR1D	ADRA2A
RXRG	ESR1
ADRA1D	EDNRA
GCAT	SHMT1
AR	RARB
ALDH18A1	ASNS
LEPREL1	LEPRE1
CHRNA4	CHRNA3
CHRM2	ADRA2A
GLRB	GLRA2
CALM1	CACNA1D

PIPOX	AGXT2
RXRG	ESR2
ADRA1D	ADRA1B
GPR18	P2RY12
ADRA2B	DRD3
DRD5	ADRB2
ESRRG	RXRG
GLRA3	GLRB
PLK1	YWHAE
ADRB2	CRH
PRKAG2	PRKAG3
WARS	FARS2
SHMT2	ALAS2
PTK2B	PRKCB
ADRA2A	OPRD1
F2	FOS
GRIN1	GRIN3B
ADCY5	NME1
ESRRG	RARG
CHRM2	HTR1A
EDNRA	GRM1
DRD3	DRD2
PPP3CA	PPP2CB
TH	IL4I1
ADRA2C	OPRD1
PTGS2	NOS2
ADRA1A	HTR2B
POLA1	NME1
GABRG3	GABRA4
GARS	YARS
PPIA	NFATC1
NOS1	NOS3
ADRA2A	OPRK1
DRD1	DRD5
ADCY5	SUCNR1
FARS2	AARS
SUCNR1	GRM7
PLA2G2A	CYP2B6
GABRG3	GABRA6
GNMT	DAO
CACNA1D	CACNA1C
ADRA1B	PIK3R1
ALAS2	SHMT1
ADRB3	DRD1
SHMT2	ALAS1
ADRA2B	OPRD1
CYBB	RAC1
GAD1	CDO1
SRC	CHRNB1
SCN1B	SCN5A
ADRB1	CRH
CHRNA2	CHRNA4
GNAS	PRKCB
OPRM1	OPRK1
GCAT	AGXT
ADRA2B	OPRK1
IL2	EPOR
GABRA5	GABRA4
ADCY2	EDNRA
ADCY5	HTR1D
ADRA2C	HTR1B
SDHC	OGDH
ADRA2B	DRD4
SUCNR1	GRM8
ASPA	ASNS
GABRA2	GABRA4
PPARG	PPARA
HCK	PIK3CA
ADRB1	PTH
GRM4	SUCNR1
PRKCA	RXRG
MED1	CPT1A
GRM7	HTR1D
ADRA1D	PIK3R1
OPRM1	ADRA2B

DRD1	PTGIR
ESRRG	RARA
ADRA2B	P2RY12
RXRG	PGR
NR1I2	SULT2A1
ESRRG	RARB
PIK3CA	NR3C1
SRC	CHRNA1
CAD	ASPA
MT-CO2	CTNNB1
CHRNA2	CHRNA3
ADRA2C	GRM8
GABRA2	GABRA6
ADRA1A	PIK3CA
CACNB4	CACNB2
DRD5	PTGIR
GABRA4	GABRA1
GRM7	ADRA2A
ADSSL1	GOT2
PRKAG1	PRKAG3
AR	RXRG
ADRA2B	HTR1B
ADRA2B	GRM7
ADRA2B	HTR1A
ADRA1A	PIK3R1
ADRA2C	HTR1A
GOT1	ADSSL1
PLOD3	PLOD1
ADRA1B	PIK3CA
ADCY2	ADRB2
ADSS	GOT2
AOX1	CYP2C8
ADRB3	ADCY2
ADCY2	CRH
CACNB4	CACNB3
GOT1	ADSS
ADRA2B	GRM4
GABRA3	GABRA4
ADCY5	EDNRA
PIK3R1	ANXA1
HCAR3	ANXA1
CACNB2	CACNB3
PPARD	PPARA
UBC	CPT1A
GPR18	HTR1A
GRM4	ADRA2A
HK1	LCT
HTR2A	ADRA1B
ADRA2C	GRM7
SHMT2	PIPOX
ALOX5	PTGS2
SHMT1	DAO
CAD	ACY3
NCOA2	CPT1A
SHMT1	PIPOX
ADRA2B	GRM8
UBC	ITPR1
DRD2	P2RY12
GOT1	ASPA
CACNB4	CACNB1
CACNA1S	CACNA1D
ADRA1D	PIK3CA
ADRA1A	EDNRA
PPARD	PPARG
ADCY2	OPRK1
HTR1B	CXCR1
ABL1	UBC
CACNA1F	CACNA1D
HTR2B	FFAR1
HCAR2	P2RY12
GABRA5	GABRA6
CHRNB2	CHRNB4
NCOA1	HMGCR
CACNA1S	CACNA1C
ADRB1	DRD1

HCK	CXCR1
GATM	GNMT
CXCR1	ADRA2A
PIK3CA	ANXA1
KCNJ11	PRKACA
ANXA1	NPY
GATM	SHMT1
CACNB1	CACNB2
CACNA1F	CACNA1S
ADRB1	ADCY2
GABRA6	GABRA1
GRM1	FFAR1
CYP2B6	PLA2G1B
ADCY2	DRD4
ABCA1	NCOA1
GRM8	ANXA1
GABRG2	GABRG3
CACNA1F	CACNA1C
GRM4	GRM7
GABRB3	GABRB1
GABRA3	GABRA2
OPRM1	P2RY12
GABRA2	GABRA5
ABCA1	SCARB1
RAC1	RAC2
DRD3	ADCY2
PLA2G2A	PISD
UBC	SLC7A2
GLRA1	GLRA2
GABRA3	GABRA6
JUN	CHRNA1
GABRA3	GABRA5
GRM4	GRM8
PROS1	EGF
HNF4G	HNF4A
GATM	PIPOX
IL4I1	TYR
NCOA1	SULT2A1
GABRA3	GABRA1
PTDSS1	PLD2
ADCY2	P2RY12
PRKCA	PRKCB
NR5A1	MED1
CACNB1	CACNB3
DRD2	GPR18
GABRA6	GABRA4
PRKACA	PTGIR
GABRA2	GABRA1
HTR2A	FFAR1
NR0B2	NFKBIA
GPR18	ANXA1
HCAR3	CHRM2
GAD2	CDO1
GABRB1	GABRB2
GRM7	GRM8
GRM4	ANXA1
RARG	RARA
TAT	MIF
AOX1	CYP2B6
GRM4	P2RY12
GATM	GCAT
GNAS	HTR2C
GABRA5	GABRA1
ADCY5	P2RY12
PAH	IL4I1
ADRB1	DRD5
RARG	RARB
HSD3B1	SULT2B1
PLA2G2A	CYP1A2
GRM1	PIK3R1
CTH	SDS
GOT1	DDC
FOS	PPARG
HCAR2	HTR1B
ADRA2B	CXCL10

CHRNG	PTK2B
CXCR1	ANXA1
CHRM2	ANXA1
ADCY2	HTR1D
ABCC8	PRKACA
HCAR3	ADCY2
UBC	EGF
DRD2	ANXA1
GNMT	ALAS1
ADRA1B	HTR2C
GRM8	CXCR1
ADRA2B	ANXA1
SUCNR1	ADCY2
CSAD	BAAT
TARS2	IARS2
ADRA2B	NPY
GPR18	HTR1D
GRM8	GPR18
ADSS	ACY3
SRD5A3	AKR1D1
PLA2G2E	CYP1A2
DRD2	CXCR1
PLA2G2E	PLD2
ADRA2C	OPRK1
PHOSPHO1	ACHE
PYCRL	P4HA2
GRM4	CXCR1
OPRM1	GPR18
PLD1	PTDSS2
ADRA1A	FFAR1
P2RY12	CXCL10
NCOA2	FADS1
HTR1B	ADCY2
ACTA1	NFATC1
PIK3R1	HTR2B
GRM7	CXCL10
ADSS	ASPA
NQO1	GGCX
GCAT	DAO
EGF	PIK3CA
ADCY5	HCAR2
ADRA2C	CXCR1
ADRA2B	ADCY2
GRM8	CXCL10
PIK3CA	FFAR1
HTR1D	P2RY12
PLA2G2E	CYP2C8
P5CR2	P4HA2
ARF6	EGF
ADRA2B	HTR1D
PISD	ESR1
PRKAA1	UBC
ADCY5	DRD3
ADCY2	CXCR1
CXCR1	OPRD1
CHRNG	SRC
NCOA1	CPT1A
PLA2G1B	PTDSS2
GRM7	CXCR1
RAC1	EGF
HCAR2	CXCL10
GRM4	CXCL10
ASPA	GOT2
ADRA2C	GPR18
GOT2	MIF
PYCR1	P4HA1
ADCY5	DRD4
DDC	GOT2
ARG1	ATF2
DDC	IL4I1
ADRA2A	ANXA1
P2RY12	CXCR1
HCAR2	ADCY2
ACY3	ASS1
SUCNR1	NPY

ADCY2	PTGIR
SUCNR1	CHRM2
ACY3	GOT2
GPR18	CXCL10
PHOSPHO1	PCYT1B
MED1	HMGCR
PLA2G1B	PISD
HTR1B	SUCNR1
NCOA1	ALAS1
PRKCB	EGF
GRM8	P2RY12
BAAT	GAD2
OPRM1	CXCL10
CYTH2	EGF
NFATC1	CSNK2A1
ADSS	NFATC1
P4HA1	PYCRL
ALAS2	PIPOX
CHRM2	CXCL10
SUCNR1	OPRD1
F2	ADRA1B
CXCL10	ANXA1
ANXA1	DRD4
DRD3	ANXA1
HCAR3	HTR1D
CTNNB1	EGF
SUCNR1	CXCL10
ADCY2	CXCL10
GATM	ALAS2
SUCNR1	DRD4
FOLH1	ACY3
CXCL10	OPRK1
ADRA2C	HCAR2
ADRA2C	P2RY12
HTR1B	ANXA1
IL4I1	BHMT
HCAR2	ANXA1
DRD3	GPR18
ADCY5	ADRA2C
SUCNR1	HTR1D
IFNB1	ATF2
HCAR3	CXCR1
TYR	GOT2
DRD5	PTH
ADRB1	PTGIR
IL4I1	ASS1
GNMT	ALAS2
GPR18	CXCR1
HCAR3	ADRA2C
SUCNR1	GPR18
HTR1D	CXCR1
HTR1D	ANXA1
HCAR3	ADCY5
CXCR1	DRD4
PLA2G2A	PLD1
GPR18	OPRD1
HCAR3	ADRA2B
PRKCA	EGF
NFATC1	PRKACA
HTR2A	PIK3CA
EDNRA	FFAR1
ADCY5	CXCL10
IL4I1	ASNS
PLD1	DGKD
ADSS	IL4I1
CXCL10	NPY
P2RY12	ANXA1
PIK3R1	EGF
NCOA2	ALAS1
PIPOX	ALAS1
LARS	SARS
PLA2G2A	PTDSS2
PPP2CA	EGF
GRM7	ANXA1
HCAR3	HTR1B

P2RY12	DRD4
HCAR2	HTR1D
CYP1A2	PLA2G1B
HTR1A	CXCR1
HTR2A	PIK3R1
P2RY12	OPRK1
HTR1A	CXCL10
LALBA	LCT
GNAS	DRD5
ADRB2	PTGIR
ADRA2C	NPY
HCAR3	CXCL10
P5CR2	P4HA1
ADCY5	ANXA1
OPRK1	ANXA1
ALDOA	EGF
ADRA2B	SUCNR1
ADCY5	CXCR1
DRD3	CXCR1
ADRA2C	SUCNR1
DDC	HAL
ANXA1	OPRD1
ANXA1	FFAR1
ADRA1D	ANXA1
SUCNR1	CXCR1
PLA2G2A	CYP2C8
PRKACA	RARA
PIK3CA	HTR2B
HCAR2	CXCR1
MT-CO2	NFKBIA
ADRA1B	FFAR1
GRM1	ANXA1
GCAT	AGXT2
CYP2C8	PLA2G1B
PTDSS1	PLD1
SLC25A22	GUCA1A
HTR1D	CXCL10
PLA2G2E	PLD1
DRD3	P2RY12
OPRM1	ADRA2C
GRM4	GPR18
SHMT1	AGXT2
PTPN1	EGF
DRD3	SUCNR1
IL4I1	CAD
ACY3	ASNS
PGM1	NUDT9
ALAS1	DAO
DGKD	PLD2
ADRB2	ARF6
PTDSS1	PLA2G2A
ADRA1B	ANXA1
HCAR2	OPRD1
NCOA2	SULT2A1
CHRM2	CXCR1
MTR	TAT
CYP11B2	SRD5A3
ADRA1D	FFAR1
HCAR2	DRD2
PTK2B	CHRNB1
HCAR2	CHRM2
GPR18	DRD4
OPRM1	SUCNR1
ADCY2	ADRA2A
ADRA2B	CXCR1
OPRM1	HCAR2
ADSSL1	ACY3
CXCL10	ADRA2A
CPT1A	RORA
DRD2	CXCL10
GLRA1	GLRA3
PLA2G1B	FADS2
EDNRA	PIK3R1
PPP2CA	PPP2CB
HCAR3	P2RY12

ALAS1	AGXT2
CXCL10	DRD4
HCAR3	OPRM1
PROS1	ALDOA
HCAR2	GPR18
ADRA1A	ANXA1
EDNRA	PIK3CA
CXCR1	NPY
GPR18	OPRK1
CHRM2	P2RY12
FTCD	MTFMT
TGFBR2	PDPK1
GOT1	MIF
GRM7	GPR18
JUN	CHRNE
ADRA2B	GPR18
PYCR1	P4HA2
HCAR2	HTR1A
PIK3R1	FFAR1
OPRM1	ANXA1
IKBKB	PRKACA
ADRB3	PTGIR
HCAR3	OPRD1
GRM7	P2RY12
MT-CO2	NR0B2
FADS1	MED1
GLRA3	GLRA2
HCAR3	HCAR2
ARG1	SLC25A2
CXCL10	OPRD1
ADCY5	HTR1B
ADCY5	GPR18
GPR18	ADCY2
LARS2	AARS
THBD	JUN
ADRA2B	HCAR2
ADCY5	OPRK1
DRD3	CXCL10
AR	PRKACA
IL4I1	MIF
HCAR2	DRD4
PGR	PRKACA
OPRM1	CXCR1
HTR2A	ANXA1
ADRB3	PTH
ALAS2	DAO
ALAS1	MED1
IL4I1	ADSSL1
HCAR3	HTR1A
ADRA2C	CXCL10
GAD1	BAAT
PHOSPHO1	PCYT1A
ADCY2	POLR2E
IL4I1	ACY3
HTR1B	GPR18
ADCY2	OPRD1
P2RY12	OPRD1
GABRB3	GABRB2
HTR1B	CXCL10
SUCNR1	ANXA1
GPR18	CHRM2
PTDSS1	PLA2G1B
SERPINA1	EGF
SUCNR1	OPRK1
EDNRA	ADRA1B
PLA2G2E	PISD
GPR18	NPY
GOT1	TYR
SUCNR1	DRD2
ACHE	ATF2
HTR2B	ANXA1
SUCNR1	HTR1A
SUCNR1	ADRA2A
PLA2G2E	CYP2B6
CYTH2	ADRB2

GOT1	ACY3
HCAR3	DRD2
ATP6V1C1	INS
VDR	TOP2B
FADS1	NCOA1
ATF2	RORA
ADRB3	DRD5
IL4I1	ASPA
ACTA1	PIK3R1
ADCY5	OPRD1
ADRA2C	ADCY2
NCOA2	HMGCR
GATM	ALAS1
ITGB2	PLAT
PRKACA	PLK1
HCAR3	DRD4
HTR1A	ANXA1
ADRA2C	ANXA1
GCAT	GNMT
ADCY5	POLR2E
CXCR1	OPRK1
ALDH18A1	GOT2
JUN	CDK6
TLR4	PPARA
IKBKB	TP53
ADRB3	PPARG
HTR2A	HTR3A
YARS	SARS
EPRS	AARS2
BCL2	F2
PPARG	SCARB1
CTNNB1	NME1
GSTP1	JUN
PPARA	FAAH
PRKCA	FOS
ALDH18A1	GOT1
TLR4	SFTPD
PGR	PAEP
CACNB1	CACNA1F
XDH	NOS1
UBC	PPAT
HARS	TARS2
XDH	NOS3
ATF6	INS
TNF	NOS3
PRKAG1	HMGCR
PAH	TYR
LARS2	TARS
KARS	AARS
EPRS	IARS2
ATF4	INS
HTR2A	SLC6A4
PGR	PIK3CA
GNAS	IGF2R
PDPK1	PKN3
CYP2B6	HNF4A
TP53	LYZ
TNF	NFKBIA
ADSSL1	PFAS
HMGCR	PRKAG2
TNF	TLR4
TP53	CDK6
TRPV1	FOS
UCKL1	CAD
MT-CO2	COX6A2
SLC13A2	PPIB
UBC	P4HA2
DPYD	TYMS
HTR3A	HTR2C
GARS	TARS2
GRIN1	SRC
PTGS2	SRC
EPRS	FARS2
YARS	KARS
PRODH	PYCR1

IARS2	WARS2
UBC	ASS1
NOS1	GRIN2C
MAF	TP53
PTK2B	TP53
PIM1	UBC
AKR1C3	AR
HCK	RAC2
PTGS2	PPARG
DPP4	IL2
JUN	ESR2
UBC	PIN1
NR1H4	INS
PPARA	PON1
CRH	IL2
RAC1	F2
GLUL	GCLC
ABL1	INS
MGAM	ALAD
PTDSS1	UBC
CTNNB1	CAD
GLUD1	CAD
ABL1	IL2
TARS	AARS
UBC	PRDX5
HDAC2	PPARD
SRC	RAC2
GLUD1	ASNS
GLUD2	CAD
UBC	ACO2
HTR1B	FOS
SCN10A	SCN4B
TP53	PSENEN
HTR1B	HTR3A
ATF4	SLC7A11
PRLR	IL2
YARS2	IARS2
UBC	GLS
GRIA1	GRIA4
GMPS	DPYD
SRC	ITGB2
PPARG	PON1
NFKB2	TP53
TLR4	NOS2
UBC	HIF1AN
PFAS	PRKAG2
UBC	PYGM
TH	SLC6A3
FARS2	AARS2
CHRNA5	CHRNB4
RPS6KA3	ESR1
NOS1	PAPSS1
NOS3	PAPSS1
NOS2	PAPSS1
ACY1	ASPA
PRKAA2	PRKAB2
SHMT2	SHMT1
YARS	WARS
SRC	FURIN
SHMT1	PFAS
FOS	HTR2C
PTGS2	PGR
EPRS	GLUL
FKBP1A	UBC
PRKAA2	UBC
SRC	ITPR1
ABL1	RAC2
ABL1	ESR1
CACNB1	CACNA1B
SYK	ITGB2
HDAC9	CTNNB1
TPI1	GAPDHS
IARS2	AARS
LARS	TARS2
CAD	SARS

HSPA5	TP53
YARS	LARS2
IARS2	FARSB
SRC	GRM1
GMPS	PPAT
LARS2	AARS2
EPRS	DARS2
ATP6V1C1	UBC
TNF	ATF2
VDR	PTH
NOS3	PTH
SCN2A	SCN2B
HTR7	SLC6A4
CHRNA4	FOS
ATF6	UBC
GLS2	TP53
PGR	CA2
YARS	CARS2
CYBB	RAC2
CACNB4	CACNA1B
TLR4	PTGS2
PGR	NME1
FARS2	SARS
OAT	ASS1
EPRS	FARSB
LCN2	INS
UBC	SRR
CAT	GSS
GARS	KARS
SRC	P2RY12
EARS2	AARS2
UBC	CAD
PRLR	UBC
ACTA1	INS
VDR	CYP27B1
SRC	NR3C1
UBC	PPIF
SLC7A8	SLC3A1
KCNJ1	SRC
HARS	AARS
SQLE	PTGIS
LARS	YARS
PPP2CA	PRKCA
NCOA2	NR0B1
PLD1	ARF1
ALDOA	PYGM
TARS2	KARS
HDAC2	NFKBIA
TNF	CYP19A1
GARS	TARS
SDHB	ATP5B
COX5A	PROC
CYP2B6	PPIG
HMGCR	PRKAG3
PAEP	INS
TP53	ANXA1
SHBG	INS
NOS1	NFKB1
DBI	UBC
CYP2C8	PPIG
LARS	AARS2
PRKAA1	HMGCR
TH	NPY
DARS2	YARS2
TRPV1	FAAH
LARS	GARS
HARS	YARS
PRKAA1	NME1
GLUD1	ASS1
PTK2B	RAC2
PTGS2	TLR2
UBC	PTK2B
EPRS	TARS
VKORC1	GGCX
PRKCA	ADCY2

FOS	PRKCB
PIM1	PTGS2
GPI	CSNK2A1
TOP2A	PRKCA
PTGS2	NR3C1
NR0B1	PPARA
UBC	CAT
CA9	SRC
SOAT1	INS
NR0B1	INS
TLR4	TLR2
CACNA1C	INS
NR0B1	NR1I3
CACNA1B	INS
ATF3	INS
GOT1	UBC
TLR4	CFTR
GMPS	UCKL1
TRPV1	INS
CACNA1D	INS
ATF4	PPP1CC
PRKAA1	NOS3
JUN	RAC2
CTNNB1	PLK1
UBC	RAC2
UBC	SLC1A3
SCN8A	SCN2B
PTK2B	NOS3
UBC	GNPDA1
ANXA5	S100B
MAF	IL2
UBC	GBA
ADRB3	PPARA
TOP2A	PRKCB
UBC	ODC1
OPRM1	UBC
LCN2	TLR2
PPP2CA	PRKCB
SRC	SCARB1
CACNA1S	CACNG1
SDHA	ATP5B
NR1I2	TP53
YARS2	AARS
TNF	PPARA
JUN	NOS1
UBC	PGRMC1
SCN8A	SCN4B
PRKAA1	TP53
CACNA1B	SRC
LARS	VARS
SCN3B	SCN2A
CTNNB1	SMARCA5
DPYD	GLUD1
TLR4	SCARB1
CALM1	GRIN2B
KARS	FARS2
PPP3CA	BCL2
ALOX5	SRC
GCLM	GLUL
CA3	INS
TLR4	CRH
WARS	TARS
PRKAG1	CAD
PRKAG2	CAD
HSD3B1	SQLE
FOS	TRPA1
EARS2	GLUL
ABCA1	TLR2
CACNA1B	CACNB2
COX7B	COX5B
PPP3CA	CACNA1C
FECH	UBC
UBC	PLOD3
PRKAA2	PPARA
PPP2CA	INS

CAD	PRKAG3
JUN	NR0B2
ATF5	ATF7
YARS	EARS2
PRKCA	PRKACA
UBC	PAPSS1
HARS	EPRS
ATP2A1	UBC
DPYD	GLUD2
AR	GNMT
PRKAA2	BCL2
NR1I2	SCARB1
GMPS	KARS
PPARG	ATF2
OAT	NAGS
CACNA1A	INS
ADCY2	PRKCB
CSAD	CAD
HDC	CAD
DDC	CAD
PRKACA	ATF2
SRC	S100B
DPP4	HNF1A
PPP2CA	SLC6A4
PRODH	TP53
NR0B1	CTNNB1
PRKCA	PPP2CB
DRD1	GRIN2B
HNF4A	SCARB1
UBC	LCMT2
UBC	NADSYN1
SLC18A2	SLC6A3
FOS	DRD4
TP53	JDP2
ATF4	PTH
HARS	FARS2
F2	P2RY12
WARS	FARSB
AARS	SARS
MAF	PPARG
NOS1	BCL2
GARS	YARS2
PTGS2	PPARD
ITGB2	PIK3CA
IARS2	TARS
MAN1B1	MAN2A1
TOP2A	CTNNB1
ATF5	BCL2
TLR4	UBC
CTH	GCLM
PRKAA2	NOS3
COMT	FOS
AR	ATF3
RAC1	PLD2
OPRK1	SLC6A4
NOS1	CACNA1C
UBC	ACACB
OAT	ASL
TNF	JUN
JUN	GNAS
TNF	DPP4
PRKCA	CTNNB1
AR	TRPM8
PIK3CG	ITGB2
INS	GCLC
ESR2	CRH
TP53	NFKBIA
CTH	PPAT
UBC	SPTLC2
ATF2	CSNK2A1
ALDOA	TPI1
UBC	PPP3R1
NOS3	ATF2
YARS	DARS
SRC	ATP1A1

PRKCA	GRIN2A
PRKAA2	HMGCR
PTGS2	F2
UBC	PPIB
PTGS2	NOS1
HK1	AKR1B1
PDPK1	PPARD
SCN10A	SCN2B
SYK	PLD2
PRKAA2	TP53
UBC	CYBA
SCN8A	SCN3B
FARSB	WARS2
NQO1	TP53
AKR1B1	GCK
GLUD2	ASNS
IGF2R	CTSD
KCNQ3	SRC
ATP5C1	COX5B
SRC	NOS1
COX5A	ATP5B
SYK	CFTR
CTH	UBC
UBC	MGMT
NR1I2	ALAS1
JUN	MT-ND1
RAC1	RHO
ABCA1	PCTP
PTK2B	F2
NOS3	NFKB1
HTR1A	SLC6A4
DARS2	LARS2
TARS2	WARS2
GRM1	GRIN2B
F2	NCF1
PIN1	PPIF
ADCY5	PRKCB
PIN1	NME1
HSPG2	BCL2
ACSL3	PPARA
NR5A1	CTNNB1
JUN	NFKB1
DRD1	GRIA1
TARS2	EPRS
TARS2	LARS2
GARS	IARS2
YARS2	LARS2
CALM1	ADCY2
NOS1	TP53
CYP17A1	SRC
NR1H4	NCOA1
BCL2	S100B
NAGS	ASNS
EPRS	FARSA
COX7B	COX7C
SERPINA1	UBC
CACNB4	CACNA2D2
SRC	PPARD
UBC	PGR
LARS	CARS
UBC	ISYNA1
NR1H4	ESR1
ACACB	OXCT1
NOS1	ITPR1
NR1H4	CYP19A1
OXCT2	ACACB
DPYD	UBC
HNF4A	MTTP
SLC25A12	UBC
PRKCA	ITPR1
SHMT2	GSS
SRC	PPP2CB
SOD2	TP53
ALDOA	PYGL
SHBG	HNF4A

ITPR1	GRM1
FTCD	CAD
TNF	PPARD
CACNA1G	CALM1
SDHC	ATP5C1
SCN1B	SCN1A
PRKCB	ATF2
UBC	PYCR2
SRC	DGKA
RARB	NCOA1
SDHB	COX5B
SHMT1	GSS
CACNA1A	UBC
ACACB	PRKAB1
ABL1	YWHAE
UBC	NQO1
NCOA1	NFKB1
SCN1B	SCN9A
NCOA2	ESRRG
GNAS	SRC
TLR4	CDK6
GATM	ACY3
HSPG2	IL2
GATM	ASS1
MT-ND1	INS
PIK3R1	TLR2
HMGCR	PRKAB1
PTGS1	ESR1
LGALS7	BCL2
ABCA1	CTSD
LARS2	WARS2
BCL2	CRH
PTGS2	PTH
MGMT	MLH1
AR	SARS
F2	RAC2
PRKCA	PPARG
CTH	GCLC
UBC	PYCRL
ALOX5	PTGS1
PLD1	RAC2
EXT1	NOS1
BCL2	METAP2
CACNA1B	UBC
UBC	CTSD
FARSB	LARS2
TP53	PLAT
GPI	SRC
SRD5A1	INS
GATM	ASPA
SLC6A5	GLRA1
CA9	ATF4
TH	SLC18A2
NCF4	PRKCB
SYK	CALM1
DRD1	FOS
NCF2	NOS2
CDA	DCTD
OGDH	ACO2
ARAF	PIK3R1
CHRNA4	ACHE
NOS3	CAT
DARS2	AARS
TLR4	EPOR
SLC6A4	DRD4
TARS2	AARS
RAC1	NFATC1
UBC	GRIN2D
HTR2A	CACNA1S
LSS	PTGIS
SCN1A	SCN2B
FTCD	HAL
GLUD2	ASS1
GRIN1	PRKACA
CARS	TARS

CALM1	GRIN2A
GOT1	GLUD2
ARAF	PRKCB
F2	CYBA
RUVBL1	TP53
NCF2	PRKCB
MT-CO3	BCS1L
YARS2	AARS2
GRIN2A	GRM1
GMPS	IARS
RPS6KA3	PLD1
SCN1B	PRKACA
ANXA1	CFTR
CDO1	GCLC
NCOA2	RXRG
TNF	TRPV1
PRKACA	GRIN2B
HSPA5	CFTR
JUN	ALDH1A1
TNF	TLR2
PPP2CA	NFKB1
PRKCA	ARAF
GMPS	TOP2A
SLC25A10	UBC
NOS2	CYBA
ABL1	ACTA1
AR	CYP19A1
GRIN2A	GRIA1
UBC	RABGGTB
CHRNE	CHRNA1
UBC	RHO
CARS	FARS2
MT-ND1	TP53
NOS1	PTK2B
BGLAP	GGCX
EARS2	YARS2
ITPR1	PRKCB
SERPINC1	UBC
BCL2	NR0B2
PRKACA	ATP1A1
GLUL	ASPA
OAT	CAD
HDAC9	PTGS2
HDAC9	PTH
GOT1	GOT2
SCN10A	SCN3B
HARS	IARS
OAT	PYCR1
MT-CO2	SRC
CACNA1B	CACNA2D1
GCLM	CDO1
PPP2CA	TGFBR2
TP53	DRD4
TARS2	YARS2
PRKAA1	BCL2
AGXT2	DAO
AR	PIM1
PPP3CA	CACNA1D
NR5A1	SCARB1
GRIN2B	GRIA2
CYP2A6	PPIG
SLAMF7	MAF
TNF	ABCA1
PROS1	COX5A
HNF1A	GCK
ESR2	NOS2
IARS	SARS
NR1I3	CYP2B6
GRIA1	GRIN2B
YARS2	FARSB
DBI	CALM1
CTSA	UBC
PTPN1	ATF4
NR5A1	SULT2A1
HAO1	AGXT2

F2	ANXA5
UBC	ESR2
NCF4	NOS2
PTGS2	S100B
ABCC8	PLK1
PGR	CYP19A1
GRIA2	GRIK5
IFNB1	PTGS2
PIK3CG	PGR
PPP1CC	GRIN2B
CYBA	RAC2
GRIN2A	GRIA4
NR0B2	NR3C1
HTR3A	HTR1D
BCL2	PLD2
PTGS2	RAC1
FXN	EPOR
ANXA5	CFTR
HTR1B	SLC6A4
GARS	FARS2
PRKCA	CACNA1A
IL4I1	NAGS
GOT1	CSAD
PTK2B	PPARA
COMT	DRD4
EPRS	NR1I2
GAD1	ASNS
IL4I1	GOT2
GRIN2A	PRKACA
EGF	INS
COX6C	COX6A2
GRIK3	GRIK5
EPRS	YARS2
PRKAG1	CFTR
GRIN1	PPP1CC
PRODH	PPARA
OPRM1	GNAS
SFTPD	LY96
EPRS	GLUD1
EARS2	GLUD2
PRKAG3	CFTR
GLUD2	GOT2
NCOA2	NR5A1
PPP2CA	CACNA1C
UBC	ALAS1
EPRS	GLUD2
UBC	SLC6A3
NR0B1	CYP17A1
TNF	SOD2
CAMP	HDAC9
EARS2	GLUD1
GAD2	ASNS
GLUD1	GCLC
PRKAG2	CFTR
HDAC9	CYP2B6
CAT	GCLC
PRKACA	GRIN2C
GRIN1	GRIA1
PSAP	TP53
PRKACA	GRIN2D
HSD3B1	LSS
DDC	SLC18A2
HARS	SARS
GMPS	VARS
GRIN2A	GRIA2
ATF3	GCK
CACNB4	PPP2CB
DARS	SARS
ADSS	NAGS
HBA1	SCN2A
DPP4	INS
PDPK1	NPRL2
CACNA1G	INS
IKBKB	JUN
CBS	MTRR

GRIK2	GRID2
UBC	NR1I2
GCLM	GLUD1
GRM1	CFTR
HDAC9	SLC1A2
RAB9A	PGRMC1
TNF	CYBA
HTR2A	CACNA1C
CALM1	CACNA1H
CACNA1A	PRKCB
GOT1	IL4I1
SHMT1	SPTLC1
PPP1CC	GRIN2A
OAZ1	UBC
SHBG	CYP19A1
UBC	GRIA1
GAD1	GOT2
GRIA4	GRIN2B
GLUL	SLC1A3
NR5A1	SERPINA1
GOT1	GAD1
GCLM	GAD1
DRD2	COMT
HDAC9	ESR1
DBI	BCL2
GMPS	IARS2
PPP1CC	GRIA1
CACNA2D2	CACNB1
LGSN	GLUL
PPP1CC	CACNB2
ATF5	LCN2
TOP2A	TP53
PPP2CA	CACNB3
EPRS	SARS
GAD2	GOT2
RAC2	NFKBIA
DRD1	GAD2
GOT1	NAGS
GOT1	GAD2
CACNB4	PPP2CA
ALDH18A1	ASPA
SHBG	AR
NOS1	CACNA1D
CACNB3	PPP2CB
PRKCB	TYR
GRIK1	NOS1
HDAC9	PAEP
ACMSD	KYNU
GRID2	GRIK5
PGR	TP53
CACNA1G	CACNA2D1
PTH	INS
PPP1CC	CACNA1C
GRIA1	GRID2
LARS	FARSB
VDR	PPP1CC
CSAD	GOT2
GLS	PCYT1A
PTGS2	ACSL4
GLS	GCLC
HDAC9	ARG2
FARSA	TARS
EPRS	GLS
JUN	MAF
COMT	SLC6A3
PRKACA	CACNA1D
SRC	ACP1
TOP1	ASNS
ADSS	PPAT
GARS	WARS2
PPP1CC	GRIA2
CACNA1S	PRKACA
SCN1B	SCN8A
CA1	ALDH1A2
HTR2A	CACNA1D

EARS2	FARS2
BCL2	FFAR1
NOS1	CFTR
FXN	SDHB
ASPH	UBC
GCLM	GAD2
PPP1CC	GRIN2D
IFNB1	EPOR
IARS	WARS
TRPV1	PTGS2
GCLM	GLS
CACNA1G	NOS1
PSAP	CTSD
PPP1CC	GRIN2C
NAGS	GOT2
PIK3CG	CHRM2
GARS	FARSB
UBC	NR3C2
ATP5C1	UBC
GRIN1	GRIA4
CTNNB1	TP53
SRC	TP53
SDHB	COX4I1
PAH	TH
ADSSL1	NAGS
BCAT1	IL4I1
GOT1	GCLM
GCLM	GOT2
ABCC8	GCK
VARS	YARS
GRID2	GRIA2
BCL2	NOS3
AR	SLC25A4
CTNNB1	ESR1
PLD2	CSNK2A1
SHMT2	SPTLC1
NCOA2	PGR
YARS2	SARS
ATF5	ATF6
GLS	FTCD
PTGS2	EDNRA
CTNNB1	PRKACA
PPP1CC	GRIA4
CBS	SPTLC1
ALDH18A1	ADSS
GLUD1	ASPA
IL4I1	BCAT2
SCN5A	PRKACA
PTGS2	NME1
ALDH18A1	ADSSL1
ADCY5	CALM1
P5CR2	PRODH
PPP1CC	GRIA3
GRIN2A	GRIA3
NOS3	SLC6A7
BCAT1	BCAT2
GRIN2B	GRIA3
HSD17B1	HSD3B1
DRD5	SLC6A3
MT-CO1	BCS1L
GLS	ASPA
GPI	PYGM
TNF	CYBB
UBC	ATF3
MAF	HAS1
PPP2CA	CACNB2
MT-CO1	NOS1
CACNB2	PPP2CB
SLC7A1	GCK
PPP2CA	CACNB1
MUTYH	MLH1
SUCLG1	DPYD
FTCD	ASS1
GARS	VARS
CACNB1	PPP2CB

TNF	NOS1
TNF	MT-ND1
GRIN1	GRIA2
GRIK1	GRID1
PRKACA	GRIA2
ALDH18A1	ACY3
SCN7A	PRKACA
EPRS	GAD1
UBC	SLC1A4
PRKACA	GRIA3
TARS	WARS2
GRIK2	GRIA2
GNAS	GRM1
RXRG	NCOA1
XDH	TLR4
SLC25A13	ASS1
ALAS2	ALAS1
SDHD	IL2
CALM1	GRIN2C
NFKB1	PPP2CB
DPP4	DNPEP
ACACB	ME2
PPT1	ACSL4
PPT1	ACSL3
SHMT1	SPTLC2
CACNA1D	HTR2B
SHMT2	SPTLC2
CALM1	NOS1
UCKL1	UBC
ESR2	PTGIS
CALM1	GRIN2D
CACNA1B	CACNA1G
GATM	ADSSL1
IL4I1	GLUD1
IL4I1	GLUD2
PRKAB1	CFTR
ADSS	GAD2
DPP4	NPY
GRIN3A	PPP1CC
PPP1CC	GRIN3B
PPP2CA	JUN
ESR2	NFKBIA
CALM1	PPP1CC
COX7B	COX6A2
GRIK4	GRIK2
EPRS	GAD2
CTSB	PPARD
SOAT2	SULT2B1
SLC6A9	UBC
LARS2	SARS
PTGS2	RAC2
CALM1	PYGL
UBC	ATF1
GAD1	GCLC
GATM	ASNS
CACNB1	PRKACA
CACNB1	PPP1CC
ADSS	GAD1
GAD2	ASS1
PPT1	UBC
BHMT	NFKB1
ABCA1	S100B
IKBKB	PRKAA1
ASPA	GAD2
NOS1	CACNA1H
PRKAA1	PRKACA
CACNB4	CACNG1
GRIN1	GRIA3
MAOB	GLO1
UBC	NQO2
GRIN1	GRM1
SDHA	ACO2
GATM	IL4I1
UBC	NR0B2
UBC	GGCX

UBC	GLUD2
PPP1CC	ATF2
DARS2	EARS2
PRODH	PYCR2
GLUD2	GLUD1
CARS	UBC
PRKAA2	CFTR
PRKCA	ATF4
ACTA1	PLD2
CACNA1A	CACNB3
AKR1C3	LCN2
ATF4	PRKCB
GAD2	GCLC
DRD2	S100B
PPP2CA	CACNA1D
VDR	FOS
CACNA2D3	PRKACA
CACNA1C	HTR2B
UBC	COX5B
KARS	SARS
SRR	SPTLC2
SRR	SPTLC1
DRD2	PPP2CB
GAD1	ASPA
PRKACA	SCN1A
CACNA2D1	PPP2CB
GATM	CAD
AGXT	SPTLC1
CACNA2D3	PPP2CB
CACNA2D3	PPP1CC
PRKCA	SLC6A3
PPP1CC	CACNG1
CACNA2D2	PPP1CC
PPP2CA	CACNA1S
ALDH18A1	IL4I1
SOD2	RAC2
ADSSL1	GLUD2
SMPD4	TP53
PPP1CC	CACNB3
SDS	SPTLC2
PPP2CA	CACNA2D2
CACNB4	PRKACA
ATF4	PRKACA
GLUD2	FTCD
GAD1	FTCD
GLS	ACY3
SLC8A1	PRKACA
GLUL	FTCD
CACNA1S	PPP2CB
GATM	GOT2
IL4I1	FTCD
SCN4B	PRKACA
PRKACA	GRIN3B
AKR1B1	SDS
EARS2	GLS
GAD1	ACY3
GATM	GOT1
PPP2CA	CACNG1
CACNB4	PPP1CC
FTCD	ASNS
GAD1	EARS2
PPP1CC	TRPM8
CBS	SPTLC2
GAD1	ASS1
ADSS	FTCD
TAT	IL4I1
GOT2	GCLC
GLUD2	ACY3
NAGS	ACY3
FTCD	ACY3
PRKCB	SLC6A3
CACNA2D3	PPP2CA
FTCD	GLUD1
GLUD2	GCLC
PPP1CC	CACNA1D

GAD1	IL4I1
PPP2CA	CACNA2D1
HARS	WARS
CACNA2D2	PRKACA
ADSSL1	GAD2
GLUD1	ACY3
GLUD2	ASPA
GOT1	GCLC
FTCD	ASPA
HDAC9	THBD
GOT1	FTCD
GRIN3A	PRKACA
SOAT1	SULT2B1
ADSSL1	GLUD1
SLC8A1	CKM
AGXT	SPTLC2
KCNJ5	PRKACA
IL4I1	GAD2
CACNG1	PPP2CB
PRLR	IFNB1
FTCD	GOT2
CACNA1C	PPP2CB
ADSS	GLUD1
CACNA1D	PPP2CB
GATM	ADSS
TGFBR2	PPP2CB
UBC	PITPNA
GAD1	ADSSL1
CACNA2D2	PPP2CB
GAD2	ACY3
CACNA2D1	PRKACA
EARS2	GAD2
NAGS	ASPA
GLUL	ACY3
CACNA1S	HTR2B
HDAC2	RXRG
PRKACA	CACNB3
CACNA1S	PPP1CC
CACNA1I	CALM1
GLO1	SDS
CACNA1I	NOS1
SPTLC1	SDS
PRKACA	CACNG1
GCLM	GLUD2
ADSS	GLUD2
MAOB	AKR1B1
FTCD	GAD2
CACNA2D1	PPP1CC
PPP2CA	DRD2
ADSSL1	FTCD
PTPN1	NOS1
MT-CO2	BCS1L
CACNA1B	CACNA1H
GRIN3A	GRIA1
PAICS	ADSS
FABP6	HNF4A
PRKCA	ACTA1
ESR2	CYP19A1
RAB9A	UBC
SOD2	RAC1
HNF4A	FABP2
UBC	RABGGTA
HDAC9	BCL2
TAT	SDS
UBC	CTSB
RPS6KA3	PIK3CA
DBI	CYBB
CARS	TARS2
COX7A1	COX6A2
PPP2CA	CAD
UBC	PCYT1A
SRC	PPARA
CTPS1	PFAS
GRIN3A	GRIA4
AARS	WARS2

ISYNA1	CDIPT
GLO1	UBC
ALDH18A1	PRODH
GMPS	TOP2B
FXN	SDHA
UBC	CCBL2
UBC	ALDH1A2
ABL1	PTPN1
MB	CAT
HTR2A	PTGS2
GPI	PYGL
MT-ND1	NR1I2
COX7A1	COX4I1
WARS	KARS
TOP2A	TYMS
GLUL	SLC1A2
FOS	PIK3CA
LARS	YARS2
YARS	ASL
NOS1	OPRK1
ATF2	ATF1
PRKACA	NCF1
HARS	LARS
WARS	SARS
IARS	WARS2
MT-ND1	CACNA1A
LARS	WARS2
NOS1	GCLC
BCL2	PPP2CB
GRIA1	GRM1
ATF5	ATF3
GMPS	SARS
IKBKB	BCL2
DBI	NOS3
CARS	KARS
KARS	YARS2
GRIA4	GRIN3B
BCAT1	UBC
B2M	UBC
GABRQ	HPGDS
HTR3A	HTR2B
LCMT1	PPP2CB
PRKAG1	PRKACA
PPP2CA	PRKAA1
FECH	ALAS2
EPRS	SQLE
GRIA1	GRIN3B
AGXT	OGDH
HARS	GARS
LARS	WARS
IARS	YARS2
IARS2	CAD
IARS	CAD
COX7B	COX6B1
SDHC	COX5A
ATP5B	COX5B
DDC	HNF1A
VARS	CAD
GRIN3A	GRIA2
TOP2B	CAD
CARS	SARS
ACACB	ACOX1
VDR	JUN
NCF1	NFKB1
ATF6	NR0B2
DBI	PRKCA
TOP2A	CAD
CHRNA2	HSPA5
TRPV1	ABCA1
CACNA2D1	CACNA1H
HDAC9	CSNK2A1
TOP2B	TP53
TARS2	WARS
CACNB2	CACNG1
CTSB	INS

CACNA2D2	CACNA1A
IKBKB	PPP2CB
GRIA3	GRIN3B
GRIN2B	GRIN3B
GRIN3A	GRIA3
UBC	BCAT2
YARS2	TARS
CACNA1B	CACNA1C
GRIA2	GRIN3B
GMPS	FARS2
TOP2B	CSNK2A1
RPS6KA3	PIK3CG
DARS2	AARS2
HMGCR	SQLE
INS	CTSD
SDHC	COX5B
UBC	PYCR1
CACNA1F	CACNA1G
SRR	DAO
ABL1	PIK3CA
GRIN1	DRD1
SHMT1	MTRR
HDAC2	NR3C1
COX6C	ATP5E
SDHB	ATP5A1
KCNJ5	ADRB2
SDHB	UBC
ABCA1	NFATC1
CAT	DAO
ALDH18A1	ACACB
KCNJ5	DRD4
AR	CYP17A1
PFAS	DHODH
HDAC9	TOP1
SCN10A	TRPV1
THNSL1	ODC1
TARS2	FARSA
CYTH2	ITGB2
HTR2C	SLC6A4
SHMT2	CAD
CARS	YARS2
MUTYH	UBC
AR	RUVBL1
HDAC2	ESR1
TARS2	UBC
S100B	GUCA1A
WARS2	SARS
HDAC9	YWHAE
FOS	DRD5
SDHB	COX5A
CARS	WARS2
GMPS	ACACB
CACNA1B	CACNA1D
UBC	CACNA1C
CARS2	AARS2
AARS	CARS2
CARS	AARS2
PRKACA	PRKAG2
WARS	AARS
ATF5	ATF2
TP53	MIF
SERPINC1	COX5A
PRODH	PYCRL
TNF	HSD11B1
ACACB	ATP5A1
UBC	IARS2
TARS	DARS
FARSB	SARS
CARS	IARS2
KCNJ11	INS
SLC6A5	GLRB
BCL2	FOS
ATP2A1	ITPR1
PIK3CG	PRKCB
TOP2A	SARS

DARS2	UBC
GLUL	ASNS
CACNA1B	CACNA1S
PCCB	ACACB
UBC	SH3RF1
LARS2	DARS
CARS	IARS
SLC6A14	SLC7A9
GNMT	BHMT
COX7B	COX5A
GRIK2	KARS
JUN	TOP1
ACHE	CHRNE
ATF3	NFKB1
CHRNA3	CHRNA5
GRIK1	KARS
CAT	NFKB1
CACNA1F	CACNA1H
HDAC2	RARA
SRC	BCL2
CACNB3	CACNG1
SCN1B	SCN3B
UBC	ACP1
CHRNA4	CHRNA5
WARS	YARS2
MT-CO2	NOS3
NR5A1	NCOA1
GMPS	CARS
UBC	COX4I1
GLYAT	BAAT
NR0B1	PGR
HDAC2	AR
ATF7	FOS
ATP5A1	EFTUD1
SCN1B	SCN3A
CHRNE	CHRNB4
CHRNG	CHRNA1
HARS	DARS
OGDH	DHODH
COX8A	PYGM
HARS	GMPS
CACNA1A	CACNB2
WARS	DARS
FARS2	DARS
GMPS	MTAP
ECI2	ACOX1
COX7A1	COX6B1
MB	LYZ
HBA1	INS
BCL2	ESR1
FABP6	ABCB11
F2	INS
GRIA1	GRIA3
CACNA1B	OLFM1
SDHB	ACO2
TNF	INS
KCNJ11	GCK
SLC18A2	HDC
TP53	INS
SLC18A2	SLC6A4
SLC6A5	GARS
THNSL1	CAD
ACY1	UBC
PFAS	CLPP
TOP2B	SARS
VDR	BGLAP
PGRMC1	M6PR
PLD1	ACTG2
ACTG2	PLD2
GABRG2	NUDT9
KCNQ2	SLC25A22
GMPS	CARS2
FURIN	ENPEP
APRT	NQO1
PAPSS1	CAD

NR1I3	MED1
SHMT1	CAD
PITPNA	YWHAE
OAT	AGXT
GMPS	FARSB
CHRNB3	CHRNA6
CTRB1	TAT
UBC	KYNU
COX7C	ATP5E
CHRNA5	CHRNA6
ABL1	PIK3CG
GMPS	JDP2
PTGIR	PTGIS
GABRG2	SCN1B
GABRG2	SCN9A
TH	SLC6A2
DPP4	FOLH1
CHRNA3	CHRNB3
GABRG2	SCN1A
ESRRG	SLC7A1
DPYD	PAPSS1
CHRNA4	CHRNB3
PIM1	CDIPT
MB	LALBA
CHRNA2	CHRNA5
DRD2	SLC6A4
FPGS	SHMT1
SUCLA2	UBC
GMPS	SHMT2
LCT	HNF1A
SUCLG2	UBC
HSD17B1	CYP17A1
HSD17B11	UBC
HNF4G	MTTP
HSPA5	ATP5A1
TNF	NOS2
RNASE1	GLUD1
SDHA	DHODH
UBC	CDIPT
QPRT	ACMSD
NOS3	HMGCR
CARS	WARS
GARS	LARS2
CHRNA4	CHRND
GRIN1	ITPR1
DARS2	PLK1
CACNA1C	CACNG1
EPRS	WARS2
PFAS	ASL
PRLR	SRR
CHRNA2	CHRNB3
HDAC2	HIF1AN
CHRNA7	CHRNA6
APRT	TAT
SLC7A11	SLC3A1
VARS	IARS
CACNA1B	CACNG1
APRT	GOT2
ANXA5	TP53
CHRNA2	CHRNE
GLUL	OGDH
CHRNA7	CHRNA5
GLDC	DPYD
SCN10A	SCN2A
ARAF	FOS
BCS1L	UBC
CHRNB1	CHRNE
GARS	CARS
CHRNA7	CHRNB1
TH	ACHE
CHRNA7	CHRND
PTK2B	GRIN2B
IARS	PFAS
CHRNA2	CHRND
SLC6A4	HTR2B

SCN5A	SCN2A
ACADSB	HIBCH
CHRNG	CHRNA4
HARS	LARS2
SLC25A12	OGDH
GRIK2	GRIN1
FURIN	CTSD
PRKCA	GRIN1
FKBP1A	NME1
CHRNA4	CHRNE
SUCLG1	GNAS
GLUD1	ACO2
YARS	AARS2
UBC	IFT122
SCN8A	SCN2A
SLC26A8	CFTR
SCN3A	SCN2B
GABRG2	SCN2A
VARS	FARS2
DNPEP	ENPEP
TH	HTR7
ARAF	YWHAE
ALDH18A1	ASL
LARS2	CAD
CHRNB1	CHRND
UBC	HSD11B1
CHRNB2	CHRNA5
TAT	GLUL
CHRNA7	CHRNB3
LARS	CAD
FKBP1A	PIN1
PRKCA	TP53
CACNA1H	CACNG1
GMPS	YARS
SLC6A5	SLC32A1
SCN10A	SCN8A
UBC	PLOD1
CACNA1F	CACNA1B
VARS	TARS
ATP5A1	SDHA
PCCB	CAD
AKR1C3	SRD5A1
COX6C	COX7A1
CHRNB1	CHRNA5
WARS	NOS3
CHRNB2	CHRNB3
CHRNB2	CHRNA6
SRC	NR1I2
CHRNE	CHRND
JUN	PPP2CB
TH	GAD1
BCL2	CAT
CARS	LARS2
CACNA1F	CACNG1
CHRNB3	CHRNB4
FARS2	CAD
IARS2	SARS
PGM1	GOT2
SOD2	ACO2
SCN1B	SCN10A
CHRNB1	CHRNA6
CHRNB1	CHRNB3
CHRNA4	CHRNB1
CHRNA3	CHRND
GRIN2C	GRM1
TP53	YWHAE
HTR1D	FOS
MGMT	ESR1
YARS	CAD
CHRNA2	CHRNB1
SLC6A5	GAD1
CHRNA6	CHRNB4
GRIN3A	GRIN3B
APRT	PFAS
RXRA	MED1

CHRNB2	CHRNB1
CAD	AARS2
KARS	LARS2
SLC25A13	UBC
CHRNA5	CHRND
CHRNA6	CHRND
TNF	PTGS1
CAD	AARS
CHRNB3	CHRND
CHRNA4	CHRNA6
CHRNA7	CHRNE
GMPS	NADSYN1
NOS3	NCF1
CHRNB2	CHRNE
XDH	HSD17B6
NAGA	CTSA
ACACB	SHMT2
DRD5	GRIA1
IKBKB	TNF
SOAT1	HMGCR
CHRNA3	CHRNB1
SUCLG2	DPYD
CHRNB1	CHRNB4
NR1I3	NR0B2
UBC	GLTP
SUCLA2	DPYD
PGM1	ACP1
CHRNA2	CHRNA6
SOD2	ATP2A1
ATF6	ATF3
TOP2A	YARS
CTSD	M6PR
CHRNB4	CHRND
INS	SULT2A1
MAOB	UBC
IMMT	SMARCA5
CHRNB2	CHRND
SOD2	ALDH5A1
HDAC2	TOP2A
GPI	GOT1
ATP5B	COX4I1
PTGS1	F2
AKR1C2	SRD5A1
SDHC	UBC
SCN8A	SCN5A
MED1	NR3C1
SCN10A	SCN5A
CHRNE	CHRNB3
CHRNA5	CHRNE
ATF3	ATF1
IARS	GLUL
CYP2B6	HSD17B6
SHBG	ESR1
CHRNA3	CHRNE
GLO1	PGM1
DRD3	COMT
CHRNA3	CHRNA6
CYP17A1	SULT2A1
CRH	INS
INS	CAT
HSD17B6	SULT2A1
CHRNA5	CHRNB3
HBB	SCN2A
CYP2B6	NOS3
WARS	NOS1
NOS3	CYBA
MGAM	PTH
COX5A	F9
AR	HSD17B6
GRIA4	GRIA3
GRIA4	GRIA2
HARS	JUN
CHRNE	CHRNA6
BCL2	INS
PROZ	SERPINC1

ALDH18A1	GCLC
HNF4A	INS
ARAF	CSNK2A1
BHMT	MTRR
SCN9A	SCN2B
NCOA2	NR1I2
TOP2A	EPRS
UBC	ATOX1
XDH	NCF1
GMPS	SHMT1
HIBCH	UBC
FPGS	SHMT2
PCCB	OXCT1
QPRT	UBC
UBC	CHRNA5
VKORC1L1	UBC
MT-CO2	NOS1
IKBKB	PRKAG1
KCNQ2	SCN2A
CACNA1G	CACNG1
BCS1L	HBA1
GSTM1	PPIG
IKBKB	PRKAG2
NCF1	CAT
SERPINA1	LYZ
THNSL1	DPYD
GMPS	WARS
HCK	UBC
HSD17B6	INS
ERVW-1	FURIN
FHIT	TP53
QPRT	SDHA
MAOB	HTR7
F10	COX5A
ATF4	ATF7
WARS	NOS2
CACNA2D2	CACNG1
CACNA2D3	CACNB1
PPP2CA	PIM1
VARS	TARS2
ALDH18A1	OTC
IFNB1	PTGS1
SERPINA1	HNF4A
ECI2	ACADSB
AMY1A	LCT
GRIK1	GRIN1
GLDC	OGDH
TYMS	MTRR
DPYD	PFAS
CACNA1A	CACNA2D1
SUCLA2	ACO2
TLR4	JUN
GRM1	GRIN2D
NR1I2	PPIG
SCN4B	SCN1A
NCOA2	HNF4A
FKBP1A	PPIB
ERVW-1	SLC1A4
MT-CO2	SLC25A4
IARS	FARSA
PCCB	OXCT2
PRKACA	FOS
PPP3CA	JUN
YARS	SHMT2
TOP2B	EPRS
KYNU	ASNS
HDAC2	TOP2B
GRM4	GRIN2B
GMPS	AARS
DRD5	CALY
DRD1	SLC6A3
F2	GRM1
DRD5	SLC6A4
HDAC2	MAOB
THNSL1	ALDH18A1

HARS	DARS2
COMT	PRODH
GRIN2A	DRD1
SLC18A2	SLC6A2
GMPS	EARS2
HNF4G	FABP2
PTPN1	NOS3
PRKAA2	PPARG
SRC	CSNK2A1
SCN3B	SCN3A
GRM4	GRIN2A
VARS	AARS
TOP2B	YARS
YARS	DARS2
COX7A1	COX5B
GMPS	GARS
UBC	PISD
OAT	GLUL
YARS	SHMT1
DARS2	KARS
CYP1A2	NR1I2
UBC	NPRL2
CDA	UNG
VARS	ATP5A1
PTGS1	TLR2
EARS2	LARS2
QPRT	NADSYN1
TLR4	PTGS1
SHBG	CYP17A1
ABL1	TOP1
TH	CRH
MAOB	SLC6A3
ISYNA1	SMARCA5
FABP6	FABP2
HTR2A	COMT
NOS2	NFKB1
SIGMAR1	PTGIS
INS	NFKB1
EARS2	ACACB
BCL2	RAC2
ECI2	ACACB
PRSS1	CFTR
DDC	ABAT
SRC	EDNRA
GARS	CHRNA4
DDC	SLC6A3
YARS	OTC
PPP2CA	TP53
KARS	FARSB
UBC	ALDH1A1
CSNK2A1	NME1
GOT1	CKM
PROS1	F10
YARS	PARS2
LGSN	CAD
NR1I3	ABCB11
CTPS1	EPRS
TOP2B	FARS2
TOP2A	FARS2
CACNA1S	ATP2A1
HSD17B6	ESR1
ACSL3	ACACB
TOP2A	DPYD
MGAM	LYZ
GRIN1	PTK2B
SUCLG1	ATP5C1
JUN	SDHC
FKBP1A	RXRB
HTR7	NPY
SUCLG2	CAD
RXRA	FKBP1A
CYBA	CAT
FKBP1A	RXRG
CYP19A1	INS
KCNJ11	SCN11A

SUCLA2	CAD
GRIN2C	GRIA1
UBC	DNPEP
PRKAA2	NME1
GRIA1	GRIN2D
PRKAA1	PPARG
TOP2A	JUN
GLO1	ACP1
GRIA4	GRIN2D
SRC	PRKACA
ATP5A1	SLC1A4
PGR	CTSD
HTR3A	SLC6A4
CYP2A6	CHRNA6
AR	BCL2
MGAM	INS
ABCA1	HMGCR
TOP2B	PFAS
CYP19A1	SULT2A1
LARS	DARS2
DRD3	SLC6A3
AR	INS
NOS1	CYP2B6
ACACB	ACSL4
ACSL3	ACOX1
SDHC	TP53
CYP17A1	COMT
LYZ	CAT
TH	MAOB
HSD17B6	CAT
JUN	SARS
TH	COMT
GLYAT	GLS
HNF4G	PMP2
HNF4G	FABP6
GRIK1	GRIN2A
GRIK1	GRM1
TOP2A	PFAS
HNF4A	PMP2
PAICS	ADSSL1
SCN9A	SCN4B
CTNNB1	ATP5A1
DPYD	OTC
ABAT	CSAD
COX5A	COX7A1
ACACB	PFAS
CTPS1	DHODH
GRIK1	UBC
GRM7	GRIN2A
NOS3	CFTR
ECI2	ACOT13
PISD	CDIPT
S100B	DRD4
PTGS2	CSNK2A1
PRKCA	GRM7
FECH	ALAS1
UBC	CA2
TOP2B	DPYD
GMPS	CTNNB1
PTPN1	ESR1
UBC	CKM
CACNA1B	MT-ND1
ACADSB	ACACB
ABL1	CAT
NR1I3	NCOA1
CACNA2D2	CACNB2
GMPS	GAMT
BCL2	HSPA5
TARS	CAD
DRD5	GAD2
TARS2	CAD
HTR1A	FOS
ACMSD	AADAT
CHRNA4	HSPA5
GPI	GOT2

BCL2	ANXA5
TPI1	PYGM
XDH	CYBA
GRIK2	GRIN2A
GRIN2C	GRIA4
SHMT2	GLUD2
MT-ND1	CSNK2A1
TOP2B	ESR1
RAC1	PKN3
TNF	NFATC1
PDPK1	PRKACA
JUN	CAT
ACOX1	COX6B1
KARS	WARS2
ATF4	MAF
GRIK2	GRIN2D
GRM4	GRIN2D
HARS	YARS2
MGAM	CAT
GRIK2	GRIN2C
GRIA2	GRIN2D
SCN1A	SCN3B
SHMT1	BHMT
GRIK1	GRIN2D
GRM7	GRIN2B
GRIN2C	GRIA2
SDHC	MT-CO2
CYBB	OTC
DARS2	IARS2
RNASE1	GLUD2
LARS	CARS2
ATF7	ATF1
PARS2	LARS2
GRIK1	GRIN2B
JUN	AARS
PTK2B	ACP1
SCN4B	SCN3A
GRIN2A	DRD5
WARS	LARS2
GCLM	CAT
SDHD	COX5B
UBC	GABRB3
GRIK2	GRIN2B
GRIK2	GRM1
HARS	AARS2
TOP2A	MLH1
SUCLG1	ACO2
THNSL1	NAGS
ABAT	HDC
SLC18A2	SLC32A1
MT-CO1	COX7A1
ACSL4	ACOX1
GRM1	GRIA4
RPS6KA3	JUN
GMPS	AARS2
DPYD	GLUL
NUDT9	GABRA1
MB	CKM
CARS2	SARS
GPT2	GLUD1
DDC	HTR7
UBC	PKN3
KARS	GRIK5
ALDH18A1	EPRS
ATF3	FOS
TNF	SLC1A2
RPS6KA3	NFKBIA
MT-CO1	NOS3
PARS2	FARS2
PFAS	PAPSS1
MT-CO3	COX7A1
GRIK1	GRIN2C
ATF5	FOS
HSD3B1	SIGMAR1
JUN	PIN1

GRIA3	GRIN2D
TOP1	PPP1CC
NCOA1	HNF4A
MT-CO2	SDHA
SCN4A	SCN4B
DRD5	GRIN2B
CAD	ODC1
SEC14L2	OTC
PRDX5	CAT
HIBCH	ACACB
OPRM1	GSTM1
TRPM8	FOLH1
GRIN2C	GRIA3
NUDT9	GABRG1
HSPA5	CAD
THBD	PROCR
HTR7	SLC6A3
CARS	FARSB
HPGDS	GSS
HTR1A	SLC6A3
BCL2	CDK6
CACNA2D2	CACNA1D
TOP1	CSNK2A1
CBS	PRKAG3
QPRT	KYNU
HTR2A	SLC6A3
SLC25A15	ASL
UBC	GCLC
ACTA1	RUVBL1
TOP2B	MLH1
LARS	ARG1
HDC	HNF1A
NR1I2	ALAS2
UBC	PRKAG2
SHMT2	GLUL
GRIN2A	NOS3
PRKCA	NFATC1
OPRM1	CALM1
AKR1C1	SRD5A1
MT-CO2	COX7A1
RUVBL1	ACTG2
ALDH5A1	GABRB2
CARS	ACACB
TNF	F2
DRD2	GRIA2
CACNA2D2	CACNB3
GLUL	SLC1A1
SERPINA1	PRSS1
GRIK1	GRIN3A
HARS	VARS
EARS2	CARS2
NCOA1	TP53
SLC6A5	GAD2
CARS	EARS2
RORA	NME1
NOS3	GRIN2B
NOS3	CYP19A1
PTGS1	EDNRA
ACACB	SHMT1
SUCLG2	KCNQ2
TH	MB
GRM4	GRIN2C
LARS	PARS2
ATF7	JDP2
RXRA	NCOA1
PFAS	IARS2
PFAS	TARS
NCOA2	PPARD
NOS3	GCLC
SDHD	ATP5C1
SCN4A	SCN2B
CYTH2	UBC
ATF4	JDP2
RXRA	NCOA2
CACNA2D2	CACNA1C

PPIA	FKBP1A
MGAM	NADSYN1
NAGS	FARS2
ADRB1	ADRA2A
JUN	AARS2
TARS2	CARS2
CARS	VARS
PGR	FOS
DPYD	ACACB
TARS	CARS2
NFS1	ACO2
GRIK3	GRIN2D
HARS	SHMT1
NQO1	GOT2
PSAT1	SHMT1
ACHE	CAT
ACACB	HMGCR
YARS	UCKL1
ACTA1	TP53
YARS	ALDH18A1
ABL1	BCL2
PRKCA	GRIA2
HSPA5	LCT
CACNA1A	CACNG1
EARS2	CAD
CACNA2D2	CACNA1G
WARS	FARSA
HIBCH	OXCT1
SHMT2	KARS
NOS2	OPRK1
GRIK3	GRIN3B
ALDH18A1	DPYD
IARS2	LARS2
SLC6A14	SLC7A8
RXRA	RARG
YARS	DPYD
TNF	RAC1
UBC	FURIN
LARS	ARG2
TOP2B	TYMS
SRC	ANXA1
ESRRG	NCOA1
DRD4	GABRA1
UBC	LALBA
F7	TP53
SRC	NR3C2
PGM1	ADH1B
RXRA	RARB
GLDC	GLUL
PFAS	GLUL
GMPS	EFTUD1
IARS	EARS2
RAC1	PRKACA
PRKCA	RHO
CBS	PAPSS1
GSTP1	MLH1
PHGDH	SMARCA5
ABCB11	NR0B2
GRIK3	GRIN2B
LARS	TH
EPRS	WARS
CSAD	AGXT2
GRIK3	GRIN2A
UBC	EARS2
GRIK1	GRIN3B
PGM1	GOT1
GRIK2	GRIN3A
VARS	UBC
ACACB	ACCS
SDHC	COX6C
CALM1	YWHAE
NR0B1	NR3C1
ABCA1	SOAT1
GRIK3	GRIN3A
EFTUD1	CAD

HARS	TOP2A
SRD5A1	HSD11B1
SYK	EPOR
NOS1	CAT
GOT2	CKM
SDHD	COX5A
MGAM	MT-CO3
NOS2	GCLC
TOP2A	FARSA
COX6C	ALDH2
GRIK3	GRIN2C
SDHC	FOS
PAH	HAL
JUN	PTGS1
LGSN	DPYD
TP53	IL2
GMPS	GLUL
GRIN3A	GRIN2D
CHRNA4	SRC
TOP2B	FARSA
PHGDH	SLC1A4
TOP2A	UNG
RHO	GUCA1A
HDAC2	BCL2
HTR7	SLC6A2
GRIN3A	GRIN2C
ERVW-1	IL2
HTR1A	CRH
NOS3	PPARG
GAD1	GABRB2
FABP6	PMP2
GLYAT	PCYT1A
F7	COX5A
NAGA	M6PR

Table S4 Detailed information of 66 QXHC candidate targets in the treatment of menstrual disorders

Herbs	Chemical_components	Candidate targets
CH	caprylic_acid	ABL1
CH	caprylic_acid	CALM1
CH	caprylic_acid	INS
CH	caprylic_acid	PRKACA
CH	caprylic_acid	TLR4
CH	cresol	INS
CH	heptanoic_acid	ABL1
CH	heptanoic_acid	CALM1
CH	heptanoic_acid	INS
CH	heptanoic_acid	PRKACA
CH	heptanoic_acid	TLR4
CH	hexadecanoic_acid	ABL1
CH	hexadecanoic_acid	CALM1
CH	hexadecanoic_acid	INS
CH	hexadecanoic_acid	PRKACA
CH	hexadecanoic_acid	TLR4
CH	pelargonic_acid	ABL1
CH	pelargonic_acid	CALM1
CH	pelargonic_acid	INS
CH	pelargonic_acid	PRKACA
CH	pelargonic_acid	TLR4
CS	salicylic_acid	PTGS2
CX	Linoleic_acid	PTGS2
CX	arachic_acid	ABL1
CX	arachic_acid	CALM1
CX	arachic_acid	INS
CX	arachic_acid	PRKACA
CX	arachic_acid	TLR4
DG	arginine	ASS1
DG	arginine	NOS2
DG	arginine	NOS3
DG	glutamic_acid	ALDH18A1
DG	glutamic_acid	ASNS
DG	glutamic_acid	EARS2
DG	glutamic_acid	EPRS
DG	glutamic_acid	GLUD1
DG	glutamic_acid	GLUD2
DG	glutamic_acid	GLUL
DG	glutamic_acid	GOT1
DG	glutamic_acid	GOT2
DG	glutamic_acid	GRIA1
DG	glutamic_acid	GRIN2A
DG	glutamic_acid	GRIN2B
DG	glutamic_acid	GRIN2D
DG	glycine	GARS
DG	glycine	GRIN2A
DG	isoleucine	IARS
DG	leucine	LARS
DG	lysine	KARS
DG	molalanine	AARS
DG	p-cresol	INS
DG	phenylalanine	CRH
DG	phosphatidylserine	PRKCA
DG	proline	EPRS
DG	serine	SARS
DG	tryptophane	WARS
DG	tyrosine	YARS
GC	Urea	CTNNB1
HH	arachic_acid	ABL1
HH	arachic_acid	CALM1
HH	arachic_acid	INS
HH	arachic_acid	PRKACA
HH	arachic_acid	TLR4
HH	linoleic_acid	PTGS2
HH	lysine	KARS
NX	arginine	ASS1
NX	arginine	NOS2
NX	arginine	NOS3
NX	glutamic_acid	ALDH18A1
NX	glutamic_acid	ASNS
NX	glutamic_acid	EARS2
NX	glutamic_acid	EPRS
NX	glutamic_acid	GLUD1
NX	glutamic_acid	GLUD2

NX	glutamic_acid	GLUL
NX	glutamic_acid	GOT1
NX	glutamic_acid	GOT2
NX	glutamic_acid	GRIA1
NX	glutamic_acid	GRIN2A
NX	glutamic_acid	GRIN2B
NX	glutamic_acid	GRIN2D
NX	glycine	GARS
NX	glycine	GRIN2A
NX	leucine	LARS
NX	phenylalanine	CRH
NX	proline	EPRS
NX	serine	SARS
NX	tryptophane	WARS
NX	tyrosine	YARS
TR	linoleic_acid	PTGS2
TR	tryptophane	WARS
ZQ	ephedrine	ATF2
ZQ	ephedrine	ATF4
ZQ	ephedrine	FOS
ZQ	ephedrine	JUN
ZQ	ephedrine	NFATC1
ZQ	ephedrine	TNF
ZQ	pseudoephedrine	ATF2
ZQ	pseudoephedrine	ATF4
ZQ	pseudoephedrine	FOS
ZQ	pseudoephedrine	JUN
ZQ	pseudoephedrine	NFATC1
ZQ	pseudoephedrine	TNF
CH	pregnenolone	GRIN2A
CH	pregnenolone	GRIN2B
CH	pregnenolone	GRIN2D
CH	pregnenolone	PPARA
CH	kaempferol	PTK2B
GC	2-Methyl-1,3,6-trity......	CSNK2A1
CX	chryaophanol	CSNK2A1
CX	chrysophanic_acid	CSNK2A1
DS	1,8-Dihydroxy-3-methyl-anthraquinone	CSNK2A1
CH	caprylic_acid	PTGS2
CH	heptanoic_acid	PTGS2
CH	hexadecanoic_acid	PTGS2
CH	pelargonic_acid	PTGS2
CX	Linoleic_acid	ABL1
CX	Linoleic_acid	CALM1
CX	Linoleic_acid	INS
CX	Linoleic_acid	PRKACA
CX	Linoleic_acid	TLR4
CX	arachic_acid	PTGS2
HH	arachic_acid	PTGS2
HH	linoleic_acid	ABL1
HH	linoleic_acid	CALM1
HH	linoleic_acid	INS
HH	linoleic_acid	PRKACA
HH	linoleic_acid	TLR4
TR	linoleic_acid	ABL1
TR	linoleic_acid	CALM1
TR	linoleic_acid	INS
TR	linoleic_acid	PRKACA
TR	linoleic_acid	TLR4
WY	linderaic_acid	PTGS2
CH	caproic_acid	ABL1
CH	caproic_acid	CALM1
CH	caproic_acid	INS
CH	caproic_acid	PRKACA
CH	caproic_acid	TLR4
DG	arginine	NOS3
NX	arginine	NOS3
DG	histidine	HARS
CH	guercetol_	PTK2B
CH	quercetol_	PTK2B
CX	Quercetin	PTK2B
ZQ	quercetin	PTK2B
DG	adenine	ACACB
CH	phenol	INS
DG	phenol	INS
DS	baicalin	CSNK2A1
DS	baicalin	PRKACA

DS	baicalin	PRKCA
DS	baicalin	PRKCB
DS	baicalin	SYK
CH	caproic_acid	PTGS2
CX	Linoleic_acid	PTGS2
HH	linoleic_acid	PTGS2
TR	linoleic_acid	PTGS2
WY	linderaic_acid	ABL1
WY	linderaic_acid	CALM1
WY	linderaic_acid	INS
WY	linderaic_acid	PRKACA
WY	linderaic_acid	TLR4
DG	2,4-dihydroxyacetophenone	PTGS2
DG	valine	CRH
NX	valine	CRH
ZQ	naringenin	PTK2B
DS	przewalskin	PPP2CA
DS	przewalskin	PPP2CB
DS	przewalskin	PRKCA
DS	przewalskin	PRKCB
DG	anisic_acid	PTGS2
DG	valine	AARS
NX	valine	AARS
DG	phenylalanine	YARS
JG	platycodin_A	PPP1CC
JG	platycodin_B	PPP1CC
JG	platycodin_C	PPP1CC
JG	platycodin_D	PPP1CC
JG	platycodin_D-2	PPP1CC
JG	platycodin_D-3	PPP1CC
JG	polygalacin_D-2	PPP1CC
NX	phenylalanine	YARS
ZQ	(-)-synephrine	TNF
CX	isorhamnetin	PTK2B
GC	Hispidulin	PTK2B
CS	gallic_acid	PTGS2
DG	arginine	NOS1
HH	cholesterol	GRIN2A
HH	cholesterol	GRIN2B
HH	cholesterol	GRIN2D
HH	cholesterol	PPARA
NX	arginine	NOS1
TR	7-dehydroavenasterol	GRIN2A
TR	7-dehydroavenasterol	GRIN2B
TR	7-dehydroavenasterol	GRIN2D
TR	7-dehydroavenasterol	PPARA
TR	campesterol	GRIN2A
TR	campesterol	GRIN2B
TR	campesterol	GRIN2D
TR	campesterol	PPARA
CH	2-nonenoic_acid	PTGS2
CH	caprylic_acid	PTGS2
CH	heptanoic_acid	PTGS2
CH	hexadecanoic_acid	PTGS2
CH	pelargonic_acid	PTGS2
CX	2-tetradecenoic_acid	PTGS2
CX	arachic_acid	PTGS2
CX	hexadecenoic_acid	PTGS2
GC	Ruvoside	PPP1CC
HH	arachic_acid	PTGS2
WY	linderaic_acid	PTGS2
CX	kaikasaponin_III	PPP1CC
DG	arginine	NOS2
NX	arginine	NOS2
GC	Licoricesaponine_D3	GNAS
CH	valeric_acid	ABL1
CH	valeric_acid	CALM1
CH	valeric_acid	INS
CH	valeric_acid	PRKACA
CH	valeric_acid	TLR4
JG	platycogenic_acid_A	NOS2
DG	o-cresol	INS
DG	tyrosine	YARS
GC	2-Methyl-1,3,6-trity......	CSNK2A1
NX	tyrosine	YARS
JG	methyl_platyconate-_A	PPP1CC
NX	inokosterone	NOS2

CX	chryaophanol	CSNK2A1
CX	chrysophanic_acid	CSNK2A1
DG	phenylalanine	YARS
DG	serine	GOT1
DS	1,8-Dihydroxy-3-methyl-anthraquinone	CSNK2A1
GC	Glyyunnanprosapogenin_D	GNAS
NX	phenylalanine	YARS
NX	serine	GOT1
CX	soyasaponin_I	PPP1CC
JG	deapioplatycodin_D	PPP1CC
CH	guercetol_	CSNK2A1
CH	guercetol_	PRKACA
CH	guercetol_	PRKCA
CH	guercetol_	PRKCB
CH	guercetol_	SYK
CH	quercetol_	CSNK2A1
CH	quercetol_	PRKACA
CH	quercetol_	PRKCA
CH	quercetol_	PRKCB
CH	quercetol_	SYK
CX	Quercetin	CSNK2A1
CX	Quercetin	PRKACA
CX	Quercetin	PRKCA
CX	Quercetin	PRKCB
CX	Quercetin	SYK
HH	dopa	YARS
ZQ	quercetin	CSNK2A1
ZQ	quercetin	PRKACA
ZQ	quercetin	PRKCA
ZQ	quercetin	PRKCB
ZQ	quercetin	SYK
CS	β-sitosterol	GRIN2A
CS	β-sitosterol	GRIN2B
CS	β-sitosterol	GRIN2D
CS	β-sitosterol	PPARA
CX	sophoradiol	GRIN2A
CX	sophoradiol	GRIN2B
CX	sophoradiol	GRIN2D
CX	sophoradiol	PPARA
CX	β-Sitosterol	GRIN2A
CX	β-Sitosterol	GRIN2B
CX	β-Sitosterol	GRIN2D
CX	β-Sitosterol	PPARA
DG	arginine	NOS1
DG	arginine	NOS2
DG	β-sitosterol	GRIN2A
DG	β-sitosterol	GRIN2B
DG	β-sitosterol	GRIN2D
DG	β-sitosterol	PPARA
DS	stigmasterol	GRIN2A
DS	stigmasterol	GRIN2B
DS	stigmasterol	GRIN2D
DS	stigmasterol	PPARA
GC	Gamma-sitosterol	GRIN2A
GC	Gamma-sitosterol	GRIN2B
GC	Gamma-sitosterol	GRIN2D
GC	Gamma-sitosterol	PPARA
HH	stigmasterol	GRIN2A
HH	stigmasterol	GRIN2B
HH	stigmasterol	GRIN2D
HH	stigmasterol	PPARA
HH	β-sitosterol	GRIN2A
HH	β-sitosterol	GRIN2B
HH	β-sitosterol	GRIN2D
HH	β-sitosterol	PPARA
NX	arginine	NOS1
NX	arginine	NOS2
SM	β-sitosterol	GRIN2A
SM	β-sitosterol	GRIN2B
SM	β-sitosterol	GRIN2D
SM	β-sitosterol	PPARA
TR	citrostadienol	GRIN2A
TR	citrostadienol	GRIN2B
TR	citrostadienol	GRIN2D
TR	citrostadienol	PPARA
TR	β-sitosterol	GRIN2A
TR	β-sitosterol	GRIN2B

TR	β-sitosterol	GRIN2D
TR	β-sitosterol	PPARA
XF	cyperolone	GRIN2A
XF	cyperolone	GRIN2B
XF	cyperolone	GRIN2D
XF	cyperolone	PPARA
YHS	β-sitosterol	GRIN2A
YHS	β-sitosterol	GRIN2B
YHS	β-sitosterol	GRIN2D
YHS	β-sitosterol	PPARA
GC	Licoricesaponine_G2	PPP1CC
GC	Licoricesaponine_J2	PPP1CC
GC	Licoricesaponine_K2	PPP1CC
CS	β-amyrin	GRIN2A
CS	β-amyrin	GRIN2B
CS	β-amyrin	GRIN2D
CS	β-amyrin	PPARA
DS	sugiol	PPP2CA
DS	sugiol	PPP2CB
DS	sugiol	PRKCA
DS	sugiol	PRKCB
XF	cyperol	GRIN2A
XF	cyperol	GRIN2B
XF	cyperol	GRIN2D
XF	cyperol	PPARA
GC	Methyl-24-hydro....	NOS2
DG	arginine	NOS3
NX	arginine	NOS3
XF	isocyperol	GRIN2A
XF	isocyperol	GRIN2B
XF	isocyperol	GRIN2D
XF	isocyperol	PPARA
DG	glutamic_acid	GLUL
NX	glutamic_acid	GLUL
SM	methylcimicifugoside	PPP1CC
DG	leucine	EGF
NX	leucine	EGF
GC	Isoramanine	GRIN2A
GC	Isoramanine	GRIN2B
GC	Isoramanine	GRIN2D
GC	Isoramanine	PPARA
SM	cimicifugoside	PPP1CC
CH	kaempferitrin	CSNK2A1
CH	kaempferitrin	PRKACA
CH	kaempferitrin	PRKCA
CH	kaempferitrin	PRKCB
CH	kaempferitrin	SYK
CH	3-O-acetylsaikosaponin_d	PPP1CC
CH	saikosaponin_A	PPP1CC
CH	saikosaponin_b1	PPP1CC
CH	saikosaponin_b2	PPP1CC
CH	saikosaponin_c	PPP1CC
CH	saikosaponin_d	PPP1CC
CH	saikosaponin_e	PPP1CC
DG	adenine	PRKACA
DG	tyrosine	YARS
GC	Lincoricesaponine_A3	PPP1CC
NX	tyrosine	YARS
DS	oleanolic_acid	GRIN2A
DS	oleanolic_acid	GRIN2B
DS	oleanolic_acid	GRIN2D
DS	oleanolic_acid	PPARA
DS	ursolic_acid	GRIN2A
DS	ursolic_acid	GRIN2B
DS	ursolic_acid	GRIN2D
DS	ursolic_acid	PPARA
GC	Glycyrrhizic_acid	GNAS
GC	Licoricesaponine_C2	GNAS
GC	Licoricesaponine_H2	GNAS
DG	phosphatidylglycerol	PRKCA
JG	chondrillasterol	GRIN2A
JG	chondrillasterol	GRIN2B
JG	chondrillasterol	GRIN2D
JG	chondrillasterol	PPARA
DG	glutamic_acid	ADSS
DG	glutamic_acid	ASNS
DG	glutamic_acid	ASS1

DG	glutamic_acid	CAD
DG	glutamic_acid	GOT1
DG	glutamic_acid	GOT2
GC	Neohancoside_A	PPP1CC
JG	platycodigenin	NOS2
NX	glutamic_acid	ADSS
NX	glutamic_acid	ASNS
NX	glutamic_acid	ASS1
NX	glutamic_acid	CAD
NX	glutamic_acid	GOT1
NX	glutamic_acid	GOT2
ZQ	epoxybergamottin	PIK3CA
ZQ	epoxybergamottin	PIK3R1
CX	senkyunolide_P	CACNA1B
CX	senkyunolide_P	CACNA1C
DG	tyrosine	PRKACA
DS	baicalin	PTK2B
NX	tyrosine	PRKACA
ZQ	hesperetinv	PTK2B
DS	paramiltioic_acid	PPP1CC
DS	salviol	PPP2CA
DS	salviol	PPP2CB
DS	salviol	PRKCA
DS	salviol	PRKCB
CH	2-nonenoic_acid	ABL1
CH	2-nonenoic_acid	CALM1
CH	2-nonenoic_acid	INS
CH	2-nonenoic_acid	PRKACA
CH	2-nonenoic_acid	TLR4
CH	2-octenoic_acid	PTGS2
CH	caproic_acid	PTGS2
CH	valeric_acid	PTGS2
CS	salicylic_acid	PTGS2
CX	2-tetradecenoic_acid	ABL1
CX	2-tetradecenoic_acid	CALM1
CX	2-tetradecenoic_acid	INS
CX	2-tetradecenoic_acid	PRKACA
CX	2-tetradecenoic_acid	TLR4
CX	hexadecenoic_acid	ABL1
CX	hexadecenoic_acid	CALM1
CX	hexadecenoic_acid	INS
CX	hexadecenoic_acid	PRKACA
CX	hexadecenoic_acid	TLR4
CX	octadecatrienoic_acid	PTGS2
CX	tetradecadienoic_acid	PTGS2
DG	anisic_acid	PTGS2
DG	phenylalanine	YARS
DG	tyrosine	CRH
NX	inokosterone	PPP1CC
NX	phenylalanine	YARS
NX	tyrosine	CRH
CH	3,6-O,O-diacetylsaikosaponin_b2	PPP1CC
CH	saikosaponin_b3	PPP1CC
CH	saikosaponin_b4	PPP1CC
GC	Methyl3-O-beta-D-.....	PPP1CC
GC	Uralsaponin_A	PPP1CC
GC	Uralsaponin_B	PPP1CC
CH	kaempferol	CSNK2A1
CH	kaempferol	PRKACA
CH	kaempferol	PRKCA
CH	kaempferol	PRKCB
CH	kaempferol	SYK
DS	miltipolone	PPP2CA
DS	miltipolone	PPP2CB
DS	miltipolone	PRKCA
DS	miltipolone	PRKCB
NX	rubrosterone	GRIN2A
NX	rubrosterone	GRIN2B
NX	rubrosterone	GRIN2D
NX	rubrosterone	PPARA
YHS	glaucine	RAC1
YHS	glaucine	RAC2
GC	Glyeurysaponin	GNAS
DG	alexandrin	PPP1CC
DG	eleutheroside_A	PPP1CC
DS	alexandrin	PPP1CC
DS	daucosterol	PPP1CC

DS	eleutheroside_A	PPP1CC
HH	alexandrin	PPP1CC
HH	eleutheroside_A	PPP1CC
TR	alexandrin	PPP1CC
TR	eleutheroside_A	PPP1CC
CH	caprylic_acid	TLR4
CH	heptanoic_acid	TLR4
CH	hexadecanoic_acid	TLR4
CH	p-isopropylbenzoic_acid	BCL2
CH	p-isopropylbenzoic_acid	PTGS2
CH	pelargonic_acid	TLR4
CX	arachic_acid	TLR4
DG	galacturonic_acid	SRC
DG	glucuronic_acid	SRC
DS	ferruginol	PPP2CA
DS	ferruginol	PPP2CB
DS	ferruginol	PRKCA
DS	ferruginol	PRKCB
DS	tanshinaldehyde	PIK3CA
DS	tanshinaldehyde	PIK3R1
GC	Glyyunnanprosapogenin_D	PPP1CC
GC	Isoramanine	NOS2
GC	Methyl_2-hydroxy-3,4..	IKBKB
GC	Methyl_2-hydroxy-3,4..	NFKB1
GC	Methyl_2-hydroxy-3,4..	PRKAA1
GC	Methyl_2-hydroxy-3,4..	PTGS2
GC	Methyl_2-hydroxy-3,4..	TP53
HH	arachic_acid	TLR4
HH	dopa	PRKACA
NX	galacturonic_acid	SRC
NX	glucuronic_acid	SRC

Table S5 A total of 41 pairs of QXHC candidate targets and the corresponding chemical components delivered into docking

**Chemical_components**	**Major_targets**
caprylic_acid	ABL1
caprylic_acid	PRKACA
caprylic_acid	TLR4
heptanoic_acid	ABL1
heptanoic_acid	PRKACA
heptanoic_acid	TLR4
hexadecanoic_acid	ABL1
hexadecanoic_acid	PRKACA
hexadecanoic_acid	TLR4
pelargonic_acid	ABL1
pelargonic_acid	PRKACA
pelargonic_acid	TLR4
salicylic_acid	PTGS2
Linoleic_acid	PTGS2
arachic_acid	ABL1
arachic_acid	PRKACA
arachic_acid	TLR4
arginine	ASS1
arginine	NOS2
arginine	NOS3
glutamic_acid	EPRS
glutamic_acid	GLUD1
glutamic_acid	GLUD2
glutamic_acid	GLUL
glutamic_acid	GOT1
glutamic_acid	GRIA1
glutamic_acid	GRIN2A
glutamic_acid	GRIN2B
glycine	GARS
glycine	GRIN2A
lysine	KARS
molalanine	AARS

phenylalanine	CRH
phosphatidylserine	PRKCA
proline	EPRS
serine	SARS
tryptophane	WARS
tyrosine	YARS
ephedrine	TNF
pseudoephedrine	TNF
